# Supplementary material for: Associations of general and central adiposity with hypertension and cardiovascular disease among South Asian populations: a systematic review and meta-analysis
Source: BMJ Open. 2023 Dec 17;13(12):e074050. doi: 10.1136/bmjopen-2023-074050 (PMC10749025; doi:10.1136/bmjopen-2023-074050)
Supplement: Supplementary data [file bmjopen-2023-074050supp001.pdf]

DATA SUPPLEMENT

Associations of General and Central Adiposity with Hypertension and Cardiovascular Disease Among South Asian Populations: A Systematic Review and Meta-Analysis

Federica RE[1] MSc, Ayodipupo S. OGUNTADE[1] MSc, Bastian BOHRMANN[1] MSc, Fiona BRAGG[1,2] DPhil, Jennifer L. CARTER[1] PhD

1. Clinical Trial Service Unit and Epidemiological Studies Unit (CTSU), Nuffield Department of Population Medicine, University of Oxford, Oxford, UK.

2. MRC Population Health Research Unit, Nuffield Department of Population Health, University of Oxford, Oxford, UK

Address for correspondence
Dr Jennifer Carter
Nuffield Department of Population Health
Big Data Institute
Roosevelt Drive, Oxford
OX3 7LF, UK
jennifer.carter@ndph.ox.ac.uk

Re F, *et al. BMJ Open* 2023; 13:e074050. doi: 10.1136/bmjopen-2023-074050

CONTENTS

Data Supplement S1. Search strategy in MEDLINE and Embase…………..……………....3

Data Supplement S2. Summary of studies included in literature review looking at the association of measures of adiposity with blood pressure (BP) and hypertension (HTN)……………………………………………………………………………………..6

Data Supplement S3. Summary of studies included in literature review looking at the association of measures of adiposity with cardiovascular disease (CVD) risk and mortality………………………………………………………………………………………12

Data Supplement S4a/S4b/S4c. Quality assessment of included studies………..……15

Data Supplement S5. Random and fixed effects model formulas.………..……..…..…..17

Data Supplement S6. Mean change in systolic blood pressure (SBP, panel B) and diastolic blood pressure (DBP, panels A and C) per 5kg/m^2 higher body mass index (BMI)……………………………………………………………………………...………..18

Data Supplement S7. Mean change in systolic blood pressure (SBP, panel B) and diastolic blood pressure (DBP, panels A and C) per 10cm larger waist circumference (WC)…………………………………………………………………………………………..19

Data Supplement S8. Odds ratio (OR) of hypertension per 5kg/m^2 higher body mass index (BMI, A), 10cm larger waist circumference (WC, B) and 0.1-units larger waist-to-hip ratio (WHR, C)……………………………………………………………….…….20

Data Supplement S9. Odds ratio of cardiovascular disease for overweight vs. normal body mass index (BMI, panel A), large vs. normal waist circumference (WC, panel B), and large vs. normal waist-to-hip ratio (WHR, panel C)…………………………………...21

Data Supplement S10. Additional details on sensitivity analyses results………...…….22

Re F, *et al. BMJ Open* 2023; 13:e074050. doi: 10.1136/bmjopen-2023-074050

Data Supplement S1. Search strategy for MEDLINE and Embase.

Medline (Ovid MEDLINE® Epub Ahead of Print, In-Process & Other Non-Indexed Citations, Ovid MEDLINE® Daily and Ovid MEDLINE®) search strategy

1 Anthropometry/ or body composition/ or body fat distribution/ or body weight/ or overweight/ or thinness/ or Adipose Tissue/ or Adiposity/ or body fat distribution/ or body mass index/ or body size/ or waist-hip ratio/ or Obesity/ or abdominal fat/ or intra-abdominal fat/ or subcutaneous fat, abdominal/ or subcutaneous fat/ or Waist Circumference/ or Waist-Height Ratio

2 ((abdom* or intraabdom* or central or truncal or trunk or visceral or android or gynoid) adj fat?)

3 (central adiposity or obes* or fat distribution or waist circumference or hip circumference or waist-hip ratio or waist-to-hip ratio or waist-height ratio or waist-to-height ratio or waist-to-stature ratio or WHR or WHtR or electric impedance or bio-impedance or bio-electric impedance or sagittal abdominal diameter)

4 (anthropometr* or adipos* or body weight or body mass index or quetelet index or BMI or fat mass* or body fat* or fatness or lean mass or fat-free mass or overweight or body composition or body size or skeletal mass or muscle mass)

5 exp Coronary Disease/ or exp Myocardial Ischemia/ or Myocardial Infarction/ or exp Peripheral Vascular Diseases/ or exp Cerebrovascular Disorders/ or Cardiovascular Diseases/ or exp Hypertension/ or Blood Pressure/

6 (coronary heart disease or coronary artery disease or angina* or myocardial infarction or coronary isch?emia or myocardial isch?emia or heart attack* or isch?emic heart disease or CHD or stroke* or cerebrovascular accident or cerebrovascular disorder or cerebrovascular disease or peripheral arter* disease or peripheral vascular disease or cardiovascular disease or CVD or cardiovascular risk or vascular risk or vascular disease or hypertension or HTN or blood pressure)

7 exp case-control studies/ or exp cohort studies/ or exp cross-sectional studies/ or clinical trial/ or observational study/ or Randomized Controlled Trials as Topic/

8 (cohort* or longitudinal* or prospective* or follow-up* or observational * or inciden* or case-control or retrospective or cross-sectional or population-based or randomi?ed controlled trial* or randomi?ed trial* or clinical trial* or association* or risk*)

9 (South Asia* or Afghan* or Bangladesh*or Bhutan* or India* or Maldiv* or Nepal* or Pakistan* or Sri Lanka*)

10 bangladesh/ or bhutan/ or india/ or afghanistan/ or nepal/ or pakistan/ or sri lanka/

Re F, *et al. BMJ Open* 2023; 13:e074050. doi: 10.1136/bmjopen-2023-074050

11 1 or 2 or 3 or 4
12 5 or 6
13 7 or 8
14 9 or 10
15 11 and 12 and 13 and 14
16 limit 15 to (english language and humans and yr="1990-Current" and "all adult (18 plus years)")

Embase search strategy

1 Anthropometry/ or body composition/ or body fat distribution/ or body weight/ or overweight/ or thinness/ or Adipose Tissue/ or Adiposity/ or body fat distribution/ or body mass index/ or body size/ or waist-hip ratio/ or Obesity/ or abdominal fat/ or intra-abdominal fat/ or subcutaneous fat, abdominal/ or subcutaneous fat/ or Waist Circumference/ or Waist-Height Ratio/

2 ((abdom* or intraabdom* or central or truncal or trunk or visceral or android or gynoid) adj fat?)

3 (central adiposity or obes* or fat distribution or waist circumference or hip circumference or waist-hip ratio or waist-to-hip ratio or waist-height ratio or waist-to-height ratio or waist-to-stature ratio or WHR or WHtR or electric impedance or bio-impedance or bio-electric impedance or sagittal abdominal diameter)

4 (anthropometr* or adipos* or body weight or body mass index or quetelet index or BMI or fat mass* or body fat* or fatness or lean mass or fat-free mass or overweight or body composition or body size or skeletal mass or muscle mass)

5 exp Coronary Disease/ or exp Myocardial Ischemia/ or Myocardial Infarction/ or exp Peripheral Vascular Diseases/ or exp Cerebrovascular Disorders/ or Cardiovascular Diseases/ or exp Hypertension/ or Blood Pressure/

6 (coronary heart disease or coronary artery disease or angina* or myocardial infarction or coronary isch?emia or myocardial isch?emia or heart attack* or isch?emic heart disease or CHD or stroke* or cerebrovascular accident or cerebrovascular disorder or cerebrovascular disease or peripheral arter* disease or peripheral vascular disease or cardiovascular disease or CVD or cardiovascular risk or vascular risk or vascular disease or hypertension or HTN or blood pressure)

7 exp case-control studies/ or exp cohort studies/ or exp cross-sectional studies/ or clinical trial/ or observational study/ or Randomized Controlled Trials as Topic/

8 (cohort* or longitudinal* or prospective* or follow-up* or observational * or inciden* or case-control or retrospective or cross-sectional or population-based or randomi?ed controlled trial* or randomi?ed trial* or clinical trial* or association* or risk*)

Re F, *et al. BMJ Open* 2023; 13:e074050. doi: 10.1136/bmjopen-2023-074050

9 (South Asia* or Afghan* or Bangladesh*or Bhutan* or India* or Maldiv* or Nepal* or Pakistan* or Sri Lanka*)

10 bangladesh/ or bhutan/ or india/ or afghanistan/ or nepal/ or pakistan/ or sri lanka/

11 1 or 2 or 3 or 4
12 5 or 6
13 7 or 8
14 9 or 10
15 11 and 12 and 13 and 14
16 limit 15 to (human and english language and yr="1990-Current" and (adult >18 to 64 years or aged >65+ years))

Re F, *et al. BMJ Open* 2023; 13:e074050. doi: 10.1136/bmjopen-2023-074050

Data Supplement S2. Summary of studies included in literature review looking at the association of measures of adiposity with blood pressure (BP) and hypertension (HTN)

Results report associations in terms of mean change (mmHg) in BP or odds ratios (OR) of HTN. Additionally, 95% confidence intervals (95%CI) are reported where available. Unless otherwise specified, cut-offs used throughout for different measures of adiposity are: underweight BMI=<18.5kg/m^2, normal BMI=18.6-25.9kg/m^2, overweight BMI=26-29.9kg/m^2, obese BMI=≥30kg/m^2, low-risk/normal WC females=<80cm, high-risk/large WC females=≥80cm, low-risk/normal WC males=<94cm, high-risk/large WC males=≥94cm, low-risk/normal WHR females=<0.85cm, high-risk/large WHR females=≥0.85cm, low-risk/normal WHR males=<0.90cm, high-risk/large WHR males=≥90cm.
Abbreviations: BMI= body mass index (kg/m^2); WHR=waist-to-hip ratio (cm); WC=waist circumference (cm); SBP=systolic blood pressure (mmHg); DBP=diastolic blood pressure (mmHg); WHtR=waist-to-height ratio (cm); B=beta coefficient; t=two-tailed t-test.

Author (study design)	Geographical location	No. of participants (% of women)	Mean (SD) age, years	Adiposity measurements	Mean follow-up, years	Outcomes	Variables adjusted for	Results
Agrawal et al., 2014[1] (prospective cohort)	India	325 (100)	38 (N/A)	BMI, WHR	4	HTN	Age-group, education, religion, caste, employment status, standard of living	The likelihood of HTN was higher among obese women (OR: 3.86; p<0.0001) compared to women with a normal BMI. The likelihood of HTN among women with large WHR was 2.5 times higher (OR=2.47; p=0.002).
Bose et al., 2005[2] (cross-sectional)	India	150 (0)	40.7 (15.2)	BMI, WC	N/A	SBP, DBP, mean arterial pressure	Age	Regression of BMI and WC with SBP (B= 0.275, t=1.426), DBP (B= 0.009, t=0.067) and mean arterial pressure (B=0.098, t=0.065) showed that BMI did not have a significant impact on SBP, DBP, or mean arterial pressure. WC had a significant impact on SBP (B=0.506, t= 7.068), DBP (B=0.393, t=5.190) and mean arterial pressure (B=0.461, t=6.387).
Deshpande-Joshi et al., 2017[3] (cross-sectional)	India	140 (0)	32.4 (2)	BMI, WC, WHR	N/A	SBP, DBP, HTN	Not provided	Odds of HTN were highest for subjects in the highest tertile of BMI (OR: 18.9; 95% CI: 4.1–87.3) followed by large WC (OR: 11.4; 95% CI:1.6–13.0), and large WHR (OR: 6.6; 95% CI: 2.2–19.7).

Author (study design)	Geographical location	No. of participants (% of women)	Mean (SD) age, years	Adiposity measurements	Mean follow-up, years	Outcomes	Variables adjusted for	Results
Dhall et al., 2018[4] (cross-sectional)	India	568 (56.0)	32 (median age)	BMI, WC, WHR	N/A	SBP, DBP, HTN	Not provided	For males, large WC was significantly associated with risk of HTN (OR: 6.76; 95% CI: 2.57-17.76). Odds of HTN were found to be highest among those with risk category of WHR and conicity index in females. However, no significant association was observed.
Ganguli et al., 2013[5] (cross-sectional)	India	415 (100)	54.5 (9.4)	BMI, WC, WHR	N/A	HTN	Age, total cholesterol, exercise frequency, family history of HTN	WC was significantly associated with HTN (OR: 2.55; 95%CI: 1.07-6.06). WHR was significantly associated with HTN in the unadjusted (OR: 2.26, 95%CI: 1.39-3.68) and age-adjusted models (OR: 2.17, 95%CI: 1.31-3.59). BMI was not significantly associated with HTN in the unadjusted, age-adjusted, or multivariable-adjusted models.
Ghosh et al., 2007[6] (cross-sectional)	India	180 (N/A)	45.7 (9.3)	BMI, WC, WHR, WHtR	N/A	SBP, DBP, HTN	Age	Large WC was significantly associated with HTN (OR: 1.07; 95%CI: 1.0-1.12), along with overweight BMI (OR: 1.17; 95%CI: 1.04-1.32), large WHR (OR: 1.09; 95%CI: 1.01-1.17), and large WHtR (OR: 1.12; 95%CI: 1.03-1.22).
Hazarika et al., 2004[7] (cross-sectional)	India	3180 (54.7)	Partici-pants aged >30	BMI, WHR	N/A	HTN	Age, sex, marital status, type of work, alcohol, smoking, tobacco chewing	Overweight and BMI were significantly associated with HTN (OR overweight: 1.95; 95%CI: 1.37-2.78 and OR obese: 3.10; 95%CI: 1.17-8.22). Large WHR was also associated with HTN (OR: 1.54; 95%CI: 1.25-1.90).

Author (study design)	Geographical location	No. of participants (% of women)	Mean (SD) age, years	Adiposity measurements	Mean follow-up, years	Outcomes	Variables adjusted for	Results
Islam et al., 2016[8] (cross-sectional)	Bangladesh	3104 (65.0)	Not reported	BMI, WC	N/A	HTN	Age, education, diabetes	Among both males and females, BMI was significantly associated with mild, moderate, and severe hypertension. The same pattern followed for quartiles of WC, though associations appeared stronger than for BMI *(see full-text, Table 3 for details)*.
Kaur et al., 2008[9] (cross-sectional)	India	2148 (0)	40.5 (11.6)	BMI, WC, WHR, WHtR		SBP, DBP, HTN	Age	All four anthropometric indices showed a significant unadjusted OR for HTN across the quintiles. BMI and WC showed significant age-adjusted OR in all quintiles. WC showed a significant increase in age and BMI adjusted OR in the last two quintiles (*see full-text for quintiles).*
Kaur et al., 2012[10] (cross-sectional)	India	10463 (53.2)	Not reported	BMI, WC		HTN	Age	Odds of HTN were higher in the general obesity category (BMI ≥27.50kg/m^2) compared to the highest central obesity category (WC ≥80cm), OR BMI: 3.25; 95%CI: 2.57-4.11 and OR WC: 2.51; 95%CI: 2.12-2.98.
Khanam et al., 2019[11] (cross-sectional)	Bangladesh	1810 (52.3)	Male median: 50 (IQR 42–60) Female median: 47 (IQR 40–57)	BMI, WC		HTN	Age, education, wealth status, smoking, fruit and vegetables (BMI models adjustments unavailable)	Compared to healthy weight, overweight BMI was significantly associated with greater risk of HTN in both males (OR: 2.9; 95%CI: 1.8-4.6) and females (OR: 1.6; 95%CI: 1.1-2.4). Odds of HTN were fourfold higher among males (OR: 4.0; 95%CI: 2.5-6.4) and threefold higher among females (OR: 2.8; 95%CI: 2.0-4.1) with large WC.
Meshram et al., 2012[12] (cross-sectional)	India	4193 (54.9)	43.3 (14.9) for males and 42.4 (14.4) for females	BMI, WC	N/A	HTN	Age, sex, education, wealth index, alcohol	Large WC and overweight BMI were associated with two-fold (OR for abdominal obesity=1.7; 95%CI=1.24-2.33, OR for

Author (study design)	Geographical location	No. of participants (% of women)	Mean (SD) age, years	Adiposity measurements	Mean follow-up, years	Outcomes	Variables adjusted for	Results
								BMI=1.7; 95%CI=1.35-2.21) increase in risk of HTN.
Meshram et al., 2016[13] (cross-sectional)	India	8969 (49.5)	38.2 (15.4)	BMI, WC, WHR	N/A	HTN	Age, sex, education, occupation	Overweight individuals had 2.1 times higher risk of HTN (2.17; 95%CI 1.68-2.81), while individuals with large WC had two times higher risk (OR 1.96; CI 1.61-2.42). Individuals with large WHR had 1.4 times higher risk of HTN (OR 1.35; 95%CI 1.13-1.62).
Shriraam et al., 2021[14] (cross-sectional)	India	502 (57.8)	55.1 (10.86)	BMI, WC	N/A	HTN	Age, sex, occupation, physical activity, diabetes	Being overweight was not significantly associated with increased risk of HTN (OR: 1.27; 95%CI: 0.76-2.13). However, being obese was (OR: 9.75; 95%CI: 2.06-46.19). WC was significantly associated with increased risk of HTN (OR:1.62; 95%CI: 1.07-2.48).
Simmons et al., 2021[15] (cross-sectional)	Bangladesh	8019 (53)	See cate-gories in full-text	BMI, WC, WHR, WHtR	N/A	HTN	Age, residence, education, fruits and vegetables, alcohol, physical activity	Risk of HTN was higher among females and males who were overweight (OR: 1.35; 95%CI: 1.23-1.62 and OR: 2.40; 95%CI: 2.34-2.62) and obese (OR: 1.68; 95%CI: 1.44-1.89 and OR: 2.62; 95%CI: 2.02-2.94). A moderate WC, defined as 94-102cm in males and 80-88cm in females, was associated with higher risk of HTN among both groups (OR females: 1.72; 95%CI: 1.64-1.91; OR males: 2.14; 95%CI: 2.05-2.28). A stronger pattern was observed for large WC (OR: 2.52; 95%CI: 2.05-2.98 and OR: 2.65; 95%CI: 2.34-2.85). Patterns were similar for WHR and WHtR.

Re F, *et al. BMJ Open* 2023; 13:e074050. doi: 10.1136/bmjopen-2023-074050

Author (study design)	Geographical location	No. of participants (% of women)	Mean (SD) age, years	Adiposity measurements	Mean follow-up, years	Outcomes	Variables adjusted for	Results
Singh et al., 2012[16] (cross-sectional)	India	3118 (36.5)	Not reported	BMI, WHR	N/A	HTN	Tobacco, alcohol	BMI was significantly associated with increased risk of HTN (OR: 1.52; 95%CI: 1.25-1.85), as was WHR (OR: 1.65; 95%CI: 1.36-2.00).
Taing et al., 2016[17] (cross-sectional)	India	7601 (50.1)	40 (11)	BMI, WC, HC, WHR, WHtR	N/A	SBP, DBP, mean arterial pressure	Education, location, WC, BMI	Every 5 kg/m^2 greater BMI or 10cm wider WC was associated with a 5 and 4mmHg higher SBP, and a 4 and 3mmHg higher DBP. The association between WC and DBP was stronger than the that of BMI and DBP.
Tselha et al., 2019[18] (cross-sectional)	India	214 (53.2)	34.0 (16.0) in males and 33.7 (11.4) in females	BMI, WC, WHR, WHtR	N/A	HTN	Not provided	In males, WHR followed by WC and BMI were found to be associated with HTN: OR: 1.48 (96%CI:-0.59-3.72); 1.16 (95%CI:-0.37-3.65); and 1.11 (95%CI:0.11-11.20). In females, WHtR, BMI and WHR were found to be the strongest predictors of HTN: OR: 6.82 (95%CI: -2.68-17.39); 1.31 (95%CI: - 0.33-5.30); and 1.06 (95%CI: 0.21-3.17), respectively.
Venkatramana et al., 2002[19] (cross-sectional)	India	212 (0)	Urban: 47.4 (9.1); rural: 40.8 (14.2)	BMI, WC	N/A	SBP, DBP	Age, smoking, physical activity	BMI did not have significant effects on SBP (B=0.050, p=0.891) and DBP (B=0.225, p=0.544) in the urban population. However, it did have significant effects in the rural population (SBP B=1.182, p<0.001; DBP B=1.047, p=0.005). WC did not have significant effects on SBP (B=0.224, p=0.660) or DBP (B=0.140, p=0.788) in the urban population or the rural population (SBP: B=-0.990, p=0.059; DBP: B=0.140, p=0.788).

Author (study design)	Geographical location	No. of participants (% of women)	Mean (SD) age, years	Adiposity measurements	Mean follow-up, years	Outcomes	Variables adjusted for	Results
Vikram et al., 2016[20] (cross-sectional)	India	509 (45.3)	40.0 (8.6) in males and 38.7 (9.2) in females	BMI, WC, WHR, WHtR	N/A	HTN	Crude	In males, being overweight (OR: 2.12; 95%CI 1.07-4.37), having a large WC (OR: 2.65; 95%CI: 1.33-5.26), WHR (OR: 2.27; 95%CI: 0.91-5.61), and WHtR (OR: 3.85; 95%CI: 1.57-9.43) was associated with HTN. In females, overweight BMI (OR: 4.00; 95%CI: 1.16-13.80) and large WC (OR: 5.25; 95%CI: 1.52-18.11) were significantly associated with HTN. Large WHR (OR: 4.21; 95%CI: 0.96-18.47) was not statistically significant.
Yadav et al., 2008[21] (cross-sectional)	India	1746 (31.8)	49.8 (11.5)	BMI, WC, WHR	N/A	HTN	Age, sex	Being overweight was associated with HTN (OR: 2.2; 95%CI: 1.5-3.1). Associations were stronger for large WC (OR: 4.1; 95%CI: 2.6-6.4), but weaker for large WHR (OR: 1.56; 95%CI: 1.37-1.79).

Data Supplement S3. Summary of studies included in literature review looking at the association of measures of adiposity with cardiovascular disease (CVD) risk and mortality

Results report associations in terms hazard ratios (HR), or relative risk (RR). Additionally, 95% confidence intervals (95%CI) and p-values (p) are reported where available. Unless otherwise specified, cut-offs used throughout for different measures of adiposity are: underweight BMI=<18.5kg/m^2, normal BMI=18.6-25.9kg/m^2, overweight BMI=26-29.9kg/m^2, obese BMI=≥30kg/m^2, low-risk/normal WC females=<80cm, high-risk/large WC females=≥80cm, low-risk/normal WC males=<94cm, high-risk/large WC males=≥94cm, low-risk/normal WHR females=<0.85cm, high-risk/large WHR females=≥0.85cm, low-risk/normal WHR males=<0.90cm, high-risk/large WHR males=≥90cm.
Abbreviations: BMI=body mass index (kg/m^2); WC=waist circumference (cm); WHR=waist-to-hip ratio (cm); CHD=coronary heart/artery disease; HC=hip circumference.

Author (study design)	Geographical location	No. of participants (% of women)	Mean (SD) age, years	Adiposity measurements	Mean follow-up, years	Outcomes	Adjustments	Results
Banerjee et al., 2021[22] (cross-sectional)	India	31464 (N/A)	N/A, participants >60	BMI, WC, WHR	N/A	CVD	Age, sex, education, marital status, working status, tobacco, alcohol, physical activity, monthly per-capital consumption expenditure, religion, caste, place of residence, region	Likelihood of CVD was higher among adults who were overweight (OR: 1.60; CI: 1.48–1.72), had a large WC (OR: 1.50; CI: 1.39–1.62) and high-risk WHR (AOR: 1.34; CI: 1.25–1.44), compared to adults with normal BMI and those who do not have a high-risk WC and high risk WHR.
Bodkhe et al., 2019[23] (cross-sectional)	India	1190 (51)	N/A, participants >60	BMI, WC, WHR	N/A	CHD	Age, sex, education, socioeconomic status, physical activity, HTN, diabetes, tobacco	No significant associations between risk of CHD and obesity (OR for BMI: 1.64; 95%CI: 0.71-3.77; OR for WC: 0.72; 95%CI: 0.28-1.87; OR for WHR: 0.96; 95%CI: 0.54-1.71).
Bramhankar et al., 2021[24] (cross-sectional)	India	37536 (N/A)	N/A, participants >45	BMI, WC, WHR	N/A	CVD	Age, sex, education, employment, residence, caste, wealth quintile, region, smoking, alcohol	Adults were 2.3 times more likely (OR: 2.33; 95%CI: 2.2-2.5) by obesity, 61% more likely (OR: 1.61; 95%CI: 1.63-1.63) by high-risk WHR and 98% more likely (OR: 1.98; 95%CI: 1.9-2.1) by high-risk WC to develop CVD than their normal-weight individuals.

Author (study design)	Geographical location	No. of participants (% of women)	Mean (SD) age, years	Adiposity measurements	Mean follow-up, years	Outcomes	Adjustments	Results
Cameron et al., 2012[25] (prospective cohort)	Mauritius (South Asian Mauritius and Africa Mauritius reported separately)	7,979	Not reported	BMI, WC, HC	15.1	Death from CVD	Age, sex, smoking	In the South Asian population, high-risk WC was significantly related to CVD death in males (OR: 1.8; 95%CI: 1.2-2.7) and females (OR: 1.5; 95%CI: 1.1-2.2), compared with those below the obesity cut-off, when adjusting for hip circumference.
Latheef et al., 2018[26] (case-control)	India	165 (0)	Not reported	WC, WHR	N/A	CAD	Not provided	Association of anthropometric variables with CAD: WC- OR: 1.16; 95%CI: 1.08-1.24. WHR- OR: 42.69; 95%CI: 10.28-177.20.
Nishtar et al., 2008[27] (cross-sectional)	Pakistan	400 (16)	Cases: 51.2 (9.5) Controls: 48.2 (9.5)	BMI, WC, WHR	N/A	CAD	Age, urban/rural, family history of CAD, smoking, diabetes, HTN, BP	A significant association of WC (OR: 1.02; 95%CI: 1.00-1.04) and WHR (OR: 1.06; 95%CI: 1.03-1.10) was found with CAD. The association was not significant for BMI (OR: 1.02; 95%CI: 0.96-1.08).
Patil et al., 2004[28] (case-control)	India	Cases 111 (20.7) Controls 222 (20.7)	*See categories in full-text*	BMI, WHR	N/A	Acute myocardial infarction	Crude	Large WHR was associated with increased risk of acute MI (OR: 2.50; 95%CI: 1.52-4.10). Overweight BMI, was also associated with increased risk (OR: 1.55; 95%CI: 0.88-2.69) but was not significant.

Author (study design)	Geographical location	No. of participants (% of women)	Mean (SD) age, years	Adiposity measurements	Mean follow-up, years	Outcomes	Adjustments	Results
Prabhakar et al., 2020[29] (case-control)	India	Cases: 144 (30.5) Controls: 144 (50.0)	*See categories in full-text*	BMI, WC	N/A	Stroke	Age, sex, smoking, alcohol, BP, diabetes, total cholesterol, HDL	Large WC in both men and women were not significantly associated with increased risk of stroke (OR: 1.33; 95%CI: 0.69-2.59 and OR: 1.06; 95%CI: 0.33-3.37 respectively). Being overweight was also not significantly associated with stroke (OR: 1.06; 95%CI: 0.33-3.37).
Shah et al., 2017[30] (case-control)	India	300 (22.0)	56.1 (10.7)	BMI, WC, WHR	N/A	CAD	Not provided	Overweight BMI significantly associated with risk of CAD (OR: 2.65; 95%CI: 1.49-4.72). Large WHR not significantly associated with risk of CAD (OR: 1.60; 95%CI: 0.97-2.66). High WC significantly associated with risk of CAD (OR: 1.71; 95%CI: 1.01-2.88).

Data Supplement S4a, S4b, and S4c. Quality assessment of included studies

Performed using the Newcastle-Ottawa Scale adapted to cohort, case-control, and cross-sectional studies. Criteria that were met were marked with a 1, whereas criteria that were not met were marked with a 0.

S4a: COHORT STUDIES

First author, publication year	SELECTION				COMPARABILITY		OUTCOME			Total (9/9)
	Representative of exposed cohort	Selection of non-exposed	Exposure ascertainment	Outcome absent at start	Main factor controlled for	Additional factors controlled for	Assessment of outcomes	Sufficient follow-up time (>2 years)	Adequacy of follow-up	
Agrawal et al., 2014	1	1	1	0	1	1	1	0	1	6/9
Cameron et al., 2012	1	1	1	1	1	0	0	1	1	7/9

S4b: CASE-CONTROL STUDIES

First author, publication year	SELECTION				COMPARABILITY		OUTCOME			Total (9/9)
	Adequate case definition	Representativeness of cases	Selection of controls	Definition of controls	Main factor controlled for	Additional factors controlled	Ascertainment of exposure	Same ascertainment cases and controls	Non-response rate	
Latheef et al., 2018	1	0	0	1	1	0	1	1	0	5/9
Patil et al., 2004	1	1	0	1	1	0	1	1	0	6/9
Prabhakar et al., 2020	1	0	1	1	1	0	1	1	0	6/9
Nishtar et al., 2008	1	0	0	1	1	0	1	1	0	5/9
Shah et al., 2017	1	0	0	1	1	0	0	1	0	4/9

Re F, *et al. BMJ Open* 2023; 13:e074050. doi: 10.1136/bmjopen-2023-074050

S4c: CROSS-SECTIONAL STUDIES

First author, publication year	SELECTION				COMPARABILITY		OUTCOME		Total (10/10)
	Representativeness of sample	Sample size	Ascertainment of exposure (max 2)	Non-response rate	Main factor controlled for	Additional factors controlled	Assessment of outcome (max 2)	Same ascertainment cases and controls	
Banerjee et al., 2021	1	1	0	0	1	1	1	1	6/10
Bodkhe et al., 2019	1	0	2	0	1	1	1	1	7/10
Bose et al., 2005	1	0	2	0	1	0	2	1	7/10
Bramhankar et al., 2021	1	0	0	0	1	1	1	1	5/10
Deshpande-Joshi et al., 2017	0	1	2	0	0	0	2	1	6/10
Dhall et al., 2018	0	0	2	0	0	0	2	1	5/10
Ganguli et al., 2013	1	0	2	0	1	1	2	1	8/10
Ghosh et al., 2007	0	0	2	0	0	0	2	1	5/10
Hazarika et al., 2004	1	1	2	0	1	1	2	1	9/10
Islam et al., 2016	1	1	2	0	1	1	2	1	9/10
Kaur et al., 2008	0	0	2	0	0	0	2	1	5/10
Kaur et al., 2012	0	1	2	0	1	0	2	1	7/10
Khanam et al., 2019	1	1	2	0	1	1	2	1	9/10
Meshram et al., 2012	0	1	2	0	1	1	2	1	8/10
Meshram et al., 2016	1	1	2	0	1	1	2	1	9/10
Shritaam et al., 2021	1	1	2	0	1	1	2	1	9/10
Simmons et al., 2021	0	0	1	0	1	1	1	1	5/10
Singh et al., 2012	0	0	2	0	1	0	2	1	6/10
Taing et al., 2016	1	0	2	0	0	0	2	1	6/10
Tselha et al., 2019	0	0	2	0	0	0	2	1	5/10
Venkatramana et al., 2002	1	0	2	0	1	1	2	1	8/10
Vikram et al., 2016	1	0	2	0	0	0	2	1	6/10
Yadav et al., 2008	0	1	2	1	1	1	2	1	9/10

Re F, *et al. BMJ Open* 2023; 13:e074050. doi: 10.1136/bmjopen-2023-074050

Data Supplement S5. Random and fixed effects model formulas.[31–33]

Fixed-effects model formula

The fixed-effects model assumes that all included studies come from the same underlying population and that any variation in the study results is due to sampling error. In this model, the effect size is assumed to be the same for all studies, and any differences between study results are solely due to chance.

$$\theta_{FE} = \frac{\sum_{i=1}^{k} w_i \cdot \theta_i}{\sum_{i=1}^{k} w_i}$$

Where:

- θ_{FE} is the estimated overall effect size.
- k is the number of studies.
- w_i is the weight assigned to each study (based on sample size and precision).
- θ_i is the effect size estimate for each study.

Random-effects model formula

The random-effects model considers not only the within-study variability but also the between-study variability. It assumes that the true effect size can vary between studies due to both sampling error and real differences in effect size between the studies.

$\theta_{RE} = \frac{\sum_{i=1}^{k} w_i \cdot \theta_i}{\sum_{i=1}^{k} w_i}$ and $w_i = \frac{1}{(\tau^2 + \theta_i^2)}$

Where:

- θ_{RE} is the estimated overall effect size in the random-effects model.
- θ_i^2 is the within-study variance of each study.
- τ^2 is the estimated between-study variance, which represents the additional variability between studies.

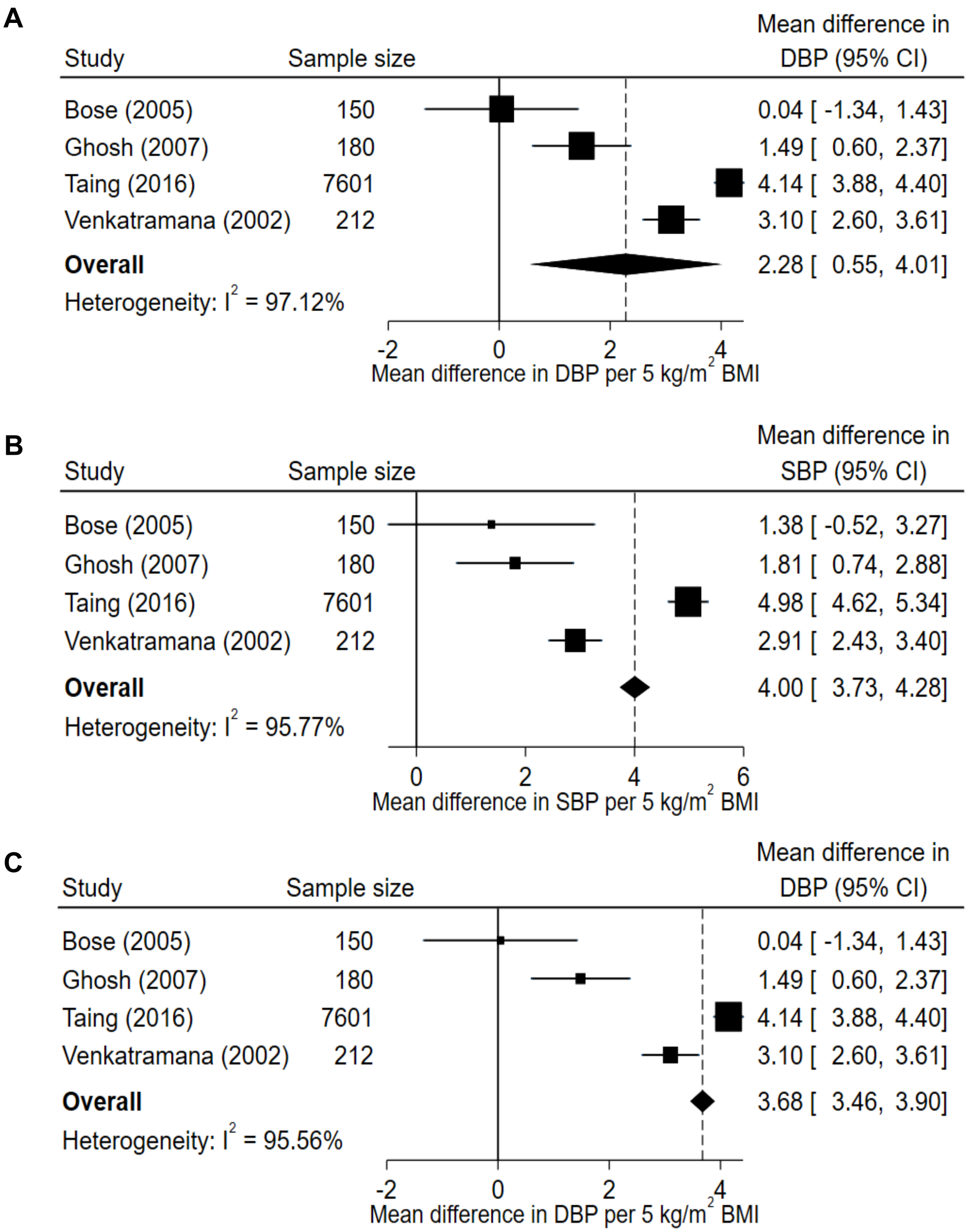

Data Supplement S6. Mean change in systolic blood pressure (SBP, panel B) and diastolic blood pressure (DBP, panels A and C) per 5kg/m^2 higher body mass index (BMI)
Random effects (A) and fixed-effects (B and C) models were applied on four studies reporting associations of blood pressure and BMI. The total number of participants was 8,143.
Abbreviations: 95% confidence interval (95%CI).

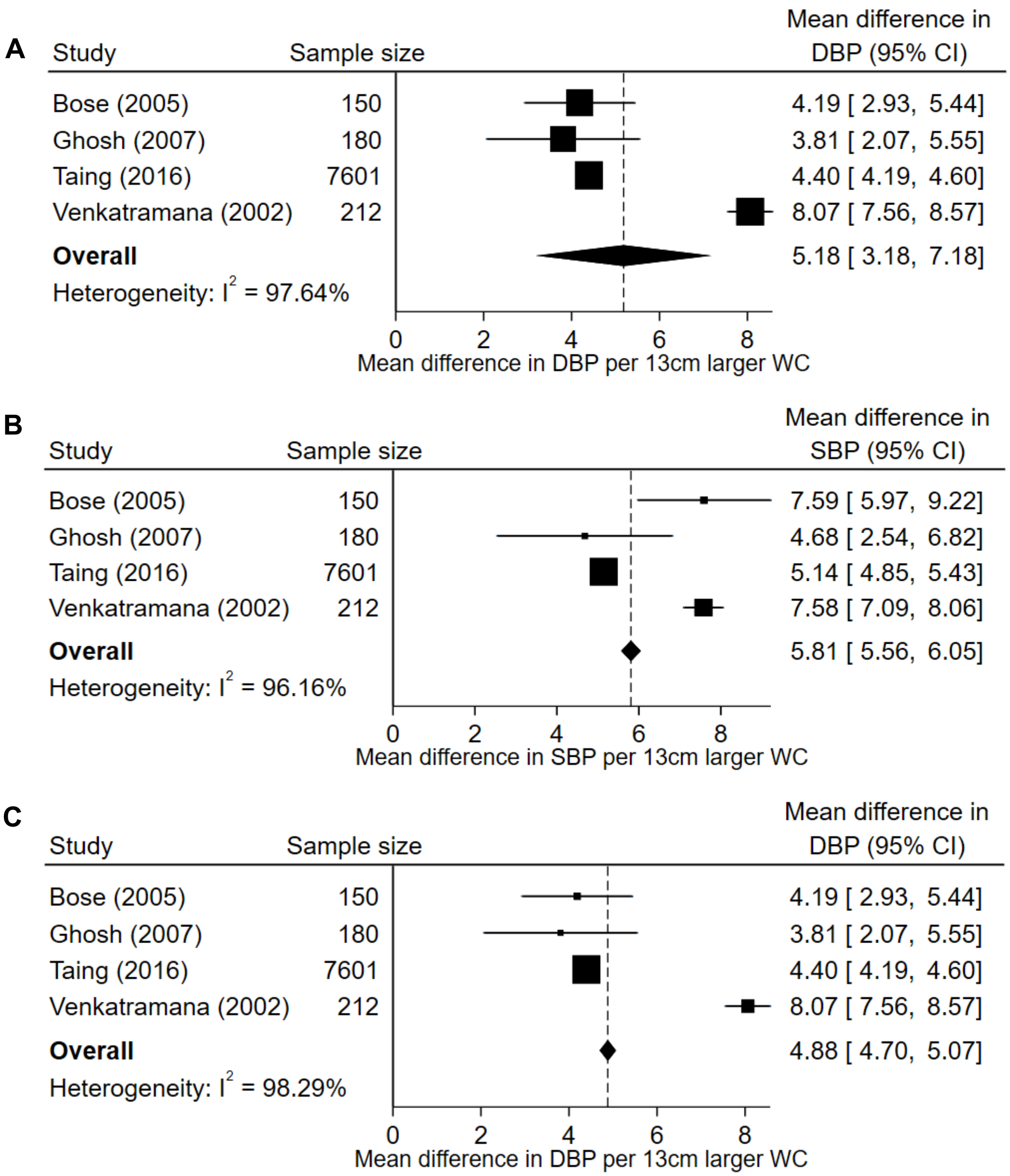

Data Supplement S7. Mean change in systolic blood pressure (SBP, panel B) and diastolic blood pressure (DBP, panels A and C) per 13cm larger waist circumference (WC)
Random effects (A) and fixed-effects (B and C) models were applied on four studies reporting associations of blood pressure and WC. The total number of participants was 8,143.
Abbreviations: 95% confidence interval (95%CI).

Re F, *et al. BMJ Open* 2023; 13:e074050. doi: 10.1136/bmjopen-2023-074050

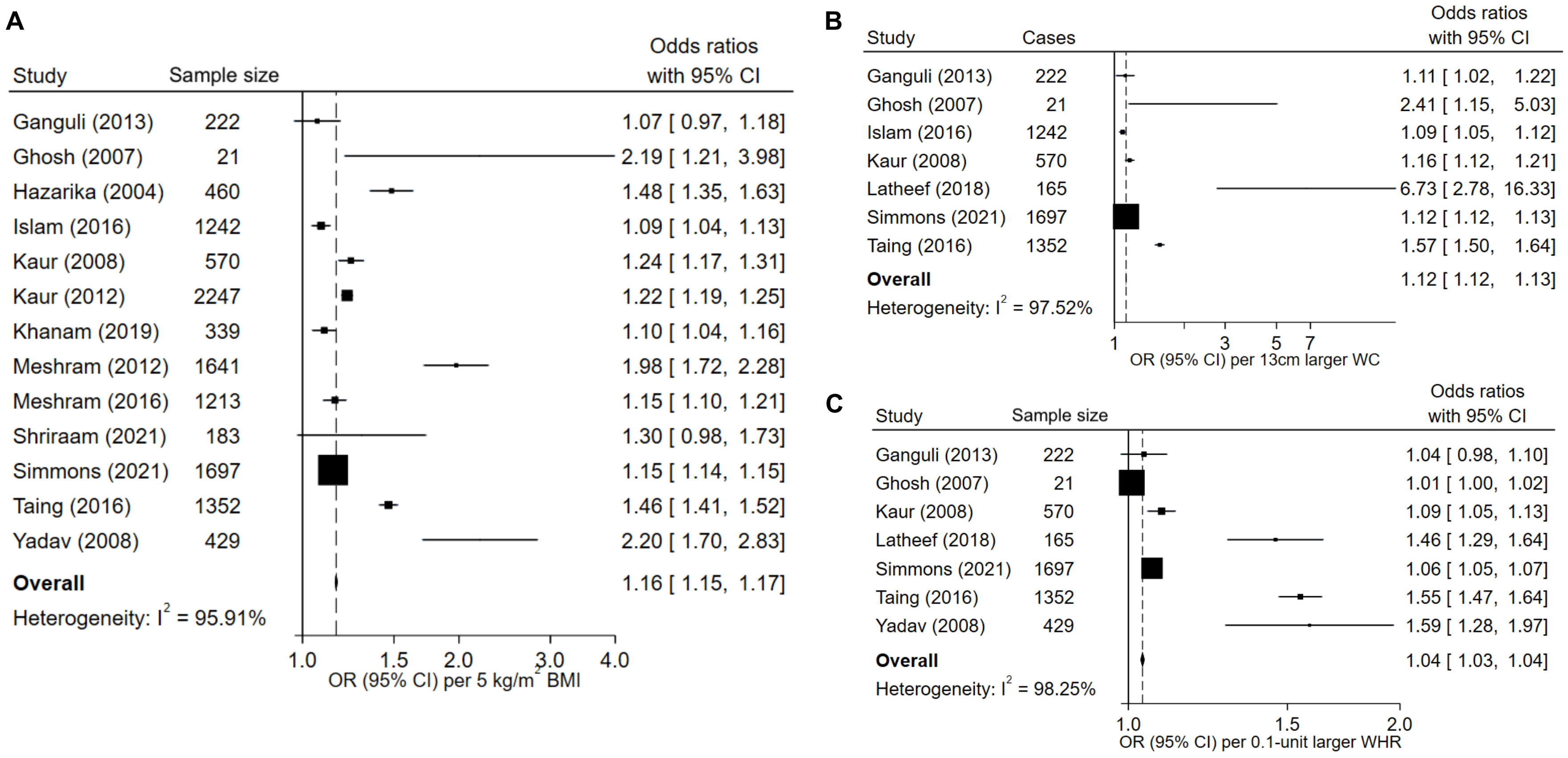

Data Supplement S8. Odds ratio (OR) of hypertension per 5kg/m² higher body mass index (BMI, A), 13cm larger waist circumference (WC, B) and 0.1-units larger waist-to-hip ratio (WHR, C)

A fixed-effects model was used on 13 studies looking at BMI, seven studies looking at WC and seven studies looking at WHR. The total number of participants was 11,636 for panel A, 4,899 for B, and 4,456 or panel C. *Abbreviations: 95% confidence interval (95%CI).*

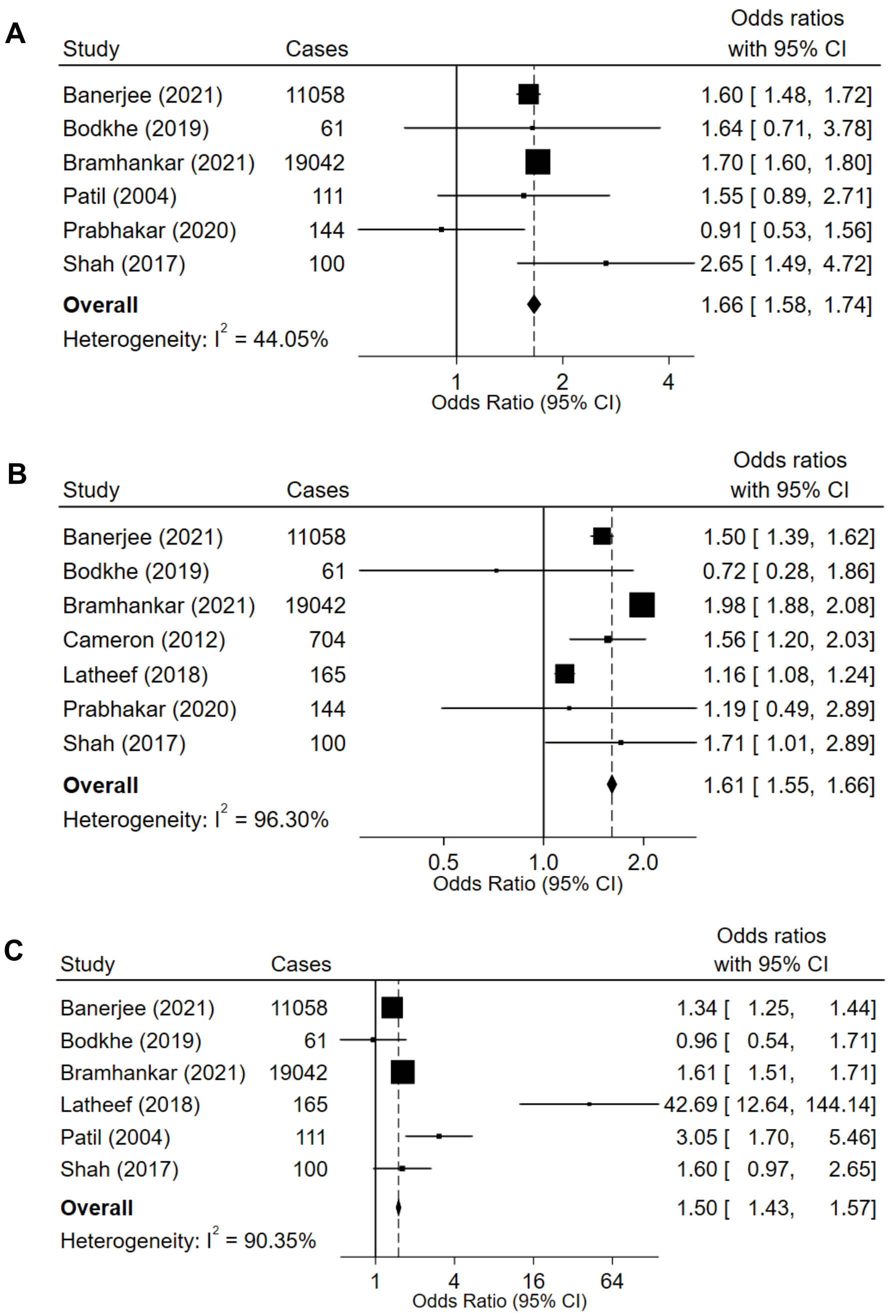

Data Supplement S9. Odds ratio of cardiovascular disease for overweight vs. normal body mass index (BMI, panel A), large vs. normal waist circumference (WC, panel B), and large vs. normal waist-to-hip ratio (WHR, panel C)

A fixed-effects model was used on six studies looking at BMI, seven studies looking at WC, and six studies looking at WHR. *Abbreviations: 95% confidence interval (95%CI).*

Re F, *et al. BMJ Open* 2023; 13:e074050. doi: 10.1136/bmjopen-2023-074050

Data Supplement S10. Additional details on sensitivity analyses results.

Results were not substantively different after sensitivity analyses. For the studies looking at the association of a 5kg/m^2 increase in BMI with HTN, removing the study by Meshram *et al*. decreased the odds ratio from 1.33 (95%CI: 1.18-1.51) to 1.28 (95%CI: 1.14-1.42).[12] Similar results were seen when removing the study by Yadav et al. (OR: 1.28; 95%CI: 1.15-1.43).[21] Individual removal of several other studies resulted in an increase in magnitude of associations to 1.36 (1.20-1.55).[5,8,11,13,15] For studies looking at the odds of HTN per 10cm larger WC, removing the study by Simmons et al. resulted in an increase in estimates from 1.33 (1.04-1.69) to 1.57 (1.28-1.93).[15] For studies looking at the odds of HTN per 0.1-unit larger WHR, removing the studies by Ghosh et al. [6] increased the strength of associations from 1.18 (1.02-1.37) to 1.26 (1.07-1.48).

Re F, *et al. BMJ Open* 2023; 13:e074050. doi: 10.1136/bmjopen-2023-074050

DATA SUPPLEMENT REFERENCE LIST

1. Agrawal P, Gupta K, Aboyans V, Agrawal S. Women's health in India: the role of body mass index. Health Care Women Int. 2014;36(3):320–41.

2. Bose K, Ghosh A, Roy S, Gangopadhyay S. The relationship of age, body mass index and waist circumference with blood pressure in Bengalee Hindu male jute mill workers of Belur, West Bengal, India. Anthropology Anz. 2005;63(2):205–12.

3. Deshpande-Joshi S, Rao S. Differential risk of hypertension among lean and non-lean rural subjects in relation to decadal changes in anthropometry. J Am Coll Nutr. 2018;37(5):380–6.

4. Dhall M, Devi K, Nilupher A, Gupta U, Tyagi R, Kapoor S. Hypertension and its correlate with general and central adiposity: A study among urban population of Delhi. Diabetes Metab Syndr. 2018;12(6):881–4.

5. Ganguli D, Das N, Saha I, Chaudhuri D, Ghosh S, Dey S. Risk factors for hypertension in a population-based sample of postmenopausal women in Kolkata, West Bengal, India. Asia Pac J Public Health. 2013;25(5):388–97.

6. Ghosh J, Bandyopadhyay A. Comparative evaluation of obesity measures: relationship with blood pressure and hypertension. Singapore Med J. 2007;48(3):232–5.

7. Hazarika N, Narain K, Biswas D, Kalita H, Mahanta J. Hypertension in the native rural population of Assam. Natl Med J India. 2004;17:300–4.

8. Islam FMA, Bhuiyan A, Chakrabarti R, Rahman MA, Kanagasingam Y, Hiller JE. Undiagnosed hypertension in a rural district in Bangladesh: The Bangladesh Population-based Diabetes and Eye Study (BPDES). J Hum Hypertens. 2016;30(4):252–9.

9. Kaur P, Radhakrishnan E, Sankarasubbaiyan S, Rao S, Kondalsamy-Chennakesavan S, Rao T, et al. A Comparison of Anthropometric Indices for Predicting Hypertension and Type 2 Siabetes in a Male Industrial Population of Chennai, South India. Ethnicity and Disease. 2008;18:31–26.

10. Kaur P, Rao S, Radhakrishnan E, Rajasekar D, Gupte M. Prevalence, awareness, treatment, control and risk factors for hypertension in a rural population in South India. Int J Public Health. 2012;57(1):87–94.

11. Khanam R, Ahmed S, Rahman S, Kibria G, Syed J, Khan A, et al. Prevalence and factors associated with hypertension among adults in rural Sylhet district of Bangladesh: a cross-sectional study. BMJ Open [Internet]. 2019 [cited 2022 Jul 24];9(10). Available from: https://bmjopen.bmj.com/lookup/doi/10.1136/bmjopen-2018-026722

12. Meshram II, Arlappa N, Balkrishna N, Rao KM, Laxmaiah A, Brahmam GNV. Prevalence of hypertension, its correlates and awareness among adult tribal population of Kerala state, India. J Postgrad Med. 2012;58(4):255–61.

13. Meshram II, Vishnu Vardhana Rao M, Sudershan Rao V, Laxmaiah A, Polasa K. Regional variation in the prevalence of overweight/obesity, hypertension and diabetes and their correlates among the adult rural population in India. Br J Nutr. 2016;115(7):1265–72.

14. Shriraam V, Mahadevan S, Arumugam P. Prevalence and risk factors of diabetes, hypertension and other non-communicable diseases in a tribal population in South India. Indian J Endocr Metab. 2021;25(4):313.

15. Simmons S, Hagan Jr. J, Schack T. The influence of anthropometric indices and intermediary determinants of hypertension in Bangladesh. IJERPH [Internet]. 2021 [cited 2022 Jul 24];18(11). Available from: https://www.mdpi.com/1660-4601/18/11/5646

16. Singh R, Mukherjee M, Kumar R, Singh R, Pal R. Study of risk factors of coronary heart disease in urban slums of Patna. Nepal J Epidemiology. 2012;2(3):205–12.

17. Taing K, Farkouh M, Moineddin R, Tu J, Jha P. Age and sex-specific associations of anthropometric measures of adiposity with blood pressure and hypertension in India: a cross-sectional study. BMC Cardiovasc Disord. 2016;16(1).

18. Tselha N, Shimrah C, Kulshreshtha M, Devi N. Association between hypertension and adiposity indicators: A study among the Muslim population of Uttar Pradesh. Diabetes Metab Syndr. 2019;13(4):2335–8.

19. Venkatramana P, Reddy P. Association of overall and abdominal obesity with coronary heart disease risk factors: comparison between urban and rural Indian men. Asia Pac J Clin Nutr. 2002;11(1):66–71.

20. Vikram N, Latifi A, Misra A, Luthra K, Bhatt S, Guleria R, et al. Waist-to-height ratio compared to standard obesity measures as predictor of cardiometabolic risk factors in Asian Indians in North India. Metab Syndr Relat Disord. 2016;14(10):492–9.

21. Yadav S, Boddula R, Genitta G, Bhatia V, Bansal B, Kongara S, et al. Prevalence & risk factors of pre-hypertension & hypertension in an affluent north Indian population. Indian J Med Res. 2008;(128):712–20.

22. Banerjee S, Kumar P, Srivastava S, Banerjee A. Association of anthropometric measures of obesity and physical Activity with ardiovascular diseases among older adults: evidence from a cross-sectional survey, 2017–18. Gaipov A, editor. PLoS ONE [Internet]. 2021 [cited 2022 Jul 24];16(12). Available from: https://dx.plos.org/10.1371/journal.pone.0260148

23. Bodkhe S, Jajoo S, Jajoo U, Ingle S, Gupta S, Taksande B. Epidemiology of confirmed coronary heart disease among population older than 60 years in rural central India—A community-based cross-sectional study. Indian Heart Journal. 2019;71(1):39–44.

24. Bramhankar M, Pandey M, Rana G, Rai B, Mishra N, Shukla A. An assessment of anthropometric indices and its association with NCDs among the older adults of India: evidence from LASI Wave-1. BMC Public Health. 2021;21(1):1357.

25. Cameron A, Magliano D, Shaw J, Zimmet P, Carstensen B, Alberti K, et al. The influence of hip circumference on the relationship between abdominal obesity and mortality. Intl J of Epi. 2012;41(2):484–94.

26. Latheef SAA, Subramanyam G, Reddy BM. Utility of anthropometric traits and indices in predicting the risk of coronary artery disease in the adult men of southern Andhra Pradesh. Indian Heart J. 2018;70(3):S133–9.

27. Nishtar S, Wierzbicki A, Lumb P, Lambert-Hammill M, Turner C, Crook M, et al. Waist-hip ratio and low HDL predict the risk of coronary artery disease in Pakistanis. Current Medical Research and Opinion. 2008;20(1):55–62.

28. Patil S, Joshi R, Gupta G, Reddy M, Pai M, Kalantri S. Risk factors for acute myocardial infarction in a rural population of central India: A hospital-based case–control study. Nat Med J India. 2004;17(4).

29. Prabhakar S, Suravarapu S, Mathai D, Renangi S, Challa S. Risk factors for stroke in rural population of Telangana State of India, an unmatched case-control study. J Neurosci Rural Pract. 2020;11(3):448–53.

30. Shah M, Mazumdar V, Patel S, Baxi R, Shringarpure K. A case control study of risk factors of coronary heart disease among patients admitted at tertiary hospital in western India. Australasian Medical Journal. 2017;10(05):381–8.

31. Borenstein M, Hedges L, Higgins J, Rothstein H. Introduction to Meta-Analysis. 2nd ed. John Wiley & Sons; 2009.

32. Higgins J, Green S, editors. Cochrane Handbook for Systematic Reviews of Interventions Verison 5.1.0 [Internet]. 2011. Available from: http://handbook-5-1.cochrane.org/.

33. DerSimonian R, Laird N. Meta-analysis in clinical trials. Controlled Clinical Trials. 1986;7(3):177–88.
