# Supplementary material for: Associations of general and central adiposity with hypertension and cardiovascular disease among South Asian populations: a systematic review and meta-analysis
Source: BMJ Open. 2023 Dec 17;13(12):e074050. doi: 10.1136/bmjopen-2023-074050 (PMC10749025; doi:10.1136/bmjopen-2023-074050)
Supplement: Author's manuscript [file bmjopen-2023-074050.draft_revisions.pdf]

BMJ Open

BMJ Open is committed to open peer review. As part of this commitment we make the peer review history of every article we publish publicly available.

When an article is published we post the peer reviewers' comments and the authors' responses online. We also post the versions of the paper that were used during peer review. These are the versions that the peer review comments apply to.

The versions of the paper that follow are the versions that were submitted during the peer review process. They are not the versions of record or the final published versions. They should not be cited or distributed as the published version of this manuscript.

BMJ Open is an open access journal and the full, final, typeset and author-corrected version of record of the manuscript is available on our site with no access controls, subscription charges or pay-per-view fees (http://bmjopen.bmj.com).

If you have any questions on BMJ Open's open peer review process please email info.bmjopen@bmj.com

BMJ Open

Associations of General and Central Adiposity with Hypertension and Cardiovascular Disease Among South Asian Populations: A Systematic Review and Meta-Analysis

Journal:	*BMJ Open*
Manuscript ID	bmjopen-2023-074050
Article Type:	Original research
Date Submitted by the Author:	25-Mar-2023
Complete List of Authors:	Re, Federica; University of Oxford, Clinical Trial Service Unit and Epidemiological Studies Unit (CTSU), Nuffield Department of Population Health<br>Oguntade, Ayodipupo; University of Oxford, Clinical Trial Service Unit and Epidemiological Studies Unit (CTSU), Nuffield Department of Population Health<br>Bohrmann, Bastian; University of Oxford, Clinical Trial Service Unit and Epidemiological Studies Unit (CTSU), Nuffield Department of Population Health<br>Bragg, Fiona; University of Oxford, Clinical Trial Service Unit and Epidemiological Studies Unit (CTSU), Nuffield Department of Population Health; University of Oxford, MRC Population Health Research Unit, Nuffield Department of Population Health<br>Carter, Jennifer ; University of Oxford, Clinical Trial Service Unit and Epidemiological Studies Unit (CTSU), Nuffield Department of Population Health; University of Oxford, MRC Population Health Research Unit, Nuffield Department of Population Health
Keywords:	Systematic Review, Hypertension < CARDIOLOGY, EPIDEMIOLOGY

SCHOLARONE™
Manuscripts

I, the Submitting Author has the right to grant and does grant on behalf of all authors of the Work (as defined in the below author licence), an exclusive licence and/or a non-exclusive licence for contributions from authors who are: i) UK Crown employees; ii) where BMJ has agreed a CC-BY licence shall apply, and/or iii) in accordance with the terms applicable for US Federal Government officers or employees acting as part of their official duties; on a worldwide, perpetual, irrevocable, royalty-free basis to BMJ Publishing Group Ltd ("BMJ") its licensees and where the relevant Journal is co-owned by BMJ to the co-owners of the Journal, to publish the Work in this journal and any other BMJ products and to exploit all rights, as set out in our licence.

The Submitting Author accepts and understands that any supply made under these terms is made by BMJ to the Submitting Author unless you are acting as an employee on behalf of your employer or a postgraduate student of an affiliated institution which is paying any applicable article publishing charge ("APC") for Open Access articles. Where the Submitting Author wishes to make the Work available on an Open Access basis (and intends to pay the relevant APC), the terms of reuse of such Open Access shall be governed by a Creative Commons licence – details of these licences and which Creative Commons licence will apply to this Work are set out in our licence referred to above.

Other than as permitted in any relevant BMJ Author's Self Archiving Policies, I confirm this Work has not been accepted for publication elsewhere, is not being considered for publication elsewhere and does not duplicate material already published. I confirm all authors consent to publication of this Work and authorise the granting of this licence.

For peer review only

Associations of General and Central Adiposity with Hypertension and Cardiovascular Disease Among South Asian Populations: A Systematic Review and Meta-Analysis

Federica RE[1] MSc, Ayodipupo S. OGUNTADE[1] MSc, Bastian BOHRMANN[1] MSc, Fiona BRAGG[1,2] DPhil, Jennifer L. CARTER[1] PhD

[1]Clinical Trial Service Unit and Epidemiological Studies Unit (CTSU), Nuffield Department of Population Health, University of Oxford, Oxford, UK.

[2]MRC Population Health Research Unit, Nuffield Department of Population Health, University of Oxford, Oxford, UK.

Address for correspondence
Dr Jennifer Carter
Nuffield Department of Population Health
Big Data Institute
Roosevelt Drive, Oxford
OX3 7LF, UK
jennifer.carter@ndph.ox.ac.uk

Short title: Adiposity in hypertension and CVD risk among South Asians

Word count total: 3,745

Word count abstract: 295

Total tables in main text: 0

Total figures in main text: 5

For peer review only - http://bmjopen.bmj.com/site/about/guidelines.xhtml

ABSTRACT

Background: The relevance of measures of general and central adiposity for cardiovascular disease (CVD) risks in populations of European descent is well-established. However, it is less well-characterised in South Asian populations, who characteristically manifest larger waist circumferences (WC) for equivalent body mass index (BMI). This systematic review and meta-analysis provide an overview of the literature on the association of different anthropometric measures with CVD risk among South Asians.

Methodology: MEDLINE and Embase were searched from 1990 to present for studies in South Asian populations investigating associations of two or more adiposity measures with CVD. Random-effects meta-analyses were conducted on the associations of BMI, WC, and waist-to-hip ratio (WHR) with blood pressure, hypertension, and CVD. Quality assessment was performed using the Newcastle-Ottawa scale.

Results: Titles and abstracts were screened for 7,327 studies, yielding 147 full-text reviews. The final sample (n=30) included two prospective, five case-control, and 23 cross-sectional studies. Studies reported significant positive associations of adiposity measures with hypertension and CVD, but with limited evidence on shape. The pooled mean difference in systolic blood pressure (SBP) per 5kg/m^2 higher BMI was 3mmHg (2.90 [95%CI:1.30-4.50]), and 5mmHg (4.87; [95%CI:3.70-6.04]) per 10 cm larger WC. The odds ratio (OR) of hypertension per 5kg/m^2 higher BMI was 1.33 (95%CI:1.18-1.51), 1.33 (95%CI:1.04-1.69) per 10cm larger WC, and 1.22 (95%CI:1.04-1.41) per 0.1-unit larger WHR. Pooled risk of CVD for BMI-defined overweight versus healthy-weight was 1.65 (95%CI:1.55-1.75), and 1.48 (95%CI:1.21-1.80) and 2.51 (95%CI:0.94-6.69) for normal versus large WC and WHR, respectively. Study quality was average with significant heterogeneity.

Conclusions: Measures of both general and central adiposity had similar, strong positive associations with the risk of CVD in South Asians. Larger prospective studies

For peer review only

For peer review only - http://bmjopen.bmj.com/site/about/guidelines.xhtml

are required to clarify which measures of body composition are more informative for targeted CVD primary prevention in this population.

For peer review only

For peer review only - http://bmjopen.bmj.com/site/about/guidelines.xhtml

STRENGTHS AND LIMITATIONS OF THIS STUDY

- A broad literature search, robust duplicate and blinded screening, and a firm quality assessment represent major strengths of this study.
- Sensitivity analyses excluding large studies contributing the most to the models in the meta-analyses showed that associations remained largely unchanged.
- While the review aims to assess risk across South Asia, most studies were conducted in India, thus limiting generalisability of findings to the rest of the sub-continent.
- Cross-sectional design of studies included in the review increases the possibility of reverse causality.

For peer review only

For peer review only - http://bmjopen.bmj.com/site/about/guidelines.xhtml

INTRODUCTION

Being overweight or obese is one of the leading risk factors for premature mortality, estimated to account for up to 4.7 million deaths yearly.[1–3] The Global BMI Mortality Collaboration, a meta-analysis of 239 prospective studies, reported higher risk of cardiovascular disease (CVD) with higher body mass index (BMI) across all regions, showing that overweight individuals (BMI >25kg/m^2) experienced 49% higher risk per 5kg/m^2 higher BMI than individuals whose weight was within the healthy range (BMI 18.5-24.9kg/m^2).[4] Further meta-analyses across Europe, North America, and East Asia have reached similar conclusions.[5–7] The shape and strength of the association between BMI and CVD risk, however, has been found to differ across certain subgroups, with weaker associations among South Asians– though few studies have focused on these populations.[8,9]

A prospective cohort study of 0.5 million adults from the city of Chennai, India, found that blood pressure was strongly and positively associated with coronary heart disease (CHD) mortality (risk ratio [RR]: 1.70 [95%CI: 1.60-1.80] per 20mmHg higher usual SBP), but that BMI was little related to CHD mortality, despite increased BMI being a strong determinant of increased systolic blood pressure (SBP).[8] Moreover, the Asia Cohort Consortium observed positive associations between overweight BMI and CVD death, but the relation was substantially weaker and not statistically significant in South Asians compared to East Asians (South Asians: hazard ratio [HR] 1.03 [95%CI: 0.93-1.15] vs. East Asians: HR: 1.09 [95%CI: 1.03-1.15]).[10] The Global BMI Collaboration concluded similar findings, describing that, among South Asians, the risk of CVD per 5kg/m^2 higher BMI in overweight individuals was 10% and not statistically significant (HR: 1.10 [95%CI: 0.83-1.46]). This was considerably weaker than the associations observed in European populations (HR: 1.56 [95%CI: 1.54-1.58]).[4]

There is some evidence suggesting that South Asians are at higher risk of diabetes and CVD compared to Western populations.[11] However, the underlying pathophysiology leading to ethnic variations in the prevalence of hypertension (HTN) and the risk of CVD is poorly understood, although differences in body fat composition and distribution may

For peer review only - http://bmjopen.bmj.com/site/about/guidelines.xhtml

explain these discrepancies. As an anthropometric measure, BMI does not distinguish between central and peripheral adiposity, or between fat and lean mass. Some evidence suggests that centrally distributed visceral fat and ectopic fat are associated with cardiovascular outcomes, independently of BMI.[12,13] Furthermore, there are marked differences between central and general adiposity in their associations with fasting glucose, diabetes, and blood pressure, which lie on the causal pathway for CVD.[12,14] Therefore, research needs to assess whether measures of central adiposity are more important markers of disease risk in South Asian populations.

There is mixed evidence on whether higher BMI is associated with higher risk of CVD among South Asians and on which anthropometric measures are more strongly related with CVD risk in these populations. Hence, the purpose of this review is to provide an overview of the literature on the shape and strength of the association of BMI and CVD, including hypertension, among South Asians and to understand whether alternative anthropometric measures are better indicators of adiposity-related CVD risk in this geographical area compared to BMI. Results can be used to develop research which better characterizes the importance of adipose tissue and its distribution in CVD risk. Over time, this can help develop targeted preventative interventions and minimize health disparities.

METHODS

Search strategy

This systematic review followed the Cochrane Collaboration methods and adhered to the PRISMA reporting recommendations.[15] A predetermined review protocol was registered in the PROSPERO database (CRD42022308682). MEDLINE and Embase were searched combining terms relating to central and general adiposity and cardiovascular outcomes. Further terms were included to select for the geographical area of interest, namely South Asia (Afghanistan, Bangladesh, Bhutan, India, Maldives, Nepal, Pakistan, and Sri Lanka). The MeSH terms were chosen from the thesaurus used for indexing the subject headings. Full details of the search strategy for MEDLINE are shown in the Data Supplement **(Table S1)**. The syntax of the search strategy was

For peer review only - http://bmjopen.bmj.com/site/about/guidelines.xhtml

adapted to Embase. The search was limited to studies conducted on adults (18+years) and published in English between 1st January 1990 and 1st January 2023.

Eligibility criteria

Eligible studies included at least one measure of general (BMI, kg/m^2) and one measure of central adiposity (WC, WHR, waist-to-height ratio [WHtR], cm) as the exposure, and at least one cardiovascular outcome: SBP (mmHg), diastolic blood pressure (DBP, mmHg), hypertension (persistent blood pressure >140/90 mmHg), coronary artery disease (CAD), peripheral vascular disease (PVD) or CVD incidence/mortality.

The search included cross-sectional, case-control, and cohort studies. To limit potential chance findings arising from small studies, studies examining clinical endpoints (e.g., CVD, hypertension) were included in the review only if they included at least 50 events, while those examining continuous outcomes (e.g., blood pressure) were included if they included at least 100 participants. Studies that solely performed correlational analyses, those conducted among participants with prevalent diseases, or those on South Asian populations living outside of South Asia were excluded.

Study selection and data extraction

Studies were imported into Covidence, an online systematic review management platform, for abstract and full-text screening.[16] After removal of duplicates, titles and abstracts were reviewed independently by two reviewers (FR and ASO). Studies that did not meet the inclusion criteria were excluded. The full texts of the remaining papers were reviewed independently by the same reviewers. Disagreements were resolved by discussion and consensus between the two reviewers or, where necessary, by involving a third reviewer (JLC). A full list of included studies is reported in the Data Supplement (**Table S2** and **Table S3**). Associations were recorded for each study.

Quality assessment

Quality of included studies was assessed using validated cross-sectional, case-control, and cohort adaptations of the Newcastle-Ottawa scale.[17,18] Studies that fulfilled a

For peer review only - http://bmjopen.bmj.com/site/about/guidelines.xhtml

criterion were awarded a point for that criterion, while no point was awarded if the criterion was not fulfilled. Cohort and case-control studies could be awarded a maximum of nine points, while cross-sectional studies could be awarded a maximum of ten points. Studies were considered of high quality if they met at least seven out of nine criteria for cohort and case control studies, and eight out of ten criteria for cross-sectional studies. The quality of a study did not determine its inclusion in the systematic review or meta-analysis. Details of the quality assessment are available in the Data Supplement (**Table S4a/S4b/S4c**).

Data synthesis and analysis

For the dose-response meta-analyses, summary RR (95% CIs) per 5kg/m^2 higher BMI, 10cm higher WC, and 0.1-unit higher WHR were calculated using random effects models due to substantial heterogeneity of the included studies. These scales were chosen to aid comparison with other large-scale studies which have used the same units.[4,10] For comparison, additional fixed-effects models were also calculated. For each study, the risk estimate from the model including the greatest number of confounders, but not intermediate factors (e.g., diabetes, left ventricular hypertrophy, etc), was used. The average of the natural logarithm of the RRs was calculated.[19] In cases where studies provided RRs (95%CIs) per unit higher adiposity measure, these were scaled to the desired units by exponentiating the RR (95%CIs) to the power of desired units. When studies only reported RRs separately for different subgroups (e.g., age, sex, or ethnicity), these subgroup estimates were combined using a fixed-effects model to obtain an overall estimate. Each study was thus only represented once in each main meta-analysis.

Where studies reported estimates for categories of anthropometric measures, estimates were log-transformed and used to calculate study-specific slopes and 95% CIs across categories of anthropometric measures, as described by Greenland and Longnecker, to generate overall study-specific RRs.[20,21] Where studies only reported total cases and controls, total numbers were divided evenly across the categories.[22] The mean or median of each category of each anthropometric measure was assigned to the

For peer review only - http://bmjopen.bmj.com/site/about/guidelines.xhtml

corresponding RRs. For studies that did not report the mean or median of the anthropometric measures, the midpoint of the range of such categories was used as the mean. When the lowest or highest category was open-ended, the width of the interval was assumed to be the same as that of the adjacent category.[22] A likelihood ratio test was used to test nonlinearity by assessing the difference between the linear and non-linear models.

Heterogeneity between studies was determined using a Q-test, and I^2 statistics were used to denote the percentage of total variability due to between-study heterogeneity. I^2 >70% indicated high heterogeneity. To assess the robustness of the overall estimates, sensitivity analyses were undertaken removing one study at a time to determine whether results were influenced by large studies or studies with extreme results. Publication bias and small study effects were examined by inspecting funnel plots for asymmetry and with Egger's test. Analyses were conducted using Stata/MP 17.0 (StataCorp LLC, College Station, Texas, USA).

RESULTS

The initial search, after duplicate removal, included 7,327 studies. Of these, 30 were included in this review. **Figure 1** shows the number of papers excluded at each stage of the review process. At the screening stage, most exclusions were of studies looking at a single anthropometric measure only or those taking place in geographical regions not included in the review. On full-text assessment, most exclusions (n=85) were of studies performing only correlation analyses between anthropometric measures and CVD incidence. The number of participants in included studies varied between 140 and 59,037.[23,24] There were 25 (83.3%) included studies in India[23–47] and 3 (10%) in Bangladesh[48–50]. The remaining two studies were conducted in Mauritian[51] and Pakistani[52] populations, respectively. The final analyses included two prospective cohort studies (one looking at hypertension[25] and one looking at CVD mortality[51]), four case-control studies[44–47] (looking at CVD outcomes), and 24 cross-sectional studies[23,24,26–43,48–50,52] (looking at hypertension outcomes). In terms of exposure variables, 29 studies included BMI, 27 studies used WC, 21 studies used

For peer review only - http://bmjopen.bmj.com/site/about/guidelines.xhtml

WHR, and 10 studies additionally used other measures, including waist-to-height ratio (n=6) and hip circumference (n=2). Overall, 18 studies looked at hypertension[23,25,27–36,38,40,41,48–50], with or without blood pressure, eight looked at CVD[24,42–47,52], three looked at blood pressure alone[26,37,39], and one study looked at CVD mortality[51].

Associations between anthropometric indices and blood pressure

A total of four studies were included in the analysis of blood pressure (SBP/DBP). Of the four studies looking at the relationship between BMI and blood pressure, all studies concluded that higher BMI was related to higher blood pressure, with stronger associations with SBP than DBP. This was reflected in the meta-analysis, which showed that the pooled mean difference per 5kg/m^2 higher BMI was 3 mmHg (2.90 [95%CI: 1.30-4.50]) for SBP (**Figure 2**) and 2 mmHg (2.28 [95%CI: 0.55-4.01]) for DBP (**Data Supplement S5**). For WC, the pooled mean change in blood pressure per 10 cm larger WC was almost 5 mmHg (4.88 [95%CI: 3.72-6.04]) for SBP (**Figure 2**) and 4 mmHg (4.02 [95%CI: 2.45-5.59]) for DBP (**Data Supplement S6**). Associations were not statistically significant per 0.1-unit change in WHR (SBP: 2.27 [95%CI: -1.62-6.16]; DBP: 1.94 [95%CI: -1.42-5.29]). Findings of fixed-effects models are reported in the Data Supplement (**Figures S5 and S6**).

Associations between anthropometric indices and hypertension

All studies looking at hypertension risk reported positive associations between measures of general adiposity (BMI) and/or central adiposity (WC, WHR) with the risk of hypertension. Five cross-sectional studies concluded that the risk of hypertension was higher with a high BMI (≥25 kg/m^2) compared to a large WC (≥80cm in females, ≥90 in males) or WHR (≥0.8 in females, ≥1.0 in males). Eleven cross-sectional studies reported that measures of central adiposity, compared to BMI, showed stronger associations with hypertension. Of these, seven reported stronger associations with WC[28,32,35,41,48–50], and four with WHR[27,36,38,40]. The pooled odds ratio of hypertension per 5kg/m^2 higher BMI was 1.33 (95%CI: 1.18-1.51; **Figure 3A**). It was similar for a 10cm larger waist circumference (OR: 1.33 [95%CI: 1.04-1.69]; **Figure 3B**),

For peer review only - http://bmjopen.bmj.com/site/about/guidelines.xhtml

but weaker for a 0.1-unit larger WHR (OR: 1.22 [95%CI: 1.05-1.41]; **Figure 3C**), though all associations remained statistically significant. Heterogeneity (I^2) was >99% in all models. Fixed-effects models showed weaker associations (**Data Supplement S7**).

Association between anthropometric indices and CVD

The analyses of non-fatal and fatal CVD included one cohort, four cross-sectional and four case-control studies. Four studies concluded that there was a stronger association of high BMI than of high WC and WHR with risk of CVD and CVD mortality.[24,42,43,47] The remaining five studies concluded that measures of central adiposity, namely WC and/or WHR, showed stronger associations with CVD and CVD mortality than BMI.[44–46,51,52] The meta-analysis included 30,516 cases for the association of BMI with CVD, 31,274 cases for that of WC with CVD, and 30,537 for that of WHR with CVD. The pooled risk of CVD for overweight versus normal-weight individuals, as determined by BMI, was 1.65 (95%CI: 1.55-1.75; **Figure 4A**), despite three of the six studies making up this estimate concluding non-statistically significant findings.[43,45,46] Associations appeared weaker, but still statistically significant, for large versus normal WC (OR: 1.48 [95%CI: 1.21-1.80]; **Figure 4B**), and were not statistically significant for large versus normal WHR (OR: 2.51 [95%CI: 0.94-6.69]; **Figure 4C**). Heterogeneity (I^2) was >75% in all models. Associations of BMI with CVD and of WC with CVD appeared stronger in fixed-effects models, and statistically significant for WHR (OR: 1.50 [95%CI: 1.43-1.57]; **Data Supplement Figure S8**).

Sensitivity analyses

Sensitivity analyses were conducted removing one study at a time and determining whether results were influenced by large studies or studies with extreme results. The results were not substantively different (**Data Supplement S9**).

Study quality assessment and publication bias

Overall, the quality of the studies included in the review was average, with a mean score of 6.5/9 for cohort studies, 5.2/9 for case-control studies, and 7/10 for cross-sectional studies (**Data Supplement Tables S4a/S4b/S4c**). The domains in which

For peer review only - http://bmjopen.bmj.com/site/about/guidelines.xhtml

studies lost points, depending on design, were principally sample representativeness, control for additional confounding factors, sample size, and discussion of non-response rate.

Publication bias was assessed by inspecting funnel plots for asymmetry and using Egger's test. Funnel plots of studies reporting on risk of HTN associated with all three anthropometric indices (BMI, WC, and WHR) showed significant skew to the right of the panel (**Figure 5**). In all three plots, there were no studies in the lower left panel of the funnel, with most studies concentrating at the tip of the funnel and to the right of it. Funnel plots of studies reporting on the risk of CVD showed similar results (**Figure 5**).

DISCUSSION

The purpose of this review was to provide an overview of current literature examining the association of different anthropometric measures with CVD and hypertension among South Asian populations. While BMI appeared marginally more strongly associated with CVD, WC appeared to be more strongly associated with higher SBP and DBP. BMI and WC were similarly associated with HTN. Overall, there appears to be a limited amount of literature focusing on the shape of these associations, whereby studies only examined anthropometric measures as continuous variables (which assumes linearity) or as dichotomised variables, and an overall shape across the range of anthropometric measures was not assessed.

Comparison of South Asian and European effects

Compared to other literature, the present study found that associations between BMI and CVD in South Asians were comparable to those in European populations. The Global BMI Mortality Collaboration found that the risk of CVD per 5kg/m^2 increase in BMI was 10% (not statistically significant) for South Asians, compared to 56% in Europeans.[4] The Chennai Prospective Study later found that the risk of CVD for overweight vs. normal weight individuals in India was 28% (when adjusting for usual SBP).[8] However, associations presented herein showed a 65% increase in risk of CVD for overweight versus healthy weight individuals (OR: 1.65; [95%CI: 1.55-1.75]),

For peer review only

For peer review only - http://bmjopen.bmj.com/site/about/guidelines.xhtml

which is larger than the estimates attributed to South Asians in both the above studies and approximating the European risk reported by the Global BMI Mortality Collaboration. Interestingly, however, the majority of studies in this review examining associations between BMI and CVD reported non-significant findings.[45,52] A plausible reason for why associations have appeared weaker in South Asians compared to other populations is that South Asians may have protective correlates of high BMI that outweigh the adverse effects of elevated BMI on CVD.[53] In part, genetic differences may also affect body composition and metabolic risk and have thus contributed to disparities in effect.[54] However, non-significant findings may also be attributable to low study power, suggesting a need for larger studies directly comparing the use of various anthropometric measures to predict CVD among heterogenous South Asian populations.

With regards to blood pressure, the wider literature concluded that BMI was positively associated with SBP (about 1mmHg per 1kg/m^2), which is in concordance with the results of the present study where a slightly weaker mean difference of 2.90mmHg (95%CI: 1.30-4.50) in SBP per 5kg/m^2 higher BMI was found. However, the present study concluded that WC (OR: 4.88 [95%CI: 3.72-6.04]) showed stronger associations with blood pressure than both BMI and WHR. This is likely because South Asians, compared to populations of European descent, may manifest disproportionately larger waist circumferences for equivalent BMIs owing to a greater propensity to visceral adiposity. Further research has concluded that fat distribution, specifically central adipose tissue, may impact blood pressure variability over short-and long-term periods, with greater amounts of visceral fat linked to elevated but less variable blood pressure.[55] From an epidemiological standpoint, this could explain why different associations have been observed in different ethnicities. Several studies also concluded that South Asians appear to have higher lipid and insulin levels compared with Europeans of the same WC and WHR.[56–58] Asian Indians tend to have greater visceral and total body fat, which is less evident from BMI measurements and differs from the typical Western build.[59] In turn, increased visceral fat can cause insulin resistance, which may lead to metabolic disorders such as hypertension.

For peer review only - http://bmjopen.bmj.com/site/about/guidelines.xhtml

The INTERHEART case-control study, which compared populations, concluded that in all sub-groups, but particularly among South Asians and mixed-race Africans, WHR was a better predictor of CVD than BMI.[60] In the present study, however, WHR was generally weaker than BMI and WC in associations with SBP, CVD and hypertension, and across fixed-effects models. In INTERHEART, both WC and WHR were strongly associated with the risk of MI, but unlike BMI, this relationship was unaffected by mutual adjustment, suggesting there is a degree of independence between measures of adiposity in predicting the risk of myocardial infarction, stressing the relative importance central adiposity measures.[60] Thus, because SBP is strongly associated with CVD risk, some adverse or protective correlate of low BMI is likely associated with CVD, particularly among South Asians.[61,62]

Finally, the shape of the associations of different anthropometric indices and CVD among South Asian populations has been scarcely analysed across the literature, with most studies only examining anthropometric measures as dichotomous variables or by calculating risks for continuous measures that assume linearity. It was therefore not possible to review results across the range in comparison to the wider literature. Given the generally flat associations concluded by the Chennai Prospective Study, a comparison of shape across the range would make for a useful analysis.

Study quality assessment

The quality of the studies included in this review was average, with most studies scoring five or six out of nine or ten possible total points on the Newcastle-Ottawa scale. Several studies recorded CVD outcomes based on self-report or verbal autopsy, largely due to local lack of national registries or reported death certification. Eight studies investigating CVD endpoints such as stroke, myocardial infarction, or death from CVD were case-control or cross-sectional in design and therefore potentially limited by reverse causality. While the included studies are limited in some respects, the populations within each study were homogenous in terms of age, sex ratio, and education, indicating potential internal validity.

For peer review only - http://bmjopen.bmj.com/site/about/guidelines.xhtml

Strengths and limitations of this review

This meta-analysis has several strengths. The overall large size enables assessment of the relationship between different anthropometric measures and CVD, with an appreciation of all relevant literature on this topic. Despite significant heterogeneity, sensitivity analyses excluding large studies contributing the most to the models showed that associations remained largely unchanged.

Due to the nature of the studies and the extent of statistical heterogeneity observed, this systematic review also has limitations. Firstly, while the review aims to assess risk across South Asia, most studies were conducted in India, thus limiting generalisability of findings to the rest of the sub-continent. Nevertheless, this reflects the current body of evidence, and highlights an important gap in the literature. Secondly, it may have been beneficial to exclude cross-sectional and case-control studies in examining the association of anthropometric measures with CVD endpoints such as MI and CVD to minimize reverse causality. However, a large proportion of the identified evidence was based on these study designs and excluding such studies would have skewed findings, thus biasing results. Thirdly, despite ensuring the most fully adjusted models were used for the meta-analyses, we cannot rule out the possibility that observed associations are confounded by unmeasured factors such as physical activity and diet, including dietary salt consumption. Few studies adjusted for physical activity– a known confounder of cardiovascular health. The majority of studies also failed to control for other important confounders, for example menopause status among females or dietary salt consumption, which are related independently to both anthropometric indices and CVD.[25,51]

CONCLUSION

From a clinical point of view, health practitioners should be made aware of ethnic variations in CVD risk and how these relate to different measures of anthropometry. There is scope for measures such as WC and WHR to become routinely included in health records, alongside BMI, if these are truly deemed stronger CVD predictors. However, these measures would need to be robustly measured, which is not always

For peer review only - http://bmjopen.bmj.com/site/about/guidelines.xhtml

straightforward in busy clinical environments and one of the reasons why BMI is more widely employed. Development of a point-of-care CVD risk score based on these measures may also prove an effective population-level prevention strategy.

Ultimately, large prospective studies among South Asian populations are required to clarify whether measures of central adiposity may be better predictors of CVD. Ideally, these studies would directly compare different measures of adiposity with risk over time. There is also potential value in imaging-based studies to characterize the distribution of adipose tissue more reliably. More considered cut-offs of different body composition measures, which consider location of fat deposition, may be needed, as well as an assessment of the shape of the relationship across the full range. Given the high prevalence of CVD globally, and the rapidly increasing prevalence among South Asian populations, this may have important implications from a public health perspective with potential to achieve better-targeted CVD primary prevention.

For peer review only - http://bmjopen.bmj.com/site/about/guidelines.xhtml
For peer review only

FIGURE LEGENDS

Figure 1. PRISMA flowchart describing the systematic literature search and study selection.

Figure 2. Mean change in systolic blood pressure (SBP) per 5kg/m² higher body mass index (BMI, A), 10cm larger waist circumference (WC, B) and 0.1-unit larger waist-to-hip ratio (WHR, C). Random effects models were applied to four studies reporting associations of SBP and BMI, four studies reporting on SBP and WC, and two studies reporting on SBP and WHR. The total number of participants was 8,143 for panels A and B, and 7,781 for panel C. *Abbreviations: 95% confidence interval (95%CI).*

Figure 3. Odds ratio (OR) of hypertension (HTN) per 5kg/m² higher body mass index (BMI, A), 10cm larger waist circumference (WC, B) and 0.1-units larger waist-to-hip ratio (WHR, C). Random effects models were applied to 13 studies looking at BMI, seven studies looking at WC, and seven studies looking at WHR. The total number of participants was 11,616 for panel A, 4,899 for panel B, and 4,456 for panel C. *Abbreviations: 95% confidence interval (95%CI).*

Figure 4. Odds ratios (OR) of cardiovascular disease (CVD) for overweight (>25kg/m²) vs. normal (18.5-24.9 kg/m²) body mass index (BMI, panel A), large ((≥80cm in females, ≥90cm in males) vs. normal waist circumference (WC, panel B), and large (≥0.8-units in females, ≥1.0-units in males) vs. normal waist-to-hip ratio (WHR, panel C). Random effects models were applied to six studies looking at BMI, seven studies looking at WC, and six studies looking at WHR. *Abbreviations: 95% confidence interval (95%CI).*

Figure 5. Funnel plots of studies on BMI-HTN (A), WC-HTN (B), WHR-HTN (C) and BMI-CVD (D), WC-CVD (E) and WHR-CVD (F). Egger's test was b=2.27 (p=0.0025) for BMI and HTN, b=2.96 (p<0.001) for WC and HTN, and b=4.58 (p=0.003) for WHR and HTN. With regards to the CVD measures, Egger's test was b=0.43 (p=0.659) for BMI and CVD, b=0.42 (p=0.614) for WC and CVD and b=4.32 (p=0.0031) for WHR and CVD. *Abbreviations: body mass index, kg/m² (BMI); hypertension (HTN); waist circumference, cm (WC); waist-to-hip ratio (WHR); odds ratio (OR); 95% confidence interval (95%CI).*

For peer review only - http://bmjopen.bmj.com/site/about/guidelines.xhtml

Acknowledgements: None.

Funding: The study was funded by core support from the UK Medical Research Council (MRC), British Heart Foundation, and Cancer Research UK to the Clinical Trial Service Unit and the MRC Population Health Research Unit, both now in the Nuffield Department of Population Health, University of Oxford (Oxford, UK).

Competing interests: None declared.

Patient and public involvement: Patients and/or the public were not involved in the design, conduct, reporting, or dissemination plans of this research.

Patient consent for publication: Not applicable.

Data availability: Individual data should be requested from the original or parent study investigators of the studies included in this review.

Ethical approval: Ethical approval was not required for the present systematic review and meta-analysis. It is assumed that all published research on which analyses were based will have fulfilled respective ethical standards at the time of publication.

For peer review only

For peer review only - http://bmjopen.bmj.com/site/about/guidelines.xhtml

REFERENCES

1 Hubert HB, Mcnamara PM, Castelli WP. Obesity as an independent risk factor for cardiovascular disease: a 26-year follow-up of participants in the Framingham Heart Study. *Circulation* 1983;**67**:10. doi:10.1161/01.cir.67.5.968

2 Manson J, Colditz G, Stamper M, *et al.* A Prospective Study of Obesity and Risk of Coronary Heart Disease in Women. *The New England Journal of Medicine* 1990;**322**:882–9. doi:10.1056/NEJM199003293221303

3 Dorn J, Schisterman E, Winkelstein W, *et al.* Body Mass Index and Mortality in a General Population Sample of Men and Women: The Buffalo Health Study. *Am J Epidemiol* 1997;**128**:919–31. doi:10.1093/oxfordjournals.aje.a009218

4 Di Angelantonio E, Bhupathiraju SN, Wormser D, *et al.* Body-mass index and all-cause mortality: individual-participant-data meta-analysis of 239 prospective studies in four continents. *The Lancet* 2016;**388**:776–86. doi:10.1016/S0140-6736(16)30175-1

5 Fan J, Song Y, Chen Y, *et al.* Combined effect of obesity and cardio-metabolic abnormality on the risk of cardiovascular disease: A meta-analysis of prospective cohort studies. *International Journal of Cardiology* 2013;**168**:4761–8. doi:10.1016/j.ijcard.2013.07.230

6 McGee DL, The Diverse Population Collaboration. Body mass index and mortality: a meta-analysis based on person-level data from twenty-six observational studies. *Annals of Epidemiology* 2005;**15**:87–97. doi:10.1016/j.annepidem.2004.05.012

7 van Dijk SB, Takken T, Prinsen EC, *et al.* Different anthropometric adiposity measures and their association with cardiovascular disease risk factors: a meta-analysis. *Neth Heart J* 2012;**20**:208–18. doi:10.1007/s12471-011-0237-7

8 Gajalakshmi V, Lacey B, Kanimozhi V, *et al.* Body-mass index, blood pressure, and cause-specific mortality in India: a prospective cohort study of 500 810 adults. *Lancet Glob Health* 2018;**6**:e787–94. doi:10.1016/S2214-109X(18)30267-5

9 Yusuf S, Hawken S, Ôunpuu S, *et al.* Obesity and the risk of myocardial infarction in 27 000 participants from 52 countries: a case-control study. *The Lancet* 2005;**366**:1640–9. doi:10.1016/S0140-6736(05)67663-5

10 Chen Y, Copeland WK, Vedanthan R, *et al.* Association between body mass index and cardiovascular disease mortality in east Asians and south Asians: pooled analysis of prospective data from the Asia Cohort Consortium. *BMJ* 2013;**347**. doi:10.1136/bmj.f5446

11 Shah A, Kanaya AM. Diabetes and Associated Complications in the South Asian Population. *Curr Cardiol Rep* 2014;**16**:476. doi:10.1007/s11886-014-0476-5

For peer review only - http://bmjopen.bmj.com/site/about/guidelines.xhtml

12 Blüher M, Laufs U. New concepts for body shape-related cardiovascular risk: role of fat distribution and adipose tissue function. *European Heart Journal* 2019;**40**:2856–8. doi:10.1093/eurheartj/ehz411

13 Neeland IJ, Poirier P, Després J-P. Cardiovascular and Metabolic Heterogeneity of Obesity: Clinical Challenges and Implications for Management. *Circulation* 2018;**137**:1391–406. doi:10.1161/CIRCULATIONAHA.117.029617

14 Huxley R, Mendis S, Zheleznyakov E, *et al.* Body mass index, waist circumference and waist:hip ratio as predictors of cardiovascular risk—a review of the literature. *Eur J Clin Nutr* 2010;**64**:16–22. doi:10.1038/ejcn.2009.68

15 Page M, McKenzie J, Bossuyt P, *et al.* The PRISMA 2020 statement: an updated guideline for reporting systematic reviews. *BMJ* Published Online First: 2021. doi:10.1136/bmj.n71

16 Cochrane Community. Covidence. The Future of Evidence Synthesis in Cochrane. 2022.

17 Wells G, Shea B, O'Connell D, *et al.* The Newcastle-Ottawa Scale (NOS) for Assessing the Quality of Nonrandomised Studies in Meta-analyses. Ottawa Hospital Research Institute. https://www.ohri.ca/programs/clinical_epidemiology/oxford.asp

18 Modesti P, Reboldi G, Cappuccio F, *et al.* Panethnic differences in blood pressure in Europe: a systematic review and meta-analysis. *PLoS ONE* 2016;**11**. doi:10.1371/journal.pone.0147601

19 Green J, Cairns B, Casabonne D, *et al.* Height and cancer incidence in the Million Women Study: prospective cohort, and meta-analysis of prospective studies of height and total cancer risk. *The Lancet Oncology* 2011;**12**:785–94. doi:10.1016/S1470-2045(11)70154-1

20 Orsini N. Weighted mixed-effects dose–response models for tables of correlated contrasts. *The Stata Journal* 2021;**21**:320–47. doi:10.1177/1536867X211025798

21 Shim S, Shin I, Yoon B, *et al.* Dose-response meta-analysis using STATA Software. *J Health Info Stat* 2016;**41**:351–8. doi:10.21032/jhis.2016.41.3.351

22 Papier K, Knuppel A, Syam N, *et al.* Meat consumption and risk of ischemic heart disease : A systematic review and meta-analysis. *Critical Reviews in Food Science and Nutrition* 2021;**0**:1–12. doi:10.1080/10408398.2021.1949575

23 Deshpande-Joshi S, Rao S. Differential risk of hypertension among lean and non-lean rural subjects in relation to decadal changes in anthropometry. *J Am Coll Nutr* 2018;**37**:380–6. doi:10.1080/07315724.2017.1405750

For peer review only - http://bmjopen.bmj.com/site/about/guidelines.xhtml

24 Bramhankar M, Pandey M, Rana G, *et al.* An assessment of anthropometric indices and its association with NCDs among the older adults of India: evidence from LASI Wave-1. *BMC Public Health* 2021;**21**:1357. doi:10.1186/s12889-021-11421-4

25 Agrawal P, Gupta K, Aboyans V, *et al.* Women's health in India: the role of body mass index. *Health Care Women Int* 2014;**36**:320–41. doi:10.1080/07399332.2014.892110

26 Bose K, Ghosh A, Roy S, *et al.* The relationship of age, body mass index and waist circumference with blood pressure in Bengalee Hindu male jute mill workers of Belur, West Bengal, India. *Anthropology Anz* 2005;**63**:205–12. doi:PMID: 15962571

27 Dhall M, Devi K, Nilupher A, *et al.* Hypertension and its correlate with general and central adiposity: A study among urban population of Delhi. *Diabetes Metab Syndr* 2018;**12**:881–4. doi:10.1016/j.dsx.2018.05.008

28 Ganguli D, Das N, Saha I, *et al.* Risk factors for hypertension in a population-based sample of postmenopausal women in Kolkata, West Bengal, India. *Asia Pac J Public Health* 2013;**25**:388–97. doi:10.1177/1010539511420703

29 Ghosh J, Bandyopadhyay A. Comparative evaluation of obesity measures: relationship with blood pressure and hypertension. *Singapore Med J* 2007;**48**:232–5. doi:PMID: 17342293

30 Hazarika N, Narain K, Biswas D, *et al.* Hypertension in the native rural population of Assam. *Natl Med J India* 2004;**17**:300–4. doi:PMID: 15736549

31 Kaur P, Rao S, Radhakrishnan E, *et al.* Prevalence, awareness, treatment, control and risk factors for hypertension in a rural population in South India. *Int J Public Health* 2012;**57**:87–94. doi:10.1007/s00038-011-0303-3

32 Kaur P, Radhakrishnan E, Sankarasubbaiyan S, *et al.* A Comparison of Anthropometric Indices for Predicting Hypertension and Type 2 Siabetes in a Male Industrial Population of Chennai, South India. *Ethnicity and Disease* 2008;**18**:31–26.

33 Meshram II, Arlappa N, Balkrishna N, *et al.* Prevalence of hypertension, its correlates and awareness among adult tribal population of Kerala state, India. *J Postgrad Med* 2012;**58**:255–61. doi:10.4103/0022-3859.105444

34 Meshram II, Vishnu Vardhana Rao M, Sudershan Rao V, *et al.* Regional variation in the prevalence of overweight/obesity, hypertension and diabetes and their correlates among the adult rural population in India. *Br J Nutr* 2016;**115**:1265–72. doi:10.1017/S0007114516000039

35 Shriraam V, Mahadevan S, Arumugam P. Prevalence and risk factors of diabetes, hypertension and other non-communicable diseases in a tribal population in South India. *Indian J Endocr Metab* 2021;**25**:313. doi:10.4103/ijem.ijem_298_21

For peer review only - http://bmjopen.bmj.com/site/about/guidelines.xhtml

36 Singh R, Mukherjee M, Kumar R, *et al.* Study of risk factors of coronary heart disease in urban slums of Patna. *Nepal J Epidemiology* 2012;**2**:205–12. doi:10.3126/nje.v2i3.6902

37 Taing K, Farkouh M, Moineddin R, *et al.* Age and sex-specific associations of anthropometric measures of adiposity with blood pressure and hypertension in India: a cross-sectional study. *BMC Cardiovasc Disord* 2016;**16**. doi:10.1186/s12872-016-0424-y

38 Tselha N, Shimrah C, Kulshreshtha M, *et al.* Association between hypertension and adiposity indicators: A study among the Muslim population of Uttar Pradesh. *Diabetes Metab Syndr* 2019;**13**:2335–8. doi:10.1016/j.dsx.2019.05.016

39 Venkatramana P, Reddy P. Association of overall and abdominal obesity with coronary heart disease risk factors: comparison between urban and rural Indian men. *Asia Pac J Clin Nutr* 2002;**11**:66–71. doi:10.1046/j.1440-6047.2002.00250.x

40 Vikram N, Latifi A, Misra A, *et al.* Waist-to-height ratio compared to standard obesity measures as predictor of cardiometabolic risk factors in Asian Indians in North India. *Metab Syndr Relat Disord* 2016;**14**:492–9. doi:10.1089/met.2016.0041

41 Yadav S, Boddula R, Genitta G, *et al.* Prevalence & risk factors of pre-hypertension & hypertension in an affluent north Indian population. *Indian J Med Res* 2008;:712–20. doi:PMID: 19246794

42 Banerjee S, Kumar P, Srivastava S, *et al.* Association of anthropometric measures of obesity and physical Activity with ardiovascular diseases among older adults: evidence from a cross-sectional survey, 2017–18. *PLoS ONE* 2021;**16**. doi:10.1371/journal.pone.0260148

43 Bodkhe S, Jajoo S, Jajoo U, *et al.* Epidemiology of confirmed coronary heart disease among population older than 60 years in rural central India—A community-based cross-sectional study. *Indian Heart Journal* 2019;**71**:39–44. doi:10.1016/j.ihj.2019.01.002

44 Latheef SAA, Subramanyam G, Reddy BM. Utility of anthropometric traits and indices in predicting the risk of coronary artery disease in the adult men of southern Andhra Pradesh. *Indian Heart J* 2018;**70**:S133–9. doi:10.1016/j.ihj.2018.07.016

45 Patil S, Joshi R, Gupta G, *et al.* Risk factors for acute myocardial infarction in a rural population of central India: A hospital-based case–control study. *Nat Med J India* 2004;**17**. doi:PMID: 15372760

46 Prabhakar S, Suravarapu S, Mathai D, *et al.* Risk factors for stroke in rural population of Telangana State of India, an unmatched case-control study. *J Neurosci Rural Pract* 2020;**11**:448–53. doi:10.1055/s-0040-1713291

For peer review only

For peer review only - http://bmjopen.bmj.com/site/about/guidelines.xhtml

47 Shah M, Mazumdar V, Patel S, *et al.* A case control study of risk factors of coronary heart disease among patients admitted at tertiary hospital in western India. *Australasian Medical Journal* 2017;**10**:381–8. doi:10.21767/AMJ.2017.2860

48 Islam FMA, Bhuiyan A, Chakrabarti R, *et al.* Undiagnosed hypertension in a rural district in Bangladesh: The Bangladesh Population-based Diabetes and Eye Study (BPDES). *J Hum Hypertens* 2016;**30**:252–9. doi:10.1038/jhh.2015.65

49 Khanam R, Ahmed S, Rahman S, *et al.* Prevalence and factors associated with hypertension among adults in rural Sylhet district of Bangladesh: a cross-sectional study. *BMJ Open* 2019;**9**. doi:10.1136/bmjopen-2018-026722

50 Simmons S, Hagan Jr. J, Schack T. The influence of anthropometric indices and intermediary determinants of hypertension in Bangladesh. *IJERPH* 2021;**18**. doi:10.3390/ijerph18115646

51 Cameron A, Magliano D, Shaw J, *et al.* The influence of hip circumference on the relationship between abdominal obesity and mortality. *Intl J of Epi* 2012;**41**:484–94. doi:10.1093/ije/dyr198

52 Nishtar S, Wierzbicki A, Lumb P, *et al.* Waist-hip ratio and low HDL predict the risk of coronary artery disease in Pakistanis. *Current Medical Research and Opinion* 2008;**20**:55–62. doi:10.1185/030079903125002595

53 Skogen J, Øverland S. The fetal origins of adult disease: a narrative review of the epidemiological literature. *JRSM Short Reports* 2012;**3**:1–7. doi:10.1258/shorts.2012.012048

54 Li X, Qi L. Gene–Environment Interactions on Body Fat Distribution. *IJMS* 2019;**20**:3690. doi:10.3390/ijms20153690

55 Levelt E, Pavlides M, Banerjee R, *et al.* Ectopic and Visceral Fat Deposition in Lean and Obese Patients With Type 2 Diabetes. *J Am Coll Cardiol* 2016;**68**:53–63. doi:10.1016/j.jacc.2016.03.597

56 Chandalia M, Abate N, Garg A, *et al.* Relationship between Generalized and Upper Body Obesity to Insulin Resistance in Asian Indian Men. *The Journal of Clincial Endocrineology and Metabolism* 1999;**84**:7.

57 Lean M, Han T, Bush H, *et al.* Ethnic differences in anthropometric and lifestyle measures related to coronary heart disease risk between South Asian, Italian and general-population British women living in the west of Scotland. *Int J Obes Relat Metab Disord* 2001;**25**:1800–5. doi:10.1038/sj.ijo.0801823

58 Rush E, Goedecke J, Jennings C, *et al.* BMI, fat and muscle differences in urban women of five ethnicities from two countries. *Int J Obes* 2007;**31**:1232–9. doi:10.1038/sj.ijo.0803576

For peer review only - http://bmjopen.bmj.com/site/about/guidelines.xhtml

59 Banerji MA, Faridi N, Atluri R, *et al.* Body Composition, Visceral Fat, Leptin, and Insulin Resistance in Asian Indian Men. *Journal of Clinical Endocrineology and Metabolism* 1999;**84**:8. doi:10.1210/jcem.84.1.5371

60 Yusuf S, Hawken S, McQueen M, *et al.* Effect of potentially modifiable risk factors associated with myocardial infarction in 52 countries (the INTERHEART study): case-control study. *The Lancet* 2004;**364**:937–52. doi:10.1016/S0140-6736(04)17018-9

61 Deurenberg P, Deurenberg-Yap M, Guricci S. Asians are different from Caucasians and from each other in their body mass index/body fat per cent relationship. *Obesity Reviews* 2002;**3**:141–6. doi:10.1046/j.1467-789X.2002.00065.x

62 Lear SA, Humphries KH, Kohli S, *et al.* The Use of BMI and Waist Circumference as Surrogates of Body Fat Differs by Ethnicity**. *Obesity* 2007;**15**:2817–24. doi:10.1038/oby.2007.334

For peer review only

For peer review only - http://bmjopen.bmj.com/site/about/guidelines.xhtml

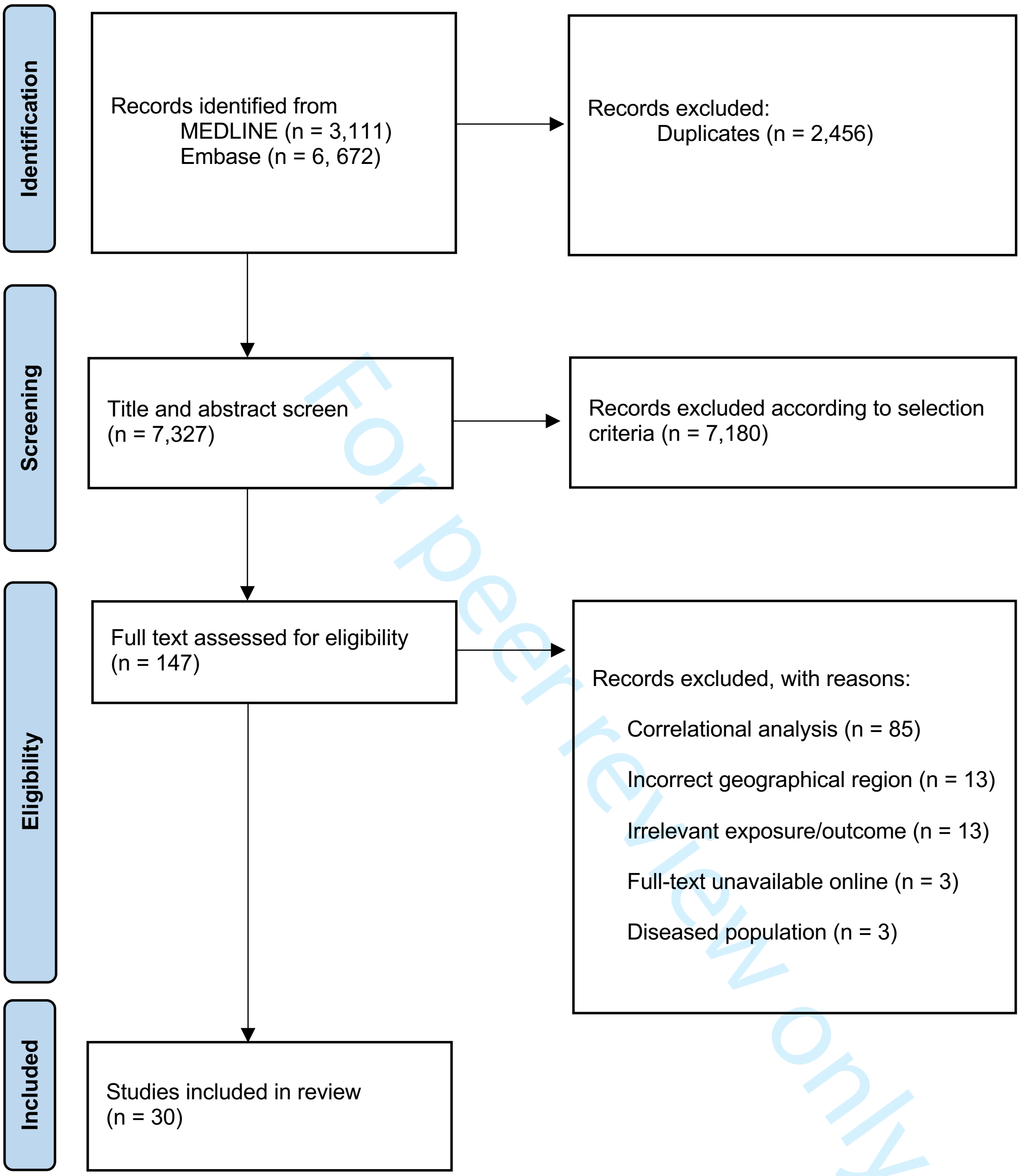

For peer review only

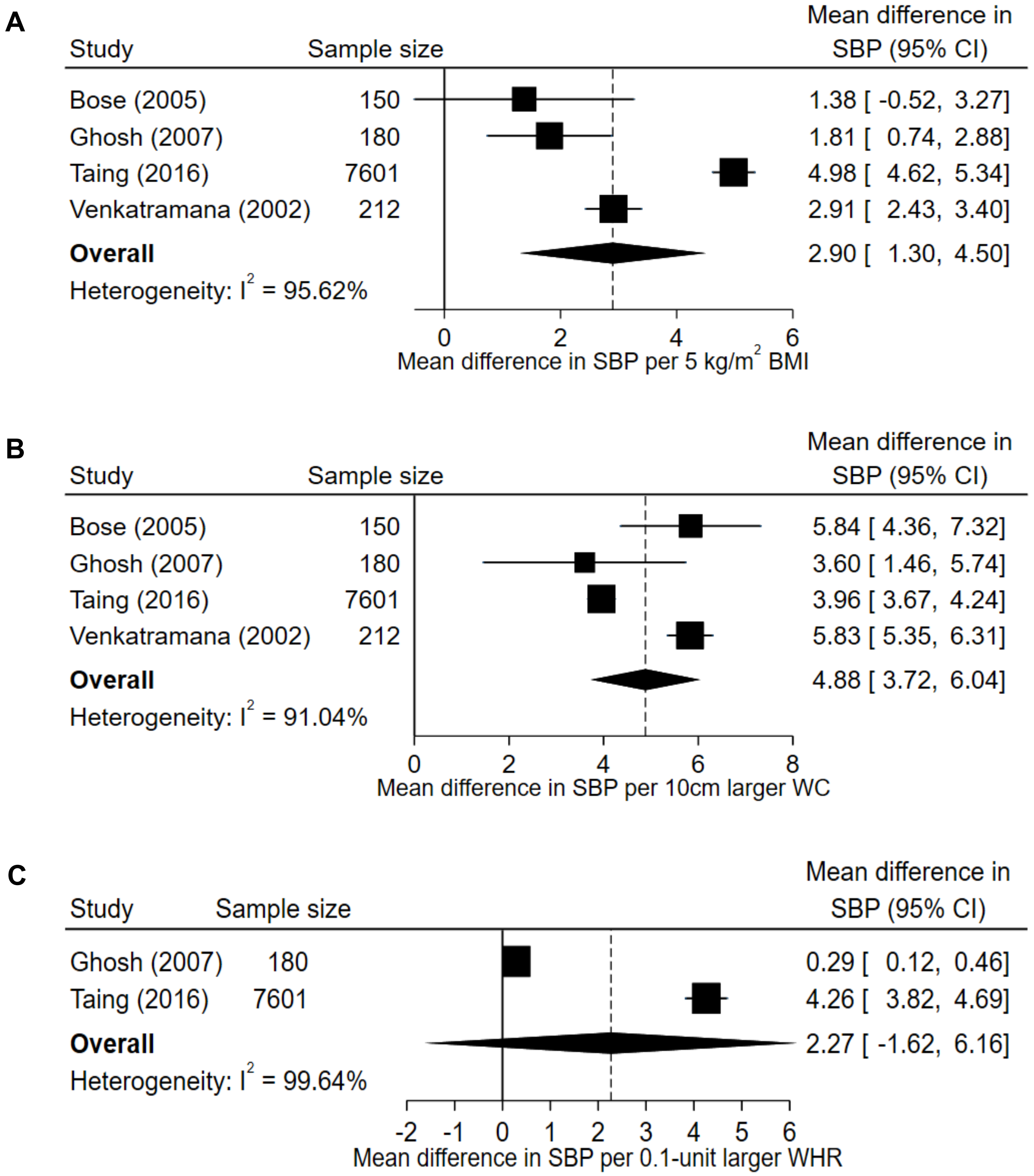
A
Study
Sample size
Mean difference in SBP (95% CI)
Bose (2005) 150 1.38 [-0.52, 3.27]
Ghosh (2007) 180 1.81 [0.74, 2.88]
Taing (2016) 7601 4.98 [4.62, 5.34]
Venkatramana (2002) 212 2.91 [2.43, 3.40]
Overall 2.90 [1.30, 4.50]
Heterogeneity: I² = 95.62%
0 2 4 6
Mean difference in SBP per 5 kg/m² BMI
B
Study
Sample size
Mean difference in SBP (95% CI)
Bose (2005) 150 5.84 [4.36, 7.32]
Ghosh (2007) 180 3.60 [1.46, 5.74]
Taing (2016) 7601 3.96 [3.67, 4.24]
Venkatramana (2002) 212 5.83 [5.35, 6.31]
Overall 4.88 [3.72, 6.04]
Heterogeneity: I² = 91.04%
0 2 4 6 8
Mean difference in SBP per 10cm larger WC
C
Study
Sample size
Mean difference in SBP (95% CI)
Ghosh (2007) 180 0.29 [0.12, 0.46]
Taing (2016) 7601 4.26 [3.82, 4.69]
Overall 2.27 [-1.62, 6.16]
Heterogeneity: I² = 99.64%
-2 -1 0 1 2 3 4 5 6
Mean difference in SBP per 0.1-unit larger WHR

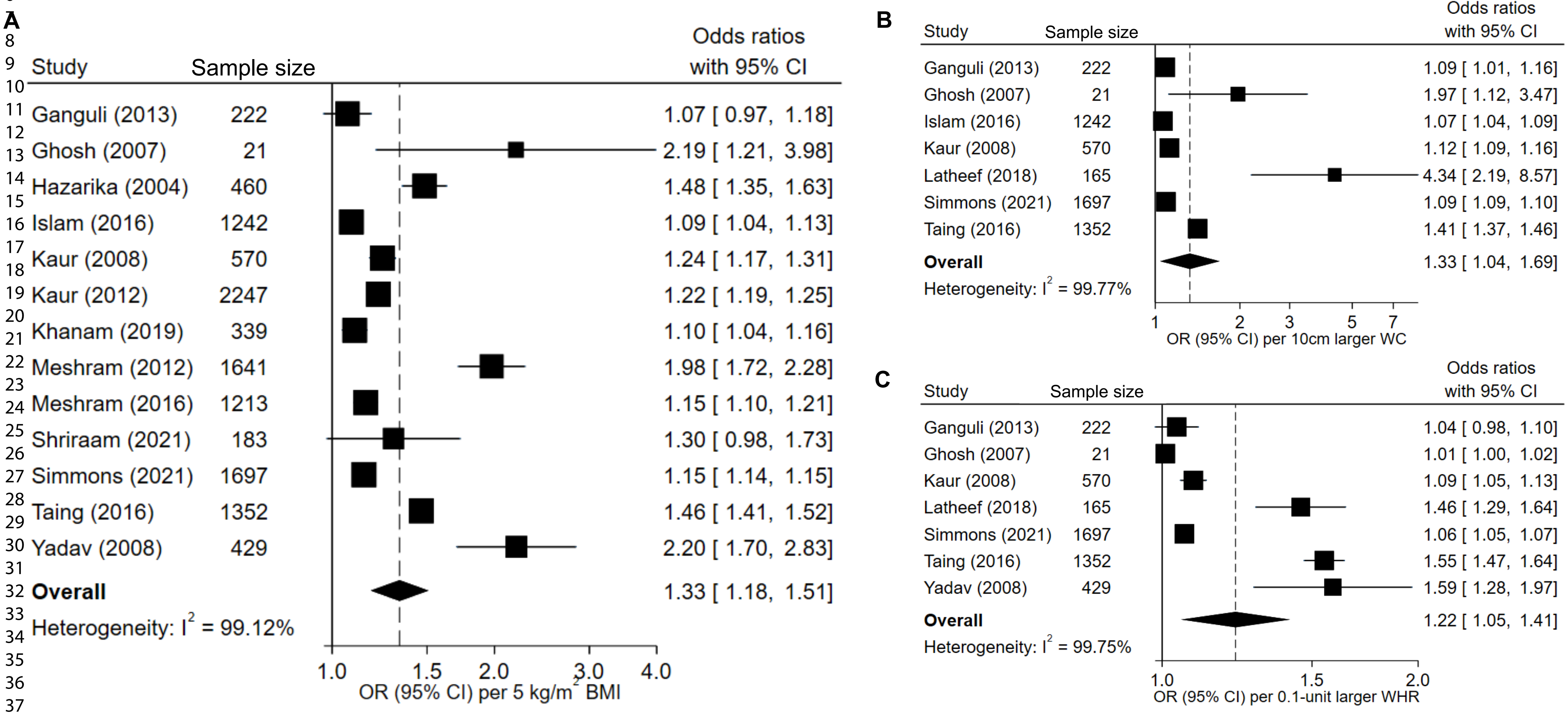
A
Study
Sample size
Odds ratios
with 95% CI
Ganguli (2013) 222 1.07 [0.97, 1.18]
Ghosh (2007) 21 2.19 [1.21, 3.98]
Hazarika (2004) 460 1.48 [1.35, 1.63]
Islam (2016) 1242 1.09 [1.04, 1.13]
Kaur (2008) 570 1.24 [1.17, 1.31]
Kaur (2012) 2247 1.22 [1.19, 1.25]
Khanam (2019) 339 1.10 [1.04, 1.16]
Meshram (2012) 1641 1.98 [1.72, 2.28]
Meshram (2016) 1213 1.15 [1.10, 1.21]
Shriraam (2021) 183 1.30 [0.98, 1.73]
Simmons (2021) 1697 1.15 [1.14, 1.15]
Taing (2016) 1352 1.46 [1.41, 1.52]
Yadav (2008) 429 2.20 [1.70, 2.83]
Overall 1.33 [1.18, 1.51]
Heterogeneity: I2 = 99.12%
1.0 1.5 2.0 3.0 4.0
OR (95% CI) per 5 kg/m2 BMI
B
Study
Sample size
Odds ratios
with 95% CI
Ganguli (2013) 222 1.09 [1.01, 1.16]
Ghosh (2007) 21 1.97 [1.12, 3.47]
Islam (2016) 1242 1.07 [1.04, 1.09]
Kaur (2008) 570 1.12 [1.09, 1.16]
Latheef (2018) 165 4.34 [2.19, 8.57]
Simmons (2021) 1697 1.09 [1.09, 1.10]
Taing (2016) 1352 1.41 [1.37, 1.46]
Overall 1.33 [1.04, 1.69]
Heterogeneity: I2 = 99.77%
1 2 3 5 7
OR (95% CI) per 10cm larger WC
C
Study
Sample size
Odds ratios
with 95% CI
Ganguli (2013) 222 1.04 [0.98, 1.10]
Ghosh (2007) 21 1.01 [1.00, 1.02]
Kaur (2008) 570 1.09 [1.05, 1.13]
Latheef (2018) 165 1.46 [1.29, 1.64]
Simmons (2021) 1697 1.06 [1.05, 1.07]
Taing (2016) 1352 1.55 [1.47, 1.64]
Yadav (2008) 429 1.59 [1.28, 1.97]
Overall 1.22 [1.05, 1.41]
Heterogeneity: I2 = 99.75%
1.0 1.5 2.0
OR (95% CI) per 0.1-unit larger WHR

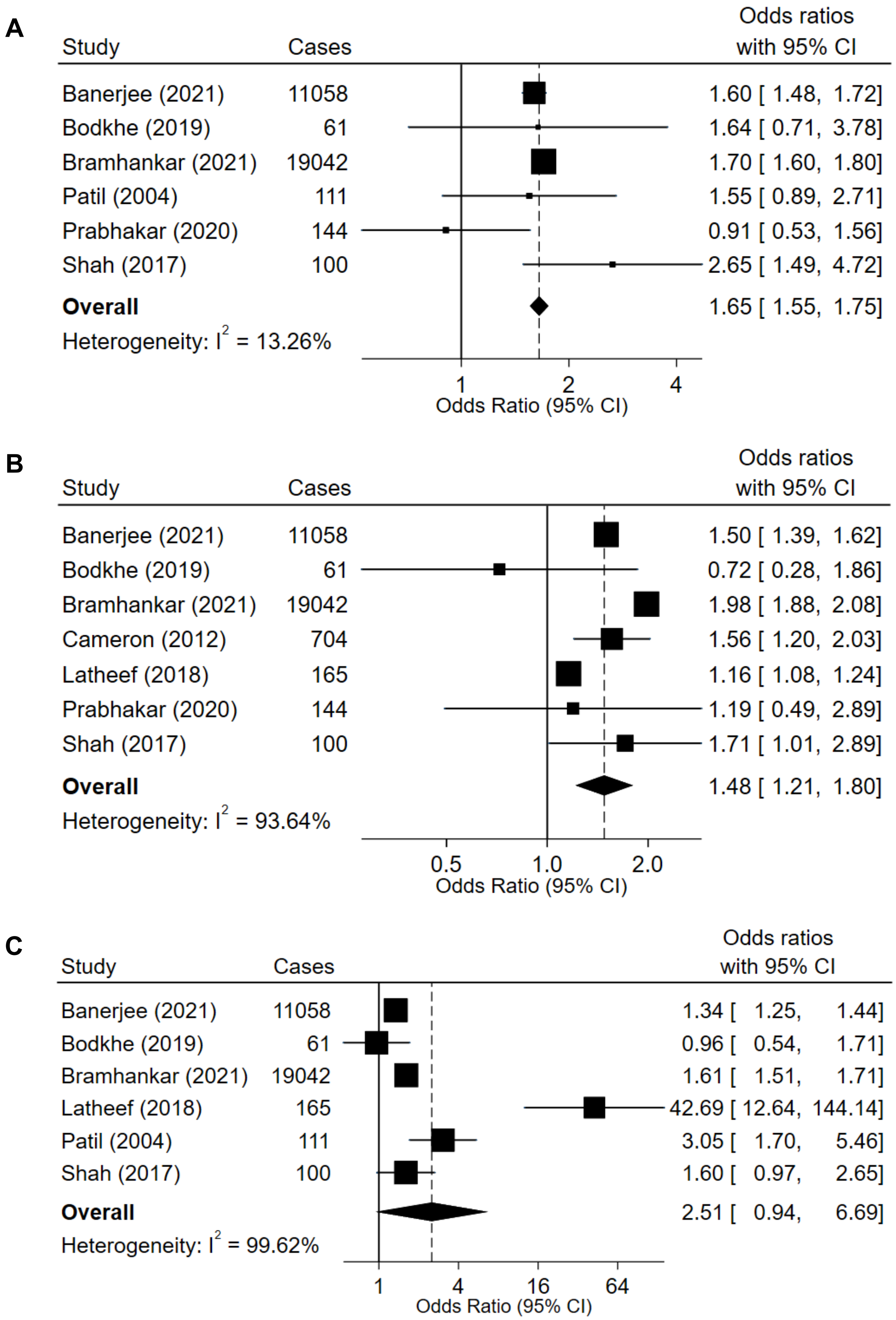
A
Study
Cases
Odds ratios with 95% CI
Banerjee (2021) 11058 1.60 [1.48, 1.72]
Bodkhe (2019) 61 1.64 [0.71, 3.78]
Bramhankar (2021) 19042 1.70 [1.60, 1.80]
Patil (2004) 111 1.55 [0.89, 2.71]
Prabhakar (2020) 144 0.91 [0.53, 1.56]
Shah (2017) 100 2.65 [1.49, 4.72]
Overall 1.65 [1.55, 1.75]
Heterogeneity: I^2 = 13.26%
1
2
4
Odds Ratio (95% CI)
B
Study
Cases
Odds ratios with 95% CI
Banerjee (2021) 11058 1.50 [1.39, 1.62]
Bodkhe (2019) 61 0.72 [0.28, 1.86]
Bramhankar (2021) 19042 1.98 [1.88, 2.08]
Cameron (2012) 704 1.56 [1.20, 2.03]
Latheef (2018) 165 1.16 [1.08, 1.24]
Prabhakar (2020) 144 1.19 [0.49, 2.89]
Shah (2017) 100 1.71 [1.01, 2.89]
Overall 1.48 [1.21, 1.80]
Heterogeneity: I^2 = 93.64%
0.5
1.0
2.0
Odds Ratio (95% CI)
C
Study
Cases
Odds ratios with 95% CI
Banerjee (2021) 11058 1.34 [1.25, 1.44]
Bodkhe (2019) 61 0.96 [0.54, 1.71]
Bramhankar (2021) 19042 1.61 [1.51, 1.71]
Latheef (2018) 165 42.69 [12.64, 144.14]
Patil (2004) 111 3.05 [1.70, 5.46]
Shah (2017) 100 1.60 [0.97, 2.65]
Overall 2.51 [0.94, 6.69]
Heterogeneity: I^2 = 99.62%
1
4
16
64
Odds Ratio (95% CI)

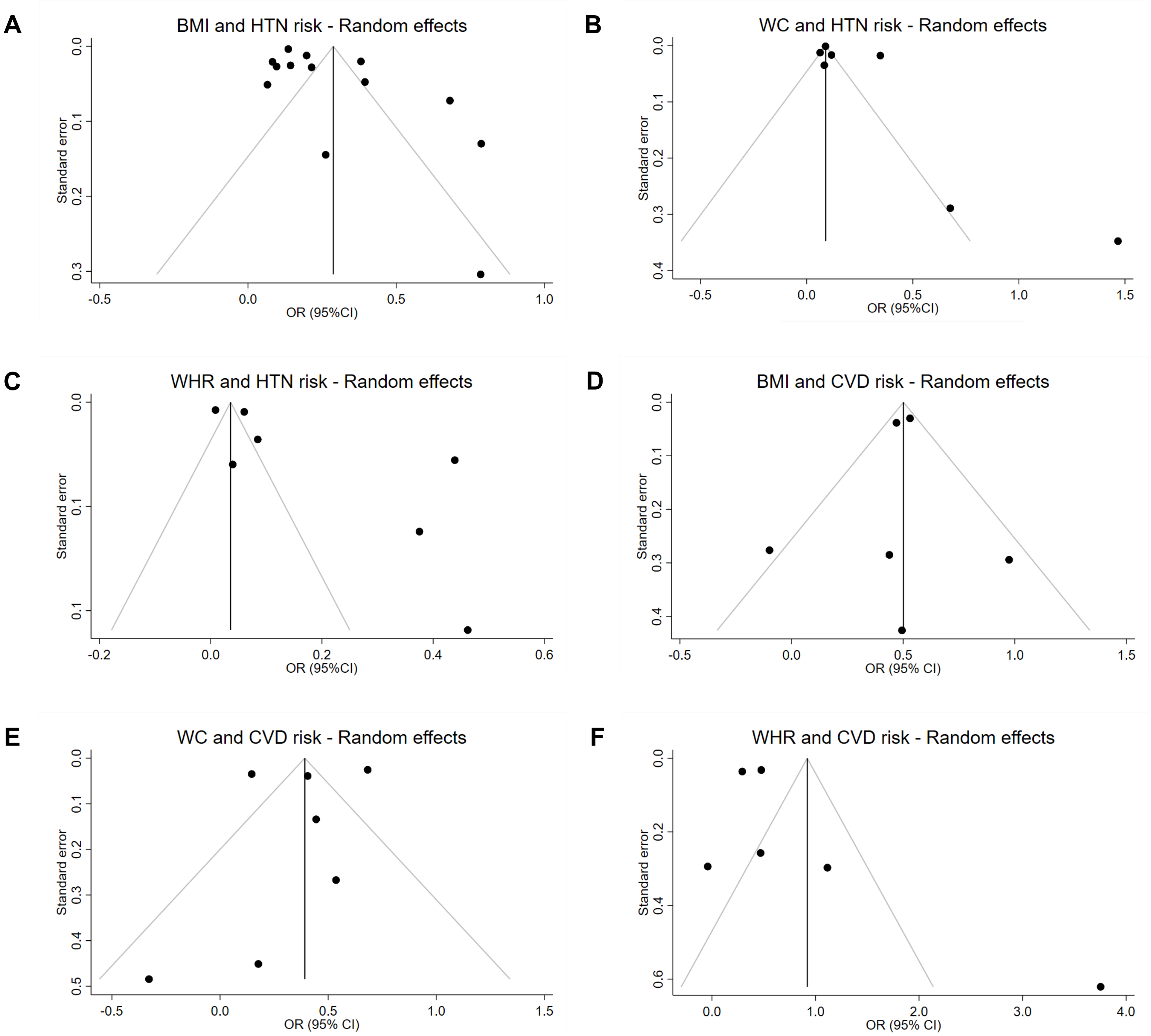
A
BMI and HTN risk - Random effects
Standard error
OR (95%CI)
B
WC and HTN risk - Random effects
Standard error
OR (95%CI)
C
WHR and HTN risk - Random effects
Standard error
OR (95%CI)
D
BMI and CVD risk - Random effects
Standard error
OR (95% CI)
E
WC and CVD risk - Random effects
Standard error
OR (95% CI)
F
WHR and CVD risk - Random effects
Standard error
OR (95% CI)

DATA SUPPLEMENT

Associations of General and Central Adiposity with Hypertension and Cardiovascular Disease Among South Asian Populations: A Systematic Review and Meta-Analysis

Federica RE[1] MSc, Ayodipupo S. OGUNTADE[1] MSc, Bastian BOHRMANN[1] MSc, Fiona BRAGG[1,2] DPhil, Jennifer L. CARTER[1] PhD

1. Clinical Trial Service Unit and Epidemiological Studies Unit (CTSU), Nuffield Department of Population Medicine, University of Oxford, Oxford, UK.

2. MRC Population Health Research Unit, Nuffield Department of Population Health, University of Oxford, Oxford, UK

Address for correspondence
Dr Jennifer Carter
Nuffield Department of Population Health
Big Data Institute
Roosevelt Drive, Oxford
OX3 7LF, UK
jennifer.carter@ndph.ox.ac.uk

For peer review only

For peer review only - http://bmjopen.bmj.com/site/about/guidelines.xhtml

CONTENTS

Supplemental Table S1. Search strategy in MEDLINE……………………………………3

Supplemental Table S2. Summary of studies included in literature review looking at the association of measures of adiposity with blood pressure (BP) and hypertension (HTN)………………………………………………………………………………………4

Supplemental Table S3. Summary of studies included in literature review looking at the association of measures of adiposity with cardiovascular disease (CVD) risk and mortality……………………………………………………………………………………10

Supplemental Tables S4a/S4b/S4c. Quality assessment of included studies…………13

Supplemental Figure S5. Mean change in systolic blood pressure (SBP, panel B) and diastolic blood pressure (DBP, panels A and C) per 5kg/m^2 higher body mass index (BMI)………………………………………………………………………………...………..15

Supplemental Figure S6. Mean change in systolic blood pressure (SBP, panel B) and diastolic blood pressure (DBP, panels A and C) per 10cm larger waist circumference (WC)………………………………………………………………………………………..16

Supplemental Figure S7. Odds ratio (OR) of hypertension per 5kg/m^2 higher body mass index (BMI, A), 10cm larger waist circumference (WC, B) and 0.1-units larger waist-to-hip ratio (WHR, C)………………………………………………………………….……17

Supplemental Figure S8. Odds ratio of cardiovascular disease for overweight vs. normal body mass index (BMI, panel A), large vs. normal waist circumference (WC, panel B), and large vs. normal waist-to-hip ratio (WHR, panel C)………………………………...18

Supplemental results S9. Additional details on sensitivity analyses results…………….19

Data supplement reference list…………………………………………………………….20

For peer review only

For peer review only - http://bmjopen.bmj.com/site/about/guidelines.xhtml

Table S1. Search strategy in MEDLINE

Keyword	Thesaurus (MeSH)	Textword
Exposures	Anthropometry/ or body composition/ or body fat distribution/ or body weight/ or overweight/ or thinness/ or Adipose Tissue/ or Adiposity/ or body fat distribution/ or body mass index/ or body size/ or waist-hip ratio/ or Obesity/ or abdominal fat/ or intra-abdominal fat/ or subcutaneous fat, abdominal/ or subcutaneous fat/ or Waist Circumference/ or Waist-Height Ratio/	(abdom* or intraabdom* or central or truncal or trunk or visceral or android or gynoid) adj fat?).mp<br>(central adiposity or obes* or fat distribution or waist circumference or hip circumference or waist-hip ratio or waist-to-hip ratio or waist-height ratio or waist-to-height ratio or waist-to-stature ratio or WHR or WHtR or electric impedance or bio-impedance or bio-electric impedance or sagittal abdominal diameter).mp<br>(anthropometr* or adipos* or body weight or body mass index or quetelet index or BMI or fat mass* or body fat* or fatness or lean mass or fat-free mass or overweight or body composition or body size or skeletal mass or muscle mass).mp
Outcomes	exp Coronary Disease/ or exp Myocardial Ischemia/ or Myocardial Infarction/ or exp Peripheral Vascular Diseases/ or exp Cerebrovascular Disorders/ or Cardiovascular Diseases/ or exp Hypertension/ or Blood Pressure/	(coronary heart disease or coronary artery disease or angina* or myocardial infarction or coronary isch?emia or myocardial isch?emia or heart attack* or isch?emic heart disease or CHD or stroke* or cerebrovascular accident or cerebrovascular disorder or cerebrovascular disease or peripheral arter* disease or peripheral vascular disease or cardiovascular disease or CVD or cardiovascular risk or vascular risk or vascular disease or hypertension or HTN or blood pressure).mp.
Ethnicity	Afghanistan/ or Bangladesh/ or Bhutan/ or India/ or Maldives/ or Nepal/ or Pakistan/ or Sri Lanka/	(South Asia* or Afghan* or Bangladesh*or Bhutan* or India* or Maldiv* or Nepal* or Pakistan* or Sri Lanka*).mp.
Study type	exp case-control studies/ or exp cohort studies/ or exp cross-sectional studies/ or clinical trial/ or observational study/ or Randomized Controlled Trials as Topic/	(cohort* or longitudinal* or prospective* or follow-up* or observational * or inciden* or case-control or retrospective or cross-sectional or population-based or randomi?ed controlled trial* or randomi?ed trial* or clinical trial* or association* or risk*).mp.

For peer review only - http://bmjopen.bmj.com/site/about/guidelines.xhtml

Table S2. Summary of studies included in literature review looking at the association of measures of adiposity with blood pressure (BP) and hypertension (HTN)

Results report associations in terms of mean change (mmHg) in BP or odds ratios (OR) of HTN. Additionally, 95% confidence intervals (95%CI) are reported where available. Unless otherwise specified, cut-offs used throughout for different measures of adiposity are: underweight BMI=<18.5kg/m^2, normal BMI=18.6-25.9kg/m^2, overweight BMI=26-29.9kg/m^2, obese BMI=≥30kg/m^2, low-risk/normal WC females=<80cm, high-risk/large WC females=≥80cm, low-risk/normal WC males=<94cm, high-risk/large WC males=≥94cm, low-risk/normal WHR females=<0.85cm, high-risk/large WHR females=≥0.85cm, low-risk/normal WHR males=<0.90cm, high-risk/large WHR males=≥90cm.

Abbreviations: BMI= body mass index (kg/m^2); WHR=waist-to-hip ratio (cm); WC=waist circumference (cm); SBP=systolic blood pressure (mmHg); DBP=diastolic blood pressure (mmHg); WHtR=waist-to-height ratio (cm); B=beta coefficient; t=two-tailed t-test.

Author (study design)	Geographical location	No. of participants (% of women)	Mean (SD) age, years	Adiposity measurements	Mean follow-up, years	Outcomes	Variables adjusted for	Results
Agrawal et al., 2014[1] (prospective cohort)	India	325 (100)	38 (N/A)	BMI, WHR	4	HTN	Age-group, education, religion, caste, employment status, standard of living	The likelihood of HTN was higher among obese women (OR: 3.86; p<0.0001) compared to women with a normal BMI. The likelihood of HTN among women with large WHR was 2.5 times higher (OR=2.47; p=0.002).
Bose et al., 2005[2] (cross-sectional)	India	150 (0)	40.7 (15.2)	BMI, WC	N/A	SBP, DBP, mean arterial pressure	Age	Regression of BMI and WC with SBP (B= 0.275, t=1.426), DBP (B= 0.009, t=0.067) and mean arterial pressure (B=0.098, t=0.065) showed that BMI did not have a significant impact on SBP, DBP, or mean arterial pressure. WC had a significant impact on SBP (B=0.506, t= 7.068), DBP (B=0.393, t=5.190) and mean arterial pressure (B=0.461, t=6.387).
Deshpande-Joshi et al., 2017[3] (cross-sectional)	India	140 (0)	32.4 (2)	BMI, WC, WHR	N/A	SBP, DBP, HTN	Not provided	Odds of HTN were highest for subjects in the highest tertile of BMI (OR: 18.9; 95% CI: 4.1–87.3) followed by large WC (OR: 11.4; 95% CI:1.6–13.0), and large WHR (OR: 6.6; 95% CI: 2.2–19.7).

For peer review only - http://bmjopen.bmj.com/site/about/guidelines.xhtml

Author (study design)	Geographical location	No. of participants (% of women)	Mean (SD) age, years	Adiposity measurements	Mean follow-up, years	Outcomes	Variables adjusted for	Results
Dhall et al., 2018[4] (cross-sectional)	India	568 (56.0)	32 (median age)	BMI, WC, WHR	N/A	SBP, DBP, HTN	Not provided	For males, large WC was significantly associated with risk of HTN (OR: 6.76; 95% CI: 2.57-17.76). Odds of HTN were found to be highest among those with risk category of WHR and conicity index in females. However, no significant association was observed.
Ganguli et al., 2013[5] (cross-sectional)	India	415 (100)	54.5 (9.4)	BMI, WC, WHR	N/A	HTN	Age, total cholesterol, exercise frequency, family history of HTN	WC was significantly associated with HTN (OR: 2.55; 95%CI: 1.07-6.06). WHR was significantly associated with HTN in the unadjusted (OR: 2.26, 95%CI: 1.39-3.68) and age-adjusted models (OR: 2.17, 95%CI: 1.31-3.59). BMI was not significantly associated with HTN in the unadjusted, age-adjusted, or multivariable-adjusted models.
Ghosh et al., 2007[6] (cross-sectional)	India	180 (N/A)	45.7 (9.3)	BMI, WC, WHR, WHtR	N/A	SBP, DBP, HTN	Age	Large WC was significantly associated with HTN (OR: 1.07; 95%CI: 1.0-1.12), along with overweight BMI (OR: 1.17; 95%CI: 1.04-1.32), large WHR (OR: 1.09; 95%CI: 1.01-1.17), and large WHtR (OR: 1.12; 95%CI: 1.03-1.22).
Hazarika et al., 2004[7] (cross-sectional)	India	3180 (54.7)	Partici-pants aged >30	BMI, WHR	N/A	HTN	Age, sex, marital status, type of work, alcohol, smoking, tobacco chewing	Overweight and BMI were significantly associated with HTN (OR overweight: 1.95; 95%CI: 1.37-2.78 and OR obese: 3.10; 95%CI: 1.17-8.22). Large WHR was also associated with HTN (OR: 1.54; 95%CI: 1.25-1.90).

For peer review only - http://bmjopen.bmj.com/site/about/guidelines.xhtml

Author (study design)	Geographical location	No. of participants (% of women)	Mean (SD) age, years	Adiposity measurements	Mean follow-up, years	Outcomes	Variables adjusted for	Results
Islam et al., 2016[8] (cross-sectional)	Bangladesh	3104 (65.0)	Not reported	BMI, WC	N/A	HTN	Age, education, diabetes	Among both males and females, BMI was significantly associated with mild, moderate, and severe hypertension. The same pattern followed for quartiles of WC, though associations appeared stronger than for BMI *(see full-text, Table 3 for details)*.
Kaur et al., 2008[9] (cross-sectional)	India	2148 (0)	40.5 (11.6)	BMI, WC, WHR, WHtR		SBP, DBP, HTN	Age	All four anthropometric indices showed a significant unadjusted OR for HTN across the quintiles. BMI and WC showed significant age-adjusted OR in all quintiles. WC showed a significant increase in age and BMI adjusted OR in the last two quintiles (*see full-text for quintiles)*.
Kaur et al., 2012[10] (cross-sectional)	India	10463 (53.2)	Not reported	BMI, WC		HTN	Age	Odds of HTN were higher in the general obesity category (BMI ≥27.50kg/m^2) compared to the highest central obesity category (WC ≥80cm), OR BMI: 3.25; 95%CI: 2.57-4.11 and OR WC: 2.51; 95%CI: 2.12-2.98.
Khanam et al., 2019[11] (cross-sectional)	Bangladesh	1810 (52.3)	Male median: 50 (IQR 42–60) Female median: 47 (IQR 40–57)	BMI, WC		HTN	Age, education, wealth status, smoking, fruit and vegetables (BMI models adjustments unavailable)	Compared to healthy weight, overweight BMI was significantly associated with greater risk of HTN in both males (OR: 2.9; 95%CI: 1.8-4.6) and females (OR: 1.6; 95%CI: 1.1-2.4). Odds of HTN were fourfold higher among males (OR: 4.0; 95%CI: 2.5-6.4) and threefold higher among females (OR: 2.8; 95%CI: 2.0-4.1) with large WC.
Meshram et al., 2012[12] (cross-sectional)	India	4193 (54.9)	43.3 (14.9) for males and 42.4 (14.4) for females	BMI, WC	N/A	HTN	Age, sex, education, wealth index, alcohol	Large WC and overweight BMI were associated with two-fold (OR for abdominal obesity=1.7; 95%CI=1.24-2.33, OR for

For peer review only - http://bmjopen.bmj.com/site/about/guidelines.xhtml

Author (study design)	Geographical location	No. of participants (% of women)	Mean (SD) age, years	Adiposity measurements	Mean follow-up, years	Outcomes	Variables adjusted for	Results
								BMI=1.7; 95%CI=1.35-2.21) increase in risk of HTN.
Meshram et al., 2016[13] (cross-sectional)	India	8969 (49.5)	38.2 (15.4)	BMI, WC, WHR	N/A	HTN	Age, sex, education, occupation	Overweight individuals had 2.1 times higher risk of HTN (2.17; 95%CI 1.68-2.81), while individuals with large WC had two times higher risk (OR 1.96; CI 1.61-2.42). Individuals with large WHR had 1.4 times higher risk of HTN (OR 1.35; 95%CI 1.13-1.62).
Shriraam et al., 2021[14] (cross-sectional)	India	502 (57.8)	55.1 (10.86)	BMI, WC	N/A	HTN	Age, sex, occupation, physical activity, diabetes	Being overweight was not significantly associated with increased risk of HTN (OR: 1.27; 95%CI: 0.76-2.13). However, being obese was (OR: 9.75; 95%CI: 2.06-46.19). WC was significantly associated with increased risk of HTN (OR:1.62; 95%CI: 1.07-2.48).
Simmons et al., 2021[15] (cross-sectional)	Bangladesh	8019 (53)	See cate-gories in full-text	BMI, WC, WHR, WHtR	N/A	HTN	Age, residence, education, fruits and vegetables, alcohol, physical activity	Risk of HTN was higher among females and males who were overweight (OR: 1.35; 95%CI: 1.23-1.62 and OR: 2.40; 95%CI: 2.34-2.62) and obese (OR: 1.68; 95%CI: 1.44-1.89 and OR: 2.62; 95%CI: 2.02-2.94). A moderate WC, defined as 94-102cm in males and 80-88cm in females, was associated with higher risk of HTN among both groups (OR females: 1.72; 95%CI: 1.64-1.91; OR males: 2.14; 95%CI: 2.05-2.28). A stronger pattern was observed for large WC (OR: 2.52; 95%CI: 2.05-2.98 and OR: 2.65; 95%CI: 2.34-2.85). Patterns were similar for WHR and WHtR.

For peer review only - http://bmjopen.bmj.com/site/about/guidelines.xhtml

Author (study design)	Geographical location	No. of participants (% of women)	Mean (SD) age, years	Adiposity measurements	Mean follow-up, years	Outcomes	Variables adjusted for	Results
Singh et al., 2012[16] (cross-sectional)	India	3118 (36.5)	Not reported	BMI, WHR	N/A	HTN	Tobacco, alcohol	BMI was significantly associated with increased risk of HTN (OR: 1.52; 95%CI: 1.25-1.85), as was WHR (OR: 1.65; 95%CI: 1.36-2.00).
Taing et al., 2016[17] (cross-sectional)	India	7601 (50.1)	40 (11)	BMI, WC, HC, WHR, WHtR	N/A	SBP, DBP, mean arterial pressure	Education, location, WC, BMI	Every 5 kg/m^2 greater BMI or 10cm wider WC was associated with a 5 and 4mmHg higher SBP, and a 4 and 3mmHg higher DBP. The association between WC and DBP was stronger than the that of BMI and DBP.
Tselha et al., 2019[18] (cross-sectional)	India	214 (53.2)	34.0 (16.0) in males and 33.7 (11.4) in females	BMI, WC, WHR, WHtR	N/A	HTN	Not provided	In males, WHR followed by WC and BMI were found to be associated with HTN: OR: 1.48 (96%CI:-0.59-3.72); 1.16 (95%CI:-0.37-3.65); and 1.11 (95%CI:0.11-11.20). In females, WHtR, BMI and WHR were found to be the strongest predictors of HTN: OR: 6.82 (95%CI: -2.68-17.39); 1.31 (95%CI: - 0.33-5.30); and 1.06 (95%CI: 0.21-3.17), respectively.
Venkatramana et al., 2002[19] (cross-sectional)	India	212 (0)	Urban: 47.4 (9.1); rural: 40.8 (14.2)	BMI, WC	N/A	SBP, DBP	Age, smoking, physical activity	BMI did not have significant effects on SBP (B=0.050, p=0.891) and DBP (B=0.225, p=0.544) in the urban population. However, it did have significant effects in the rural population (SBP B=1.182, p<0.001; DBP B=1.047, p=0.005). WC did not have significant effects on SBP (B=0.224, p=0.660) or DBP (B=0.140, p=0.788) in the urban population or the rural population (SBP: B=-0.990, p=0.059; DBP: B=0.140, p=0.788).

For peer review only - http://bmjopen.bmj.com/site/about/guidelines.xhtml

Author (study design)	Geographical location	No. of participants (% of women)	Mean (SD) age, years	Adiposity measurements	Mean follow-up, years	Outcomes	Variables adjusted for	Results
Vikram et al., 2016[20] (cross-sectional)	India	509 (45.3)	40.0 (8.6) in males and 38.7 (9.2) in females	BMI, WC, WHR, WHtR	N/A	HTN	Crude	In males, being overweight (OR: 2.12; 95%CI 1.07-4.37), having a large WC (OR: 2.65; 95%CI: 1.33-5.26), WHR (OR: 2.27; 95%CI: 0.91-5.61), and WHtR (OR: 3.85; 95%CI: 1.57-9.43) was associated with HTN. In females, overweight BMI (OR: 4.00; 95%CI: 1.16-13.80) and large WC (OR: 5.25; 95%CI: 1.52-18.11) were significantly associated with HTN. Large WHR (OR: 4.21; 95%CI: 0.96-18.47) was not statistically significant.
Yadav et al., 2008[21] (cross-sectional)	India	1746 (31.8)	49.8 (11.5)	BMI, WC, WHR	N/A	HTN	Age, sex	Being overweight was associated with HTN (OR: 2.2; 95%CI: 1.5-3.1). Associations were stronger for large WC (OR: 4.1; 95%CI: 2.6-6.4), but weaker for large WHR (OR: 1.56; 95%CI: 1.37-1.79).

For peer review only - http://bmjopen.bmj.com/site/about/guidelines.xhtml

Table S3. Summary of studies included in literature review looking at the association of measures of adiposity with cardiovascular disease (CVD) risk and mortality

Results report associations in terms hazard ratios (HR), or relative risk (RR). Additionally, 95% confidence intervals (95%CI) and p-values (p) are reported where available. Unless otherwise specified, cut-offs used throughout for different measures of adiposity are: underweight BMI=<18.5kg/m^2, normal BMI=18.6-25.9kg/m^2, overweight BMI=26-29.9kg/m^2, obese BMI=≥30kg/m^2, low-risk/normal WC females=<80cm, high-risk/large WC females=≥80cm, low-risk/normal WC males=<94cm, high-risk/large WC males=≥94cm, low-risk/normal WHR females=<0.85cm, high-risk/large WHR females=≥0.85cm, low-risk/normal WHR males=<0.90cm, high-risk/large WHR males=≥90cm.

Abbreviations: BMI=body mass index (kg/m^2); WC=waist circumference (cm); WHR=waist-to-hip ratio (cm); CHD=coronary heart/artery disease; HC=hip circumference.

Author (study design)	Geographical location	No. of participants (% of women)	Mean (SD) age, years	Adiposity measurements	Mean follow-up, years	Outcomes	Adjustments	Results
Banerjee et al., 2021[22] (cross-sectional)	India	31464 (N/A)	N/A, participants >60	BMI, WC, WHR	N/A	CVD	Age, sex, education, marital status, working status, tobacco, alcohol, physical activity, monthly per-capital consumption expenditure, religion, caste, place of residence, region	Likelihood of CVD was higher among adults who were overweight (OR: 1.60; CI: 1.48–1.72), had a large WC (OR: 1.50; CI: 1.39–1.62) and high-risk WHR (AOR: 1.34; CI: 1.25–1.44), compared to adults with normal BMI and those who do not have a high-risk WC and high risk WHR.
Bodkhe et al., 2019[23] (cross-sectional)	India	1190 (51)	N/A, participants >60	BMI, WC, WHR	N/A	CHD	Age, sex, education, socioeconomic status, physical activity, HTN, diabetes, tobacco	No significant associations between risk of CHD and obesity (OR for BMI: 1.64; 95%CI: 0.71-3.77; OR for WC: 0.72; 95%CI: 0.28-1.87; OR for WHR: 0.96; 95%CI: 0.54-1.71).
Bramhankar et al., 2021[24] (cross-sectional)	India	37536 (N/A)	N/A, participants >45	BMI, WC, WHR	N/A	CVD	Age, sex, education, employment, residence, caste, wealth quintile, region, smoking, alcohol	Adults were 2.3 times more likely (OR: 2.33; 95%CI: 2.2-2.5) by obesity, 61% more likely (OR: 1.61; 95%CI: 1.63-1.63) by high-risk WHR and 98% more likely (OR: 1.98; 95%CI: 1.9-2.1) by high-risk WC to develop CVD than their normal-weight individuals.

For peer review only - http://bmjopen.bmj.com/site/about/guidelines.xhtml

Author (study design)	Geographical location	No. of participants (% of women)	Mean (SD) age, years	Adiposity measurements	Mean follow-up, years	Outcomes	Adjustments	Results
Cameron et al., 2012[25] (prospective cohort)	Mauritius (South Asian Mauritius and Africa Mauritius reported separately)	7,979	Not reported	BMI, WC, HC	15.1	Death from CVD	Age, sex, smoking	In the South Asian population, high-risk WC was significantly related to CVD death in males (OR: 1.8; 95%CI: 1.2-2.7) and females (OR: 1.5; 95%CI: 1.1-2.2), compared with those below the obesity cut-off, when adjusting for hip circumference.
Latheef et al., 2018[26] (case-control)	India	165 (0)	Not reported	WC, WHR	N/A	CAD	Not provided	Association of anthropometric variables with CAD:<br>WC- OR: 1.16; 95%CI: 1.08-1.24. WHR- OR: 42.69; 95%CI: 10.28-177.20.
Nishtar et al., 2008[27] (cross-sectional)	Pakistan	400 (16)	Cases: 51.2 (9.5)<br>Controls: 48.2 (9.5)	BMI, WC, WHR	N/A	CAD	Age, urban/rural, family history of CAD, smoking, diabetes, HTN, BP	A significant association of WC (OR: 1.02; 95%CI: 1.00-1.04) and WHR (OR: 1.06; 95%CI: 1.03-1.10) was found with CAD. The association was not significant for BMI (OR: 1.02; 95%CI: 0.96-1.08).
Patil et al., 2004[28] (case-control)	India	Cases 111 (20.7)<br>Controls 222 (20.7)	*See categories in full-text*	BMI, WHR	N/A	Acute myocardial infarction	Crude	Large WHR was associated with increased risk of acute MI (OR: 2.50; 95%CI: 1.52-4.10). Overweight BMI, was also associated with increased risk (OR: 1.55; 95%CI: 0.88-2.69) but was not significant.

For peer review only

For peer review only - http://bmjopen.bmj.com/site/about/guidelines.xhtml

Author (study design)	Geographical location	No. of participants (% of women)	Mean (SD) age, years	Adiposity measurements	Mean follow-up, years	Outcomes	Adjustments	Results
Prabhakar et al., 2020[29] (case-control)	India	Cases: 144 (30.5) Controls: 144 (50.0)	*See categories in full-text*	BMI, WC	N/A	Stroke	Age, sex, smoking, alcohol, BP, diabetes, total cholesterol, HDL	Large WC in both men and women were not significantly associated with increased risk of stroke (OR: 1.33; 95%CI: 0.69-2.59 and OR: 1.06; 95%CI: 0.33-3.37 respectively). Being overweight was also not significantly associated with stroke (OR: 1.06; 95%CI: 0.33-3.37).
Shah et al., 2017[30] (case-control)	India	300 (22.0)	56.1 (10.7)	BMI, WC, WHR	N/A	CAD	Not provided	Overweight BMI significantly associated with risk of CAD (OR: 2.65; 95%CI: 1.49-4.72). Large WHR not significantly associated with risk of CAD (OR: 1.60; 95%CI: 0.97-2.66). High WC significantly associated with risk of CAD (OR: 1.71; 95%CI: 1.01-2.88).

For peer review only

For peer review only - http://bmjopen.bmj.com/site/about/guidelines.xhtml

Tables S4a, S4b, and S4c. Quality assessment of included studies

Performed using the Newcastle-Ottawa Scale adapted to cohort, case-control, and cross-sectional studies. Criteria that were met were marked with a 1, whereas criteria that were not met were marked with a 0.

S4a: COHORT STUDIES

First author, publication year	SELECTION				COMPARABILITY		OUTCOME			Total (9/9)
	Representative of exposed cohort	Selection of non-exposed	Exposure ascertainment	Outcome absent at start	Main factor controlled for	Additional factors controlled for	Assessment of outcomes	Sufficient follow-up time (>2 years)	Adequacy of follow-up	
Agrawal et al., 2014	1	1	1	0	1	1	1	0	1	6/9
Cameron et al., 2012	1	1	1	1	1	0	0	1	1	7/9

S4b: CASE-CONTROL STUDIES

First author, publication year	SELECTION				COMPARABILITY		OUTCOME			Total (9/9)
	Adequate case definition	Representativeness of cases	Selection of controls	Definition of controls	Main factor controlled for	Additional factors controlled	Ascertainment of exposure	Same ascertainment cases and controls	Non-response rate	
Latheef et al., 2018	1	0	0	1	1	0	1	1	0	5/9
Patil et al., 2004	1	1	0	1	1	0	1	1	0	6/9
Prabhakar et al., 2020	1	0	1	1	1	0	1	1	0	6/9
Nishtar et al., 2008	1	0	0	1	1	0	1	1	0	5/9
Shah et al., 2017	1	0	0	1	1	0	0	1	0	4/9

For peer review only - http://bmjopen.bmj.com/site/about/guidelines.xhtml

S4c: CROSS-SECTIONAL STUDIES

First author, publication year	SELECTION				COMPARABILITY		OUTCOME		Total (10/10)
	Representativeness of sample	Sample size	Ascertainment of exposure (max 2)	Non-response rate	Main factor controlled for	Additional factors controlled	Assessment of outcome (max 2)	Same ascertainment cases and controls	
Banerjee et al., 2021	1	1	0	0	1	1	1	1	6/10
Bodkhe et al., 2019	1	0	2	0	1	1	1	1	7/10
Bose et al., 2005	1	0	2	0	1	0	2	1	7/10
Bramhankar et al., 2021	1	0	0	0	1	1	1	1	5/10
Deshpande-Joshi et al., 2017	0	1	2	0	0	0	2	1	6/10
Dhall et al., 2018	0	0	2	0	0	0	2	1	5/10
Ganguli et al., 2013	1	0	2	0	1	1	2	1	8/10
Ghosh et al., 2007	0	0	2	0	0	0	2	1	5/10
Hazarika et al., 2004	1	1	2	0	1	1	2	1	9/10
Islam et al., 2016	1	1	2	0	1	1	2	1	9/10
Kaur et al., 2008	0	0	2	0	0	0	2	1	5/10
Kaur et al., 2012	0	1	2	0	1	0	2	1	7/10
Khanam et al., 2019	1	1	2	0	1	1	2	1	9/10
Meshram et al., 2012	0	1	2	0	1	1	2	1	8/10
Meshram et al., 2016	1	1	2	0	1	1	2	1	9/10
Shritaam et al., 2021	1	1	2	0	1	1	2	1	9/10
Simmons et al., 2021	0	0	1	0	1	1	1	1	5/10
Singh et al., 2012	0	0	2	0	1	0	2	1	6/10
Taing et al., 2016	1	0	2	0	0	0	2	1	6/10
Tselha et al., 2019	0	0	2	0	0	0	2	1	5/10
Venkatramana et al., 2002	1	0	2	0	1	1	2	1	8/10
Vikram et al., 2016	1	0	2	0	0	0	2	1	6/10
Yadav et al., 2008	0	1	2	1	1	1	2	1	9/10

For peer review only - http://bmjopen.bmj.com/site/about/guidelines.xhtml
For peer review only

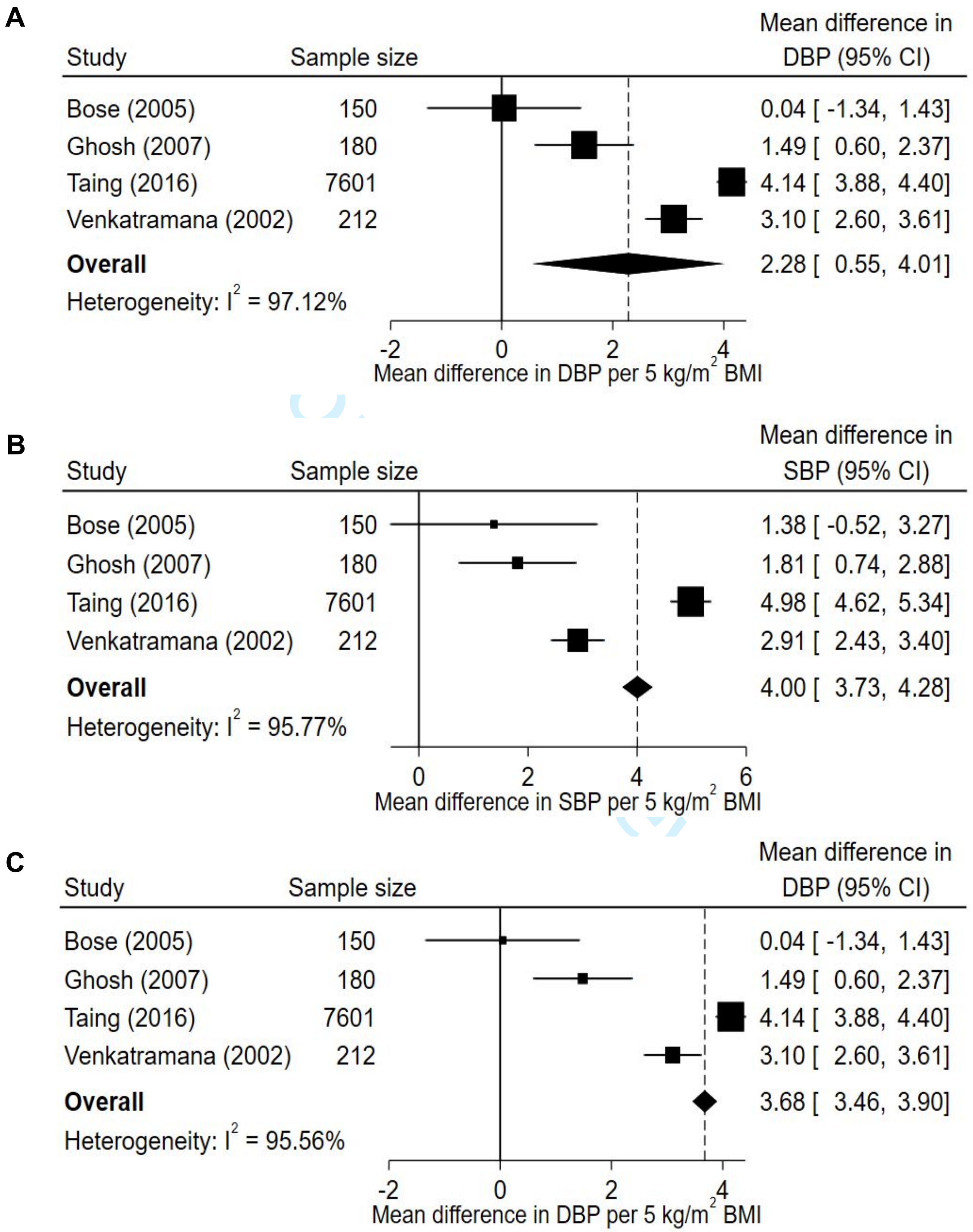

Figure S5. Mean change in systolic blood pressure (SBP, panel B) and diastolic blood pressure (DBP, panels A and C) per 5kg/m^2 higher body mass index (BMI)
Random effects (A) and fixed-effects (B and C) models were applied on four studies reporting associations of blood pressure and BMI. The total number of participants was 8,143.
Abbreviations: 95% confidence interval (95%CI).

For peer review only - http://bmjopen.bmj.com/site/about/guidelines.xhtml

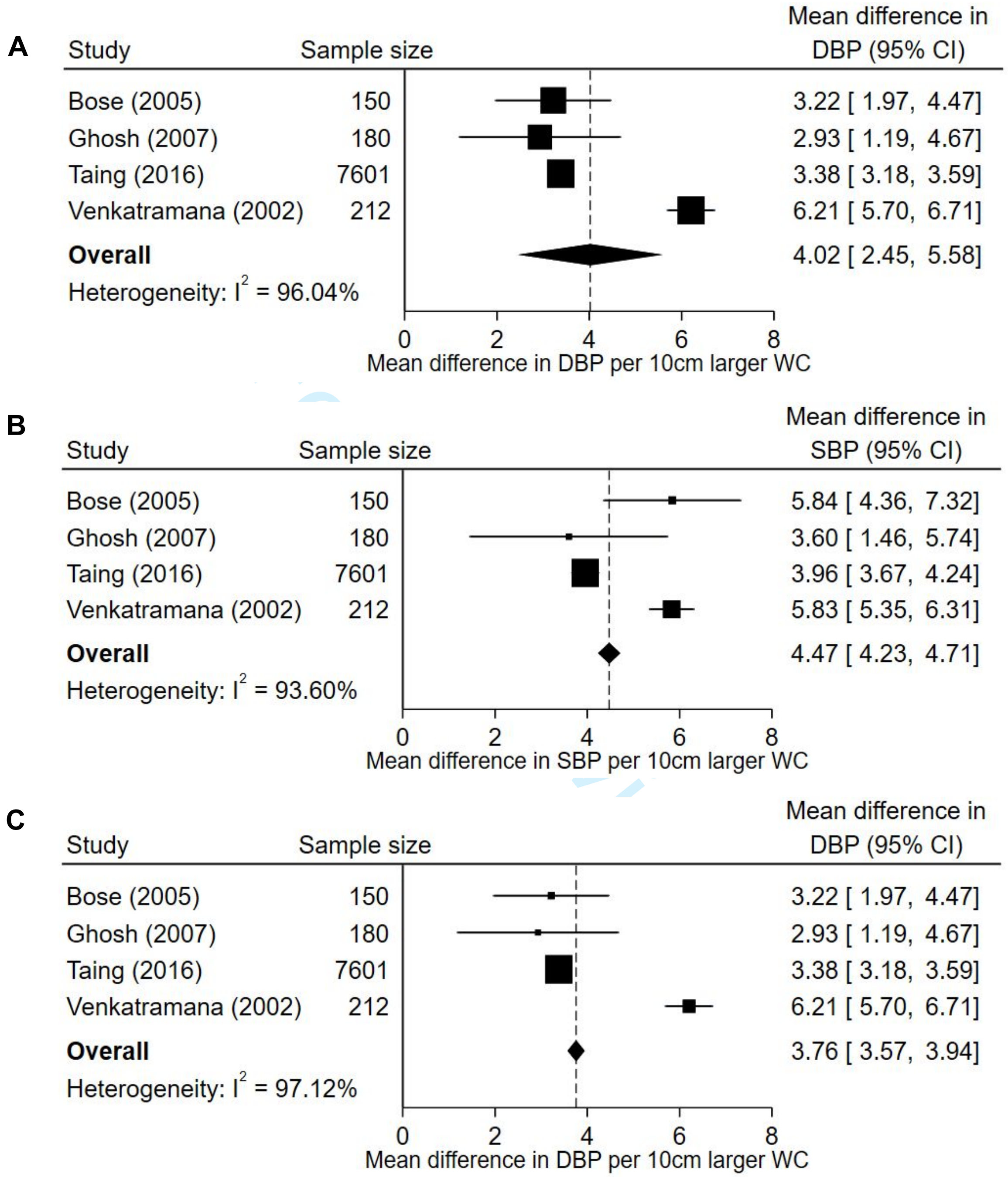

Figure S6. Mean change in systolic blood pressure (SBP, panel B) and diastolic blood pressure (DBP, panels A and C) per 10cm larger waist circumference (WC)
Random effects (A) and fixed-effects (B and C) models were applied on four studies reporting associations of blood pressure and WC. The total number of participants was 8,143.
Abbreviations: 95% confidence interval (95%CI).

For peer review only - http://bmjopen.bmj.com/site/about/guidelines.xhtml

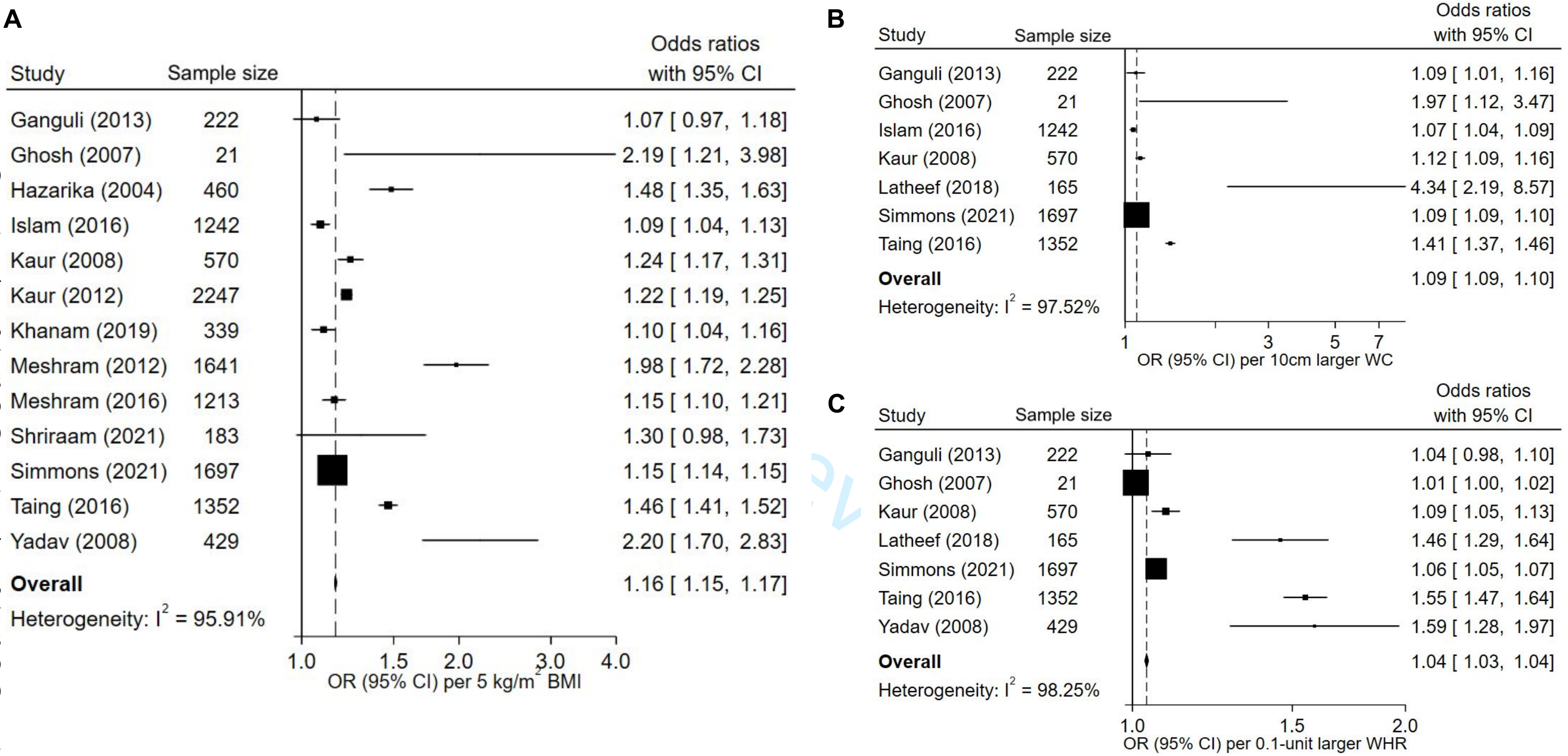

Figure S7. Odds ratio (OR) of hypertension per 5kg/m² higher body mass index (BMI, A), 10cm larger waist circumference (WC, B) and 0.1-units larger waist-to-hip ratio (WHR, C)
A fixed-effects model was used on 13 studies looking at BMI, seven studies looking at WC and seven studies looking at WHR. The total number of participants was 11,636 for panel A, 4,899 for B, and 4,456 or panel C. *Abbreviations: 95% confidence interval (95%CI).*

For peer review only - http://bmjopen.bmj.com/site/about/guidelines.xhtml

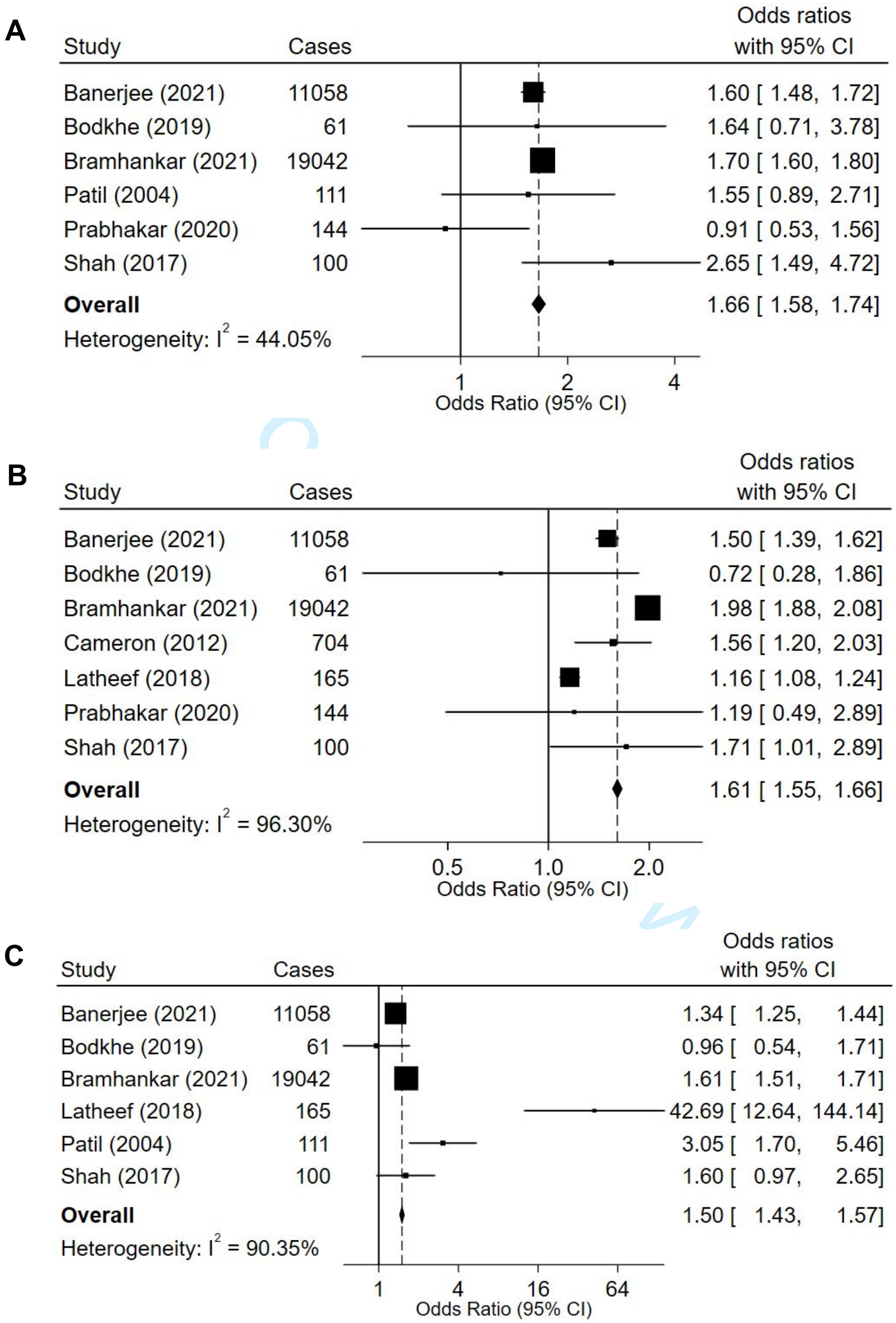

Figure S8. Odds ratio of cardiovascular disease for overweight vs. normal body mass index (BMI, panel A), large vs. normal waist circumference (WC, panel B), and large vs. normal waist-to-hip ratio (WHR, panel C)
A fixed-effects model was used on six studies looking at BMI, seven studies looking at WC, and six studies looking at WHR. *Abbreviations: 95% confidence interval (95%CI).*

For peer review only - http://bmjopen.bmj.com/site/about/guidelines.xhtml

S9. Supplemental results. Additional details on sensitivity analyses results

Results were not substantively different after sensitivity analyses. For the studies looking at the association of a 5kg/m^2 increase in BMI with HTN, removing the study by Meshram *et al*. decreased the odds ratio from 1.33 (95%CI: 1.18-1.51) to 1.28 (95%CI: 1.14-1.42).[12] Similar results were seen when removing the study by Yadav et al. (OR: 1.28; 95%CI: 1.15-1.43).[21] Individual removal of several other studies resulted in an increase in magnitude of associations to 1.36 (1.20-1.55).[5,8,11,13,15] For studies looking at the odds of HTN per 10cm larger WC, removing the study by Simmons et al. resulted in an increase in estimates from 1.33 (1.04-1.69) to 1.57 (1.28-1.93).[15] For studies looking at the odds of HTN per 0.1-unit larger WHR, removing the studies by Ghosh et al. [6] increased the strength of associations from 1.18 (1.02-1.37) to 1.26 (1.07-1.48).

For peer review only

For peer review only - http://bmjopen.bmj.com/site/about/guidelines.xhtml

DATA SUPPLEMENT REFERENCE LIST

1. Agrawal P, Gupta K, Aboyans V, Agrawal S. Women's health in India: the role of body mass index. Health Care Women Int. 2014;36(3):320–41.

2. Bose K, Ghosh A, Roy S, Gangopadhyay S. The relationship of age, body mass index and waist circumference with blood pressure in Bengalee Hindu male jute mill workers of Belur, West Bengal, India. Anthropology Anz. 2005;63(2):205–12.

3. Deshpande-Joshi S, Rao S. Differential risk of hypertension among lean and non-lean rural subjects in relation to decadal changes in anthropometry. J Am Coll Nutr. 2018;37(5):380–6.

4. Dhall M, Devi K, Nilupher A, Gupta U, Tyagi R, Kapoor S. Hypertension and its correlate with general and central adiposity: A study among urban population of Delhi. Diabetes Metab Syndr. 2018;12(6):881–4.

5. Ganguli D, Das N, Saha I, Chaudhuri D, Ghosh S, Dey S. Risk factors for hypertension in a population-based sample of postmenopausal women in Kolkata, West Bengal, India. Asia Pac J Public Health. 2013;25(5):388–97.

6. Ghosh J, Bandyopadhyay A. Comparative evaluation of obesity measures: relationship with blood pressure and hypertension. Singapore Med J. 2007;48(3):232–5.

7. Hazarika N, Narain K, Biswas D, Kalita H, Mahanta J. Hypertension in the native rural population of Assam. Natl Med J India. 2004;17:300–4.

8. Islam FMA, Bhuiyan A, Chakrabarti R, Rahman MA, Kanagasingam Y, Hiller JE. Undiagnosed hypertension in a rural district in Bangladesh: The Bangladesh Population-based Diabetes and Eye Study (BPDES). J Hum Hypertens. 2016;30(4):252–9.

9. Kaur P, Radhakrishnan E, Sankarasubbaiyan S, Rao S, Kondalsamy-Chennakesavan S, Rao T, et al. A Comparison of Anthropometric Indices for Predicting Hypertension and Type 2 Siabetes in a Male Industrial Population of Chennai, South India. Ethnicity and Disease. 2008;18:31–26.

10. Kaur P, Rao S, Radhakrishnan E, Rajasekar D, Gupte M. Prevalence, awareness, treatment, control and risk factors for hypertension in a rural population in South India. Int J Public Health. 2012;57(1):87–94.

11. Khanam R, Ahmed S, Rahman S, Kibria G, Syed J, Khan A, et al. Prevalence and factors associated with hypertension among adults in rural Sylhet district of Bangladesh: a cross-sectional study. BMJ Open [Internet]. 2019 [cited 2022 Jul 24];9(10). Available from: https://bmjopen.bmj.com/lookup/doi/10.1136/bmjopen-2018-026722

For peer review only - http://bmjopen.bmj.com/site/about/guidelines.xhtml

12. Meshram II, Arlappa N, Balkrishna N, Rao KM, Laxmaiah A, Brahmam GNV. Prevalence of hypertension, its correlates and awareness among adult tribal population of Kerala state, India. J Postgrad Med. 2012;58(4):255–61.

13. Meshram II, Vishnu Vardhana Rao M, Sudershan Rao V, Laxmaiah A, Polasa K. Regional variation in the prevalence of overweight/obesity, hypertension and diabetes and their correlates among the adult rural population in India. Br J Nutr. 2016;115(7):1265–72.

14. Shriraam V, Mahadevan S, Arumugam P. Prevalence and risk factors of diabetes, hypertension and other non-communicable diseases in a tribal population in South India. Indian J Endocr Metab. 2021;25(4):313.

15. Simmons S, Hagan Jr. J, Schack T. The influence of anthropometric indices and intermediary determinants of hypertension in Bangladesh. IJERPH [Internet]. 2021 [cited 2022 Jul 24];18(11). Available from: https://www.mdpi.com/1660-4601/18/11/5646

16. Singh R, Mukherjee M, Kumar R, Singh R, Pal R. Study of risk factors of coronary heart disease in urban slums of Patna. Nepal J Epidemiology. 2012;2(3):205–12.

17. Taing K, Farkouh M, Moineddin R, Tu J, Jha P. Age and sex-specific associations of anthropometric measures of adiposity with blood pressure and hypertension in India: a cross-sectional study. BMC Cardiovasc Disord. 2016;16(1).

18. Tselha N, Shimrah C, Kulshreshtha M, Devi N. Association between hypertension and adiposity indicators: A study among the Muslim population of Uttar Pradesh. Diabetes Metab Syndr. 2019;13(4):2335–8.

19. Venkatramana P, Reddy P. Association of overall and abdominal obesity with coronary heart disease risk factors: comparison between urban and rural Indian men. Asia Pac J Clin Nutr. 2002;11(1):66–71.

20. Vikram N, Latifi A, Misra A, Luthra K, Bhatt S, Guleria R, et al. Waist-to-height ratio compared to standard obesity measures as predictor of cardiometabolic risk factors in Asian Indians in North India. Metab Syndr Relat Disord. 2016;14(10):492–9.

21. Yadav S, Boddula R, Genitta G, Bhatia V, Bansal B, Kongara S, et al. Prevalence & risk factors of pre-hypertension & hypertension in an affluent north Indian population. Indian J Med Res. 2008;(128):712–20.

22. Banerjee S, Kumar P, Srivastava S, Banerjee A. Association of anthropometric measures of obesity and physical Activity with ardiovascular diseases among older adults: evidence from a cross-sectional survey, 2017–18. Gaipov A, editor. PLoS ONE [Internet]. 2021 [cited 2022 Jul 24];16(12). Available from: https://dx.plos.org/10.1371/journal.pone.0260148

For peer review only - http://bmjopen.bmj.com/site/about/guidelines.xhtml

23. Bodkhe S, Jajoo S, Jajoo U, Ingle S, Gupta S, Taksande B. Epidemiology of confirmed coronary heart disease among population older than 60 years in rural central India—A community-based cross-sectional study. Indian Heart Journal. 2019;71(1):39–44.

24. Bramhankar M, Pandey M, Rana G, Rai B, Mishra N, Shukla A. An assessment of anthropometric indices and its association with NCDs among the older adults of India: evidence from LASI Wave-1. BMC Public Health. 2021;21(1):1357.

25. Cameron A, Magliano D, Shaw J, Zimmet P, Carstensen B, Alberti K, et al. The influence of hip circumference on the relationship between abdominal obesity and mortality. Intl J of Epi. 2012;41(2):484–94.

26. Latheef SAA, Subramanyam G, Reddy BM. Utility of anthropometric traits and indices in predicting the risk of coronary artery disease in the adult men of southern Andhra Pradesh. Indian Heart J. 2018;70(3):S133–9.

27. Nishtar S, Wierzbicki A, Lumb P, Lambert-Hammill M, Turner C, Crook M, et al. Waist-hip ratio and low HDL predict the risk of coronary artery disease in Pakistanis. Current Medical Research and Opinion. 2008;20(1):55–62.

28. Patil S, Joshi R, Gupta G, Reddy M, Pai M, Kalantri S. Risk factors for acute myocardial infarction in a rural population of central India: A hospital-based case–control study. Nat Med J India. 2004;17(4).

29. Prabhakar S, Suravarapu S, Mathai D, Renangi S, Challa S. Risk factors for stroke in rural population of Telangana State of India, an unmatched case-control study. J Neurosci Rural Pract. 2020;11(3):448–53.

30. Shah M, Mazumdar V, Patel S, Baxi R, Shringarpure K. A case control study of risk factors of coronary heart disease among patients admitted at tertiary hospital in western India. Australasian Medical Journal. 2017;10(05):381–8.

For peer review only

For peer review only - http://bmjopen.bmj.com/site/about/guidelines.xhtml

PRISMA 2020 Checklist

Section and Topic	Item #	Checklist item	Location where item is reported
**TITLE**			
Title	1	Identify the report as a systematic review.	Page 1
**ABSTRACT**			
Abstract	2	See the PRISMA 2020 for Abstracts checklist.	Pages 2-3
**INTRODUCTION**			
Rationale	3	Describe the rationale for the review in the context of existing knowledge.	Pages 5-6
Objectives	4	Provide an explicit statement of the objective(s) or question(s) the review addresses.	Page 6
**METHODS**			
Eligibility criteria	5	Specify the inclusion and exclusion criteria for the review and how studies were grouped for the syntheses.	Page 7
Information sources	6	Specify all databases, registers, websites, organisations, reference lists and other sources searched or consulted to identify studies. Specify the date when each source was last searched or consulted.	Page 6
Search strategy	7	Present the full search strategies for all databases, registers, and websites, including any filters and limits used.	Supplement S1
Selection process	8	Specify the methods used to decide whether a study met the inclusion criteria of the review, including how many reviewers screened each record and each report retrieved, whether they worked independently, and if applicable, details of automation tools used in the process.	Page 7
Data collection process	9	Specify the methods used to collect data from reports, including how many reviewers collected data from each report, whether they worked independently, any processes for obtaining or confirming data from study investigators, and if applicable, details of automation tools used in the process.	Page 7
Data items	10a	List and define all outcomes for which data were sought. Specify whether all results that were compatible with each outcome domain in each study were sought (e.g. for all measures, time points, analyses), and if not, the methods used to decide which results to collect.	Pages 6-7
	10b	List and define all other variables for which data were sought (e.g. participant and intervention characteristics, funding sources). Describe any assumptions made about any missing or unclear information.	Pages 6-7
Study risk of bias assessment	11	Specify the methods used to assess risk of bias in the included studies, including details of the tool(s) used, how many reviewers assessed each study and whether they worked independently, and if applicable, details of automation tools used in the process.	Pages 7-8
Effect measures	12	Specify for each outcome the effect measure(s) (e.g. risk ratio, mean difference) used in the synthesis or presentation of results.	Page 7
Synthesis methods	13a	Describe the processes used to decide which studies were eligible for each synthesis (e.g. tabulating the study intervention characteristics and comparing against the planned groups for each synthesis (item #5)).	N/A
	13b	Describe any methods required to prepare the data for presentation or synthesis, such as handling of missing summary statistics, or data conversions.	Pages 8-9
	13c	Describe any methods used to tabulate or visually display results of individual studies and syntheses.	Page 8
	13d	Describe any methods used to synthesize results and provide a rationale for the choice(s). If meta-analysis was performed, describe the model(s), method(s) to identify the presence and extent of statistical heterogeneity, and software package(s) used.	Pages 8-9
	13e	Describe any methods used to explore possible causes of heterogeneity among study results (e.g. subgroup analysis, meta-regression).	Pages 8-9
	13f	Describe any sensitivity analyses conducted to assess robustness of the synthesized results.	N/A
Reporting bias assessment	14	Describe any methods used to assess risk of bias due to missing results in a synthesis (arising from reporting biases).	Pages 7-8
Certainty assessment	15	Describe any methods used to assess certainty (or confidence) in the body of evidence for an outcome.	N/A

For peer review only

PRISMA 2020 Checklist

Section and Topic	Item #	Checklist item	Location where item is reported
**RESULTS**			
Study selection	16a	Describe the results of the search and selection process, from the number of records identified in the search to the number of studies included in the review, ideally using a flow diagram.	Page 9
	16b	Cite studies that might appear to meet the inclusion criteria, but which were excluded, and explain why they were excluded.	Figure 1
Study characteristics	17	Cite each included study and present its characteristics.	Pages 9-10; Supplement S2 and S3
Risk of bias in studies	18	Present assessments of risk of bias for each included study.	Page 13; Supplement S4a, S4b, S4c
Results of individual studies	19	For all outcomes, present, for each study: (a) summary statistics for each group (where appropriate) and (b) an effect estimate and its precision (e.g. confidence/credible interval), ideally using structured tables or plots.	Supplement S2 and S3
Results of syntheses	20a	For each synthesis, briefly summarise the characteristics and risk of bias among contributing studies.	Page 13
	20b	Present results of all statistical syntheses conducted. If meta-analysis was done, present for each the summary estimate and its precision (e.g. confidence/credible interval) and measures of statistical heterogeneity. If comparing groups, describe the direction of the effect.	Pages 10-11; Figures 2, 3, 4; Supplement S5, S6, S7, S8
	20c	Present results of all investigations of possible causes of heterogeneity among study results.	N/A
	20d	Present results of all sensitivity analyses conducted to assess the robustness of the synthesized results.	Page 13; Supplement S9
Reporting biases	21	Present assessments of risk of bias due to missing results (arising from reporting biases) for each synthesis assessed.	Page 13; Figure 5
Certainty of evidence	22	Present assessments of certainty (or confidence) in the body of evidence for each outcome assessed.	N/A
**DISCUSSION**			
Discussion	23a	Provide a general interpretation of the results in the context of other evidence.	Pages 12-14
	23b	Discuss any limitations of the evidence included in the review.	Pages 12-14
	23c	Discuss any limitations of the review processes used.	Pages 14-15
	23d	Discuss implications of the results for practice, policy, and future research.	Pages 12-15
**OTHER INFORMATION**			
Registration and protocol	24a	Provide registration information for the review, including register name and registration number, or state that the review was not registered.	Page 6
	24b	Indicate where the review protocol can be accessed, or state that a protocol was not prepared.	Page 6
	24c	Describe and explain any amendments to information provided at registration or in the protocol.	N/A
Support	25	Describe sources of financial or non-financial support for the review, and the role of the funders or sponsors in the review.	Page 18
Competing interests	26	Declare any competing interests of review authors.	Page 18

For peer review only

PRISMA 2020 Checklist

Section and Topic	Item #	Checklist item	Location where item is reported
Availability of data, code and other materials	27	Report which of the following are publicly available and where they can be found: template data collection forms; data extracted from included studies; data used for all analyses; analytic code; any other materials used in the review.	Page 18

From: Page MJ, McKenzie JE, Bossuyt PM, Boutron I, Hoffmann TC, Mulrow CD, et al. The PRISMA 2020 statement: an updated guideline for reporting systematic reviews. BMJ 2021;372:n71. doi: 10.1136/bmj.n71

For more information, visit: http://www.prisma-statement.org/

For peer review only

BMJ Open

Associations of General and Central Adiposity with Hypertension and Cardiovascular Disease Among South Asian Populations: A Systematic Review and Meta-Analysis

Journal:	*BMJ Open*
Manuscript ID	bmjopen-2023-074050.R1
Article Type:	Original research
Date Submitted by the Author:	31-Oct-2023

Complete List of Authors:	Re, Federica; University of Oxford, Clinical Trial Service Unit and Epidemiological Studies Unit (CTSU), Nuffield Department of Population Health<br>Oguntade, Ayodipupo; University of Oxford, Clinical Trial Service Unit and Epidemiological Studies Unit (CTSU), Nuffield Department of Population Health<br>Bohrmann, Bastian; University of Oxford, Clinical Trial Service Unit and Epidemiological Studies Unit (CTSU), Nuffield Department of Population Health<br>Bragg, Fiona; University of Oxford, Clinical Trial Service Unit and Epidemiological Studies Unit (CTSU), Nuffield Department of Population Health; University of Oxford, MRC Population Health Research Unit, Nuffield Department of Population Health<br>Carter, Jennifer ; University of Oxford, Clinical Trial Service Unit and Epidemiological Studies Unit (CTSU), Nuffield Department of Population Health; University of Oxford, MRC Population Health Research Unit, Nuffield Department of Population Health

**Primary Subject Heading**:	Cardiovascular medicine
Secondary Subject Heading:	Epidemiology, Global health, Public health
Keywords:	Systematic Review, Hypertension < CARDIOLOGY, EPIDEMIOLOGY

SCHOLARONE™
Manuscripts

I, the Submitting Author has the right to grant and does grant on behalf of all authors of the Work (as defined in the below author licence), an exclusive licence and/or a non-exclusive licence for contributions from authors who are: i) UK Crown employees; ii) where BMJ has agreed a CC-BY licence shall apply, and/or iii) in accordance with the terms applicable for US Federal Government officers or employees acting as part of their official duties; on a worldwide, perpetual, irrevocable, royalty-free basis to BMJ Publishing Group Ltd ("BMJ") its licensees and where the relevant Journal is co-owned by BMJ to the co-owners of the Journal, to publish the Work in this journal and any other BMJ products and to exploit all rights, as set out in our licence.

The Submitting Author accepts and understands that any supply made under these terms is made by BMJ to the Submitting Author unless you are acting as an employee on behalf of your employer or a postgraduate student of an affiliated institution which is paying any applicable article publishing charge ("APC") for Open Access articles. Where the Submitting Author wishes to make the Work available on an Open Access basis (and intends to pay the relevant APC), the terms of reuse of such Open Access shall be governed by a Creative Commons licence – details of these licences and which Creative Commons licence will apply to this Work are set out in our licence referred to above.

Other than as permitted in any relevant BMJ Author's Self Archiving Policies, I confirm this Work has not been accepted for publication elsewhere, is not being considered for publication elsewhere and does not duplicate material already published. I confirm all authors consent to publication of this Work and authorise the granting of this licence.

For peer review only

Associations of General and Central Adiposity with Hypertension and Cardiovascular Disease Among South Asian Populations: A Systematic Review and Meta-Analysis

Federica RE[1] MSc, Ayodipupo S. OGUNTADE[1] MSc, Bastian BOHRMANN[1] MSc, Fiona BRAGG[1,2] DPhil, Jennifer L. CARTER[1] PhD

[1]Clinical Trial Service Unit and Epidemiological Studies Unit (CTSU), Nuffield Department of Population Health, University of Oxford, Oxford, UK.

[2]MRC Population Health Research Unit, Nuffield Department of Population Health, University of Oxford, Oxford, UK.

Address for correspondence
Dr Jennifer Carter
Nuffield Department of Population Health
Big Data Institute
Roosevelt Drive, Oxford
OX3 7LF, UK
jennifer.carter@ndph.ox.ac.uk

For peer review only

Short title: Adiposity in hypertension and CVD risk among South Asians

Word count total: 4,371

Word count abstract: 296

Total tables in main text: 0

Total figures in main text: 5

For peer review only - http://bmjopen.bmj.com/site/about/guidelines.xhtml

ABSTRACT

Background: The relevance of measures of general and central adiposity for cardiovascular disease (CVD) risks in populations of European descent is well-established. However, it is less well-characterised in South Asian populations, who characteristically manifest larger waist circumferences (WC) for equivalent body mass index (BMI). This systematic review and meta-analysis provide an overview of the literature on the association of different anthropometric measures with CVD risk among South Asians.

Methodology: MEDLINE and Embase were searched from 1990 to the present for studies in South Asian populations investigating associations of two or more adiposity measures with CVD. Random-effects meta-analyses were conducted on the associations of BMI, WC, and waist-to-hip ratio (WHR) with blood pressure, hypertension, and CVD. Quality assessment was performed using the Newcastle-Ottawa scale.

Results: Titles and abstracts were screened for 7,327 studies, yielding 147 full-text reviews. The final sample (n=30) included two prospective, five case-control, and 23 cross-sectional studies. Studies reported generally higher risks of hypertension and CVD at higher adiposity levels. The pooled mean difference in systolic blood pressure (SBP) per 5 kg/m^2 higher BMI was 3 mmHg (2.90 [95%CI:1.30-4.50]), and 5 mmHg (6.31; [4.81-7.81]) per 13 cm larger WC. The odds ratio (OR) of hypertension per 5 kg/m^2 higher BMI was 1.33 (95%CI:1.18-1.51), 1.45 (1.05-1.98) per 13 cm larger WC, and 1.22 (1.04-1.41) per 0.1-unit larger WHR. Pooled risk of CVD for BMI-defined overweight versus healthy-weight was 1.65 (95%CI:1.55-1.75), and 1.48 (1.21-1.80) and 2.51 (0.94-6.69) for normal versus large WC and WHR, respectively. Study quality was average with significant heterogeneity.

Conclusions: Measures of both general and central adiposity had similar, strong positive associations with the risk of CVD in South Asians. Larger prospective studies

For peer review only - http://bmjopen.bmj.com/site/about/guidelines.xhtml

are required to clarify which measures of body composition are more informative for targeted CVD primary prevention in this population.

For peer review only

For peer review only - http://bmjopen.bmj.com/site/about/guidelines.xhtml

STRENGTHS AND LIMITATIONS OF THIS STUDY

- A broad literature search, robust duplicate and blinded screening, and a firm quality assessment represent major strengths of this study.
- Sensitivity analyses excluding large studies contributing the most to the models in the meta-analyses showed that associations remained largely unchanged.
- While the review aims to assess risk across South Asia, most studies were conducted in India, thus limiting generalisability of findings to the rest of the sub-continent.
- Cross-sectional design of studies included in the review increases the possibility of reverse causality.

For peer review only

For peer review only - http://bmjopen.bmj.com/site/about/guidelines.xhtml

INTRODUCTION

Being overweight or obese is one of the leading risk factors for premature mortality, estimated to account for up to 4.7 million deaths yearly.[1–3] The Global BMI Mortality Collaboration, a meta-analysis of 239 prospective studies, reported higher risk of cardiovascular disease (CVD) with higher body mass index (BMI) across all regions, showing that overweight individuals (BMI >25kg/m^2) experienced 49% higher risk per 5kg/m^2 higher BMI than individuals whose weight was within the healthy range (BMI 18.5-24.9kg/m^2).[4] Further meta-analyses across Europe, North America, and East Asia have reached similar conclusions.[5–7] The shape and strength of the association between BMI and CVD risk, however, has been found to differ across certain subgroups, with weaker associations among South Asians– though few studies have focused on these populations.[8,9]

A prospective cohort study of 0.5 million adults from the city of Chennai, India, found that blood pressure was strongly and positively associated with coronary heart disease (CHD) mortality (risk ratio [RR]: 1.70 [95%CI: 1.60-1.80] per 20mmHg higher usual SBP), but that BMI was little related to CHD mortality, despite increased BMI being a strong determinant of increased systolic blood pressure (SBP).[8] Moreover, the Asia Cohort Consortium observed positive associations between overweight BMI and CVD death, but the relation was substantially weaker and not statistically significant in South Asians compared to East Asians (South Asians: hazard ratio [HR] 1.03 [95%CI: 0.93-1.15] vs. East Asians: HR: 1.09 [95%CI: 1.03-1.15]).[10] The Global BMI Collaboration concluded similar findings, describing that, among South Asians, the risk of CVD per 5kg/m^2 higher BMI in overweight individuals was 10% and not statistically significant (HR: 1.10 [95%CI: 0.83-1.46]). This was considerably weaker than the associations observed in European populations (HR: 1.56 [95%CI: 1.54-1.58]).[4]

There is some evidence suggesting that South Asians are at higher risk of diabetes and CVD compared to Western populations.[11] However, the underlying pathophysiology leading to ethnic variations in the prevalence of hypertension (HTN) and the risk of CVD is poorly understood, although differences in body fat composition and distribution may

For peer review only - http://bmjopen.bmj.com/site/about/guidelines.xhtml

explain these discrepancies. As an anthropometric measure, BMI does not distinguish between central and peripheral adiposity, or between fat and lean mass. Some evidence suggests that centrally distributed visceral fat and ectopic fat are associated with cardiovascular outcomes, independently of BMI.[12,13] Furthermore, there are marked differences between central and general adiposity in their associations with fasting glucose, diabetes, and blood pressure, which lie on the causal pathway for CVD.[12,14] Therefore, research needs to assess whether measures of central adiposity are more important markers of disease risk in South Asian populations.

There is mixed evidence on whether higher BMI is associated with higher risk of CVD among South Asians and on which anthropometric measures are more strongly related with CVD risk in these populations. Hence, the purpose of this review is to provide an overview of the literature on the shape and strength of the association of BMI and CVD, including hypertension, among South Asians and to understand whether alternative anthropometric measures are better indicators of adiposity-related CVD risk in this geographical area compared to BMI. Results can be used to develop research which better characterizes the importance of adipose tissue and its distribution in CVD risk. Over time, this can help develop targeted preventative interventions and minimize health disparities.

METHODS

Search strategy

This systematic review followed the Cochrane Collaboration methods and adhered to the PRISMA reporting recommendations.[15] A predetermined review protocol was registered in the PROSPERO database (CRD42022308682). MEDLINE and Embase were searched combining terms relating to central and general adiposity and cardiovascular outcomes. Further terms were included to select for the geographical area of interest, namely South Asia (Afghanistan, Bangladesh, Bhutan, India, Maldives, Nepal, Pakistan, and Sri Lanka). The MeSH terms were chosen from the thesaurus used for indexing the subject headings. Full details of the search strategy for MEDLINE and Embase are shown in the **Data Supplement S1**. The search was limited to studies

For peer review only - http://bmjopen.bmj.com/site/about/guidelines.xhtml

conducted on adults (18+years) and published in English between 1st January 1990 and 1st January 2023.

Eligibility criteria

Eligible studies included at least one measure of general (BMI, kg/m^2) and one measure of central adiposity (WC, WHR, waist-to-height ratio [WHtR], cm) as the exposure, and at least one cardiovascular outcome: SBP (mmHg), diastolic blood pressure (DBP, mmHg), hypertension (persistent blood pressure >140/90 mmHg), coronary artery disease (CAD), peripheral vascular disease (PVD) or CVD incidence/mortality.

The search included cross-sectional, case-control, and cohort studies. To limit potential publication bias from the inclusion of small studies with chance findings that reported stronger-than-average results, studies examining clinical endpoints (e.g., CVD, hypertension) were included in the review only if they included at least 50 events, while those examining continuous outcomes (e.g., blood pressure) were included if they included at least 100 participants. Studies that solely performed correlational analyses, those conducted among participants with prevalent diseases, or those on South Asian populations living outside of South Asia were excluded.

Study selection and data extraction

Studies were imported into Covidence, an online systematic review management platform, for abstract and full-text screening.[16] After removal of duplicates, titles and abstracts were reviewed independently by two reviewers (FR and ASO). Studies that did not meet the inclusion criteria were excluded. The full texts of the remaining papers were reviewed independently by the same reviewers. Disagreements were resolved by discussion and consensus between the two reviewers or, where necessary, by involving a third reviewer (JLC). A full list of included studies is reported in the **Data Supplement (S2 and S3)**. Associations were recorded for each study.

For peer review only - http://bmjopen.bmj.com/site/about/guidelines.xhtml

Quality assessment

Quality of included studies was assessed using validated cross-sectional, case-control, and cohort adaptations of the Newcastle-Ottawa scale.[17,18] Studies that fulfilled a criterion were awarded a point for that criterion, while no point was awarded if the criterion was not fulfilled. Cohort and case-control studies could be awarded a maximum of nine points, while cross-sectional studies could be awarded a maximum of ten points. Studies were considered of high quality if they met at least seven out of nine criteria for cohort and case control studies, and eight out of ten criteria for cross-sectional studies. The quality of a study did not determine its inclusion in the systematic review or meta-analysis. Details of the quality assessment are available in the **Data Supplement** (**S4a/S4b/S4c**).

Data synthesis and analysis

For the dose-response meta-analyses, summary RR (95% CIs) per 5kg/m^2 higher BMI, 13cm higher WC, and 0.1-unit higher WHR were calculated using random effects models due to substantial heterogeneity of the included studies. BMI was presented as a 5 kg/m^2 change to allow comparability with other large-scale studies which have used the same units.[4,10] Associations with WC and WHR were compared to those of BMI by scaling the measures to the same SD unit change. Scaling factors were based on the mean and SD reported by Taing *et al.*, 2016 since this study had the largest sample size (n=7,601) and included all three adiposity measures of interest.[19] The pooled BMI SD in the study by Taing *et al.*, 2016 is 4.6 kg/m^2, so a change in 5 kg/m^2 represents a 1.087 SD change. Accordingly, central adiposity measures were then scaled to the same SD change as 5 kg/m^2 BMI, which corresponded to a 13 cm increase in WC and 0.1-unit change in WHR. Additional fixed-effects models were also calculated. Formulas for random and fixed effects models are included in the Data Supplement **(Data Supplement S5)**. For each study, the risk estimates from the model including the greatest number of confounders, but not intermediate factors (e.g., diabetes, left ventricular hypertrophy, etc), were used. The average of the natural logarithm of the RRs was calculated.[20] In cases where studies provided RRs (95%CIs) per unit higher adiposity measure, these were scaled to the desired units by exponentiating the RR

For peer review only - http://bmjopen.bmj.com/site/about/guidelines.xhtml
For peer review only

(95%CIs) to the power of desired units. When studies only reported RRs separately for different subgroups (e.g., age, sex, or ethnicity), these subgroup estimates were combined using a fixed-effects model to obtain an overall estimate. Each study was thus only represented once in each main meta-analysis.

Where studies reported estimates for categories of anthropometric measures, estimates were log-transformed and used to calculate study-specific slopes and 95% CIs across categories of anthropometric measures, as described by Greenland and Longnecker, to generate overall study-specific RRs.[21,22] Where studies only reported total cases and controls, total numbers were divided evenly across the categories.[23] The mean or median of each category of each anthropometric measure was assigned to the corresponding RRs. For studies that did not report the mean or median of the anthropometric measures, the midpoint of the range of such categories was used as the mean. When the lowest or highest category was open-ended, the width of the interval was assumed to be the same as that of the adjacent category.[23] A likelihood ratio test was used to test nonlinearity by assessing the difference between the linear and non-linear models.

Heterogeneity between studies was determined using a Q-test, and I^2 statistics were used to denote the percentage of total variability due to between-study heterogeneity. I^2 >70% indicated high heterogeneity. To assess the robustness of the overall estimates, sensitivity analyses were undertaken removing one study at a time to determine whether results were influenced by large studies or studies with extreme results. Publication bias and small study effects were examined by inspecting funnel plots for asymmetry and with Egger's test. Analyses were conducted using Stata/MP 17.0 (StataCorp LLC, College Station, Texas, USA).

RESULTS

The initial search, after duplicate removal, included 7,327 studies. Of these, 30 were included in this review. **Figure 1** shows the number of papers excluded at each stage of the review process. At the screening stage, most exclusions were of studies looking at a

For peer review only - http://bmjopen.bmj.com/site/about/guidelines.xhtml

single anthropometric measure only or those taking place in geographical regions not included in the review. On full-text assessment, most exclusions (n=85) were of studies performing only correlation analyses between anthropometric measures and CVD incidence. The number of participants in included studies varied between 140 and 59,037.[24,25] There were 25 (83.3%) included studies in India[19,24–47] and 3 (10%) in Bangladesh[48–50]. The remaining two studies were conducted in Mauritian[51] and Pakistani[52] populations, respectively. The final analyses included two prospective cohort studies (one looking at hypertension[26] and one looking at CVD mortality[51]), four case-control studies[44–47] (looking at CVD outcomes), and 24 cross-sectional studies[19,24,25,27–43,48–50,52] (looking at hypertension outcomes). In terms of exposure variables, 29 studies included BMI, 27 studies used WC, 21 studies used WHR, and 10 studies additionally used other measures, including waist-to-height ratio (n=6) and hip circumference (n=2). Overall, 18 studies looked at hypertension[24,26,28–38,40,41,48–50], with or without blood pressure, eight looked at CVD[25,42–47,52], three looked at blood pressure alone[19,27,39], and one study looked at CVD mortality[51].

Associations between anthropometric indices and blood pressure

A total of four studies were included in the analysis of blood pressure (SBP/DBP). Of the four studies looking at the relationship between BMI and blood pressure, all studies concluded that higher BMI was related to higher blood pressure, with stronger associations with SBP than DBP. This was reflected in the meta-analysis, which showed that the pooled mean difference per 5kg/m^2 higher BMI was 3 mmHg (2.90 [95%CI: 1.30-4.50]) for SBP (**Figure 2**) and 2 mmHg (2.28 [95%CI: 0.55-4.01]) for DBP (**Data Supplement S6**). For WC, the pooled mean change in blood pressure per 13cm larger WC was almost 5 mmHg (6.31 [95%CI: 4.81-7.81]) for SBP (**Figure 2**) and 5 mmHg (5.18 [95%CI: 3.18-7.18]) for DBP (**Data Supplement S7**). Associations were not statistically significant per 0.1-unit change in WHR (SBP: 2.27 [95%CI: -1.62-6.16]; DBP: 1.94 [95%CI: -1.42-5.29]). Findings of fixed-effects models are reported in the **Data Supplement (S6 and S7)**.

For peer review only - http://bmjopen.bmj.com/site/about/guidelines.xhtml

Associations between anthropometric indices and hypertension

All studies looking at hypertension risk reported positive associations between measures of general adiposity (BMI) and/or central adiposity (WC, WHR) with the risk of hypertension. Five cross-sectional studies concluded that the risk of hypertension was higher with a high BMI (≥25 kg/m^2) compared to a large WC (≥80cm in females, ≥90 in males) or WHR (≥0.8 in females, ≥1.0 in males). Eleven cross-sectional studies reported that measures of central adiposity, compared to BMI, showed stronger associations with hypertension. Of these, seven reported stronger associations with WC[29,33,36,41,48–50], and four with WHR[28,37,38,40]. The pooled odds ratio of hypertension per 5kg/m^2 higher BMI was 1.33 (95%CI: 1.18-1.51; **Figure 3A**). It was stronger for a 13cm larger waist circumference (OR: 1.45 [95%CI: 1.05-1.98]; **Figure 3B**), but weaker for a 0.1-unit larger WHR (OR: 1.22 [95%CI: 1.05-1.41]; **Figure 3C**), though all associations remained statistically significant. Heterogeneity (I^2) was >99% in all models. Fixed-effects models showed weaker associations (**Data Supplement S8**).

Association between anthropometric indices and CVD

The analyses of non-fatal and fatal CVD included one cohort, four cross-sectional and four case-control studies. Four studies concluded that there was a stronger association of high BMI than of high WC and WHR with risk of CVD and CVD mortality.[25,42,43,47] The remaining five studies concluded that measures of central adiposity, namely WC and/or WHR, showed stronger associations with CVD and CVD mortality than BMI.[44–46,51,52] The meta-analysis included 30,516 cases for the association of BMI with CVD, 31,274 cases for that of WC with CVD, and 30,537 for that of WHR with CVD. The pooled risk of CVD for overweight versus normal-weight individuals, as determined by BMI, was 1.65 (95%CI: 1.55-1.75; **Figure 4A**), despite three of the six studies making up this estimate concluding non-statistically significant findings.[43,45,46] Associations appeared weaker, but still statistically significant, for large versus normal WC (OR: 1.48 [95%CI: 1.21-1.80]; **Figure 4B**), and were not statistically significant for large versus normal WHR (OR: 2.51 [95%CI: 0.94-6.69]; **Figure 4C**). Heterogeneity (I^2) was >75% in all models. Associations of BMI with CVD

For peer review only - http://bmjopen.bmj.com/site/about/guidelines.xhtml

and of WC with CVD appeared stronger in fixed-effects models, and statistically significant for WHR (OR: 1.50 [95%CI: 1.43-1.57]; **Data Supplement S9**).

Sensitivity analyses

Sensitivity analyses were conducted removing one study at a time and determining whether results were influenced by large studies or studies with extreme results. The results were not substantively different (**Data Supplement S10**).

Study quality assessment and publication bias

Overall, the quality of the studies included in the review was average, with a mean score of 6.5/9 for cohort studies, 5.2/9 for case-control studies, and 7/10 for cross-sectional studies (**Data Supplement S4a/S4b/S4c**). The domains in which studies lost points, depending on design, were principally sample representativeness, control for additional confounding factors, sample size, and discussion of non-response rate.

Publication bias was assessed by inspecting funnel plots for asymmetry and using Egger's test. Funnel plots of studies reporting on risk of HTN associated with all three anthropometric indices (BMI, WC, and WHR) showed significant skew to the right of the panel (**Figure 5**). There were no studies in the lower left panel of the funnel, with most studies concentrating at the tip of the funnel and to the right of it. Funnel plots of studies reporting on the risk of CVD showed similar results (**Figure 5**).

DISCUSSION

The purpose of this review was to provide an overview of current literature examining the association of different anthropometric measures with CVD and hypertension among South Asian populations. While BMI appeared marginally more strongly associated with CVD, WC appeared to be more strongly associated with higher SBP and DBP, as well as risk of hypertension. Overall, there appears to be a limited amount of literature focusing on the shape of these associations, whereby studies only examined anthropometric measures as continuous variables (which assumes linearity)

For peer review only - http://bmjopen.bmj.com/site/about/guidelines.xhtml

or as dichotomised variables, and an overall shape across the range of anthropometric measures was not assessed.

Comparison of South Asian effects and other ethnicities

It is important to ascertain which anthropometric measures are better predictors of morbidity. Regarding CVD, the evidence is inconclusive, and varies depending on factors such as sex, ethnicity, and subtype of CVD.[53] A large cross-sectional study, the International Day for the Evaluation of Abdominal Obesity (IDEA) study, looked at 168,000 participants across 63 countries and found WC to be a better predictor of CVD compared to BMI in men, but reported no significant difference between these measures in women (CVD ORs of BMI versus WC in men: 1.13 [95%CI: 1.09-1.17] versus 1.24 [95%CI: 1.19-1.28]; CVD ORs of BMI versus WC in women: 1.20 [95%CI: 1.16-1.24] versus 1.21 [95%CI: 1.17-1.25]).[54] Specifically, for South Asians, the study concluded that the risk of CVD associated with a 1-SD increase in BMI was 1.26 (95%CI: 1.17-1.35) for men and 1.26 (95%CI: 1.18-1.35) for women, which was similar to 1.27 (95%CI: 1.18-1.36) and 1.30 (95%CI: 1.21-1.39), respectively, for WC.[54] A large prospective study of 0.5 million Chinese adults reported similar associations for stroke when comparing BMI and WC, whereas a large prospective study of 0.5 million adults in the United Kingdom reported that BMI was more strongly associated with myocardial infarction than WC in women, but with equivalent associations in men.[55,56]

There are several possible explanations for these results. Studies indicating stronger associations between CVD and WC, as opposed to BMI, support the theory that increased central obesity may be linked to systemic inflammation, a direct contributor to CVD risk. Additionally, central obesity is associated with higher levels of free fatty acids, which can interfere with insulin metabolism, leading to hyperinsulinemia. This in turn promotes atherosclerosis, dyslipidaemia, and the release of pro-thrombotic factors, which are linked to CVD. However, the results were inconsistent across region and sex regarding the relative importance of general versus central adiposity for the risk of CVD,

For peer review only - http://bmjopen.bmj.com/site/about/guidelines.xhtml

and it may be that subgroup-specific mechanisms and their relationships to CVD subtypes have not been fully elucidated yet.

In our study, the pooled estimates indicated that BMI, rather than WC, was more strongly associated with risk of CVD. The modestly improved prognostic value of BMI in South Asians may reflect the combined effects of height, fat mass, and lean muscle mass that are each individually associated with cardiometabolic risk in this ethnic group, but not represented by WC measures.[57] Recent large-scale research comparing Asian ethnic groups identified lean mass to be positively associated with SBP, triglycerides, and HbA1c, which lie on the causal pathway of CVD.[57] Specifically, among Malay and Indian women, the associations of SBP, triglycerides, and HbA1c with appendicular lean mass were stronger than associations with BMI or fat mass. This suggests that future research needs to further unravel the correlates of BMI and WC in South Asians as it appears there are ethnic-specific mechanisms related to the risk of CVD. However, the results in this review comparing BMI to WC are still cautious as many of the included studies reported non-significant findings with CVD, likely due to low study power.[45,52] There is a need for larger prospective studies to directly compare anthropometric measures with measures of specific body composition (such as regional fat and lean mass depots) among South Asian populations (and their subtypes) to better understand the ethnic-specific aetiology of the risk of CVD.[58]

With regards to blood pressure, the wider literature concluded that BMI was positively associated with SBP (about 1mmHg per 1kg/m^2)[8], which is slightly stronger than the results of the present study where a mean difference of about 3 mmHg in SBP per 5kg/m^2 higher BMI was found. A large study on 0.5 million Chinese adults also concluded that BMI was more strongly associated with higher SBP than both WC and WHR.[59] However, the present study concluded that WC showed much stronger associations with blood pressure than both BMI and WHR. This is likely because South Asians, compared to other ethnicities, may manifest disproportionately larger waist circumferences for equivalent BMIs due to a greater propensity to store visceral fat.[60] Further research has concluded that fat distribution, specifically central adipose tissue,

For peer review only - http://bmjopen.bmj.com/site/about/guidelines.xhtml
For peer review only

may impact blood pressure variability over short-and long-term periods, with greater amounts of visceral fat linked to elevated but less variable blood pressure, and thus to incidence of hypertension.[61] From an epidemiological standpoint, this could explain why different associations have been observed in different ethnicities. Several studies also concluded that South Asians appear to have higher lipid and insulin levels compared with Europeans of the same WC and WHR.[62–64] Asian Indians tend to have greater visceral and total body fat, which is less evident from BMI measurements and differs from the typical Western build.[65] In turn, increased visceral fat can cause insulin resistance, dyslipidaemia, and inflammation, which may lead to metabolic disorders such as hypertension. The present study concluded that WC was more strongly associated with risk of HTN than BMI (OR WC: 1.45 [95%CI: 1.05-1.98]; OR BMI: 1.33 [95%CI: 1.18-1.51]), which is in keeping with this theory. While BMI has long been used as a general indicator of obesity, the recognition that WC may be more strongly associated with HTN, and that HTN is linked to increased morbidity, may allow for more accurate risk stratification and preventative interventions to address the burden of downstream CVD risk. This is particularly relevant to South Asian populations for whom previous research has concluded that blood pressure is strongly and positively associated with CVD mortality, but that BMI is little related to CVD mortality, despite higher BMI being a strong determinant of higher blood pressure and consequently HTN.[8]

The INTERHEART case-control study, which compared populations, concluded that in all sub-groups, but particularly among South Asians and mixed-race Africans, WHR was a better predictor of CVD than BMI.[66] In the present study, however, WHR was generally weaker than BMI and WC in associations with SBP, CVD and hypertension, and across fixed-effects models. In INTERHEART, both WC and WHR were strongly associated with the risk of MI, but unlike BMI, this relationship was unaffected by mutual adjustment, suggesting there is a degree of independence between measures of adiposity in predicting the risk of myocardial infarction, stressing the relative importance of central adiposity measures.[66] Thus, because SBP is strongly associated with CVD

For peer review only

For peer review only - http://bmjopen.bmj.com/site/about/guidelines.xhtml

risk, some adverse or protective correlate of low BMI is likely associated with CVD, particularly among South Asians.[67,68]

Finally, the shape of the associations of different anthropometric indices and CVD among South Asian populations has been scarcely analysed across the literature, with most studies only examining anthropometric measures as dichotomous variables or by calculating risks for continuous measures that assume linearity. It was therefore not possible to review results across the range in comparison to the wider literature. Given the generally flat associations concluded by the Chennai Prospective Study, a comparison of shape across the range would make for a useful analysis.

Study quality assessment

The quality of the studies included in this review was average, with most studies scoring five or six out of nine or ten possible total points on the Newcastle-Ottawa scale. Several studies recorded CVD outcomes based on self-report or verbal autopsy, largely due to local lack of national registries or reported death certification. Eight studies investigating CVD endpoints such as stroke, myocardial infarction, or death from CVD were case-control or cross-sectional in design and therefore potentially limited by reverse causality. While the included studies are limited in some respects, the populations within each study were homogenous in terms of age, sex ratio, and education, indicating potential internal validity.

Strengths and limitations of this review

This meta-analysis has several strengths. The overall large size enables assessment of the relationship between different anthropometric measures and CVD, with an appreciation of all relevant literature on this topic. Despite significant heterogeneity, sensitivity analyses excluding large studies contributing the most to the models showed that associations remained largely unchanged.

Due to the nature of the studies and the extent of statistical heterogeneity observed, this systematic review also has limitations. Firstly, while the review aims to assess risk

For peer review only - http://bmjopen.bmj.com/site/about/guidelines.xhtml

across South Asia, most studies were conducted in India, thus limiting generalisability of findings to the rest of the sub-continent. Additionally, funnel plots of studies reporting on risk of HTN or CVD associated with all three anthropometric indices (BMI, WC, and WHR) showed possible publication bias (**Figure 5**). This may have impacted the ability to accurately synthesize the direction and strength of associations. Nevertheless, this reflects the current body of evidence, and highlights an important gap in the literature. Secondly, it may have been beneficial to exclude cross-sectional and case-control studies in examining the association of anthropometric measures with CVD endpoints such as MI and CVD to minimize reverse causality. However, a large proportion of the identified evidence was based on these study designs and excluding such studies would have skewed findings, thus biasing results. Thirdly, despite ensuring the most fully adjusted models were used for the meta-analyses, we cannot rule out the possibility that observed associations are confounded by unmeasured factors such as physical activity and diet, including dietary salt consumption. Few studies adjusted for physical activity– a known confounder of cardiovascular health. The majority of studies also failed to control for other important confounders, for example menopause status among females or dietary salt consumption, which are related independently to both anthropometric indices and CVD.[26,51]

CONCLUSION

From a clinical point of view, health practitioners should be made aware of ethnic variations in CVD risk and how these relate to different measures of anthropometry. There is scope for measures such as WC and WHR to become routinely included in health records, alongside BMI, if these are truly deemed stronger CVD predictors. However, these measures would need to be robustly measured, which is not always straightforward in busy clinical environments and one of the reasons why BMI is more widely employed. Development of a point-of-care CVD risk score based on these measures may also prove an effective population-level prevention strategy.

Ultimately, large prospective studies among South Asian populations are required to clarify whether measures of central adiposity may be better predictors of CVD. Ideally,

For peer review only - http://bmjopen.bmj.com/site/about/guidelines.xhtml

these studies would directly compare different measures of adiposity with risk over time. There is also potential value in imaging-based studies to characterize the distribution of adipose tissue more reliably. More considered cut-offs of different body composition measures, which consider location of fat deposition, may be needed, as well as an assessment of the shape of the relationship across the full range. Given the high prevalence of CVD globally, and the rapidly increasing prevalence among South Asian populations, this may have important implications from a public health perspective with potential to achieve better-targeted CVD primary prevention.

For peer review only

For peer review only - http://bmjopen.bmj.com/site/about/guidelines.xhtml

FIGURE LEGENDS

Figure 1. PRISMA flowchart describing the systematic literature search and study selection.

Figure 2. Mean change in systolic blood pressure (SBP) per 5kg/m^2 higher body mass index (BMI, A), 13cm larger waist circumference (WC, B) and 0.1-unit larger waist-to-hip ratio (WHR, C). Random effects models were applied to four studies reporting associations of SBP and BMI, four studies reporting on SBP and WC, and two studies reporting on SBP and WHR. The total number of participants was 8,143 for panels A and B, and 7,781 for panel C. *Abbreviations: 95% confidence interval (95%CI).*

Figure 3. Odds ratio (OR) of hypertension (HTN) per 5kg/m^2 higher body mass index (BMI, A), 13cm larger waist circumference (WC, B) and 0.1-units larger waist-to-hip ratio (WHR, C). Random effects models were applied to 13 studies looking at BMI, seven studies looking at WC, and seven studies looking at WHR. The total number of participants was 11,616 for panel A, 4,899 for panel B, and 4,456 for panel C. *Abbreviations: 95% confidence interval (95%CI).*

Figure 4. Odds ratios (OR) of cardiovascular disease (CVD) for overweight (>25kg/m^2) vs. normal (18.5-24.9 kg/m^2) body mass index (BMI, panel A), large ((≥80cm in females, ≥90cm in males) vs. normal waist circumference (WC, panel B), and large (≥0.8-units in females, ≥1.0-units in males) vs. normal waist-to-hip ratio (WHR, panel C). Random effects models were applied to six studies looking at BMI, seven studies looking at WC, and six studies looking at WHR. *Abbreviations: 95% confidence interval (95%CI).*

Figure 5. Funnel plots of studies on BMI-HTN (A), WC-HTN (B), WHR-HTN (C) and BMI-CVD (D), WC-CVD (E) and WHR-CVD (F). Egger's test was b=2.27 (p=0.0025) for BMI and HTN, b=2.96 (p<0.001) for WC and HTN, and b=4.58 (p=0.003) for WHR and HTN. With regards to the CVD measures, Egger's test was b=0.43 (p=0.659) for BMI and CVD, b=0.42 (p=0.614) for WC and CVD and b=4.32 (p=0.0031) for WHR and CVD. *Abbreviations: body mass index, kg/m^2 (BMI); hypertension (HTN); waist circumference, cm (WC); waist-to-hip ratio (WHR); odds ratio (OR); 95% confidence interval (95%CI).*

For peer review only - http://bmjopen.bmj.com/site/about/guidelines.xhtml

Acknowledgements: None.

Funding: The study was funded by core support from the UK Medical Research Council (MRC), British Heart Foundation, and Cancer Research UK to the Clinical Trial Service Unit and the MRC Population Health Research Unit, both now in the Nuffield Department of Population Health, University of Oxford (Oxford, UK).

Competing interests: None declared.

Patient and public involvement: Patients and/or the public were not involved in the design, conduct, reporting, or dissemination plans of this research.

Patient consent for publication: Not applicable.

Data availability: Individual data should be requested from the original or parent study investigators of the studies included in this review.

Ethical approval: Ethical approval was not required for the present systematic review and meta-analysis. It is assumed that all published research on which analyses were based will have fulfilled respective ethical standards at the time of publication.

Author contributions: FR, JLC, and FB contributed to the design of the study and the statistical analysis plan. FR and ASO conducted the analysis. All authors (FR, ASO, BB, FB, JLC) contributed to the interpretation of the analysis and the presentation of results. FR was responsible for drafting the manuscript. All authors contributed to reviewing and editing the manuscript, and all authors have agreed to the final version of the manuscript.

For peer review only

For peer review only - http://bmjopen.bmj.com/site/about/guidelines.xhtml

REFERENCES

1 Hubert HB, Mcnamara PM, Castelli WP. Obesity as an independent risk factor for cardiovascular disease: a 26-year follow-up of participants in the Framingham Heart Study. *Circulation* 1983;**67**:10. doi:10.1161/01.cir.67.5.968

2 Manson J, Colditz G, Stamper M, *et al.* A Prospective Study of Obesity and Risk of Coronary Heart Disease in Women. *The New England Journal of Medicine* 1990;**322**:882–9. doi:10.1056/NEJM199003293221303

3 Dorn J, Schisterman E, Winkelstein W, *et al.* Body Mass Index and Mortality in a General Population Sample of Men and Women: The Buffalo Health Study. *Am J Epidemiol* 1997;**128**:919–31. doi:10.1093/oxfordjournals.aje.a009218

4 Di Angelantonio E, Bhupathiraju SN, Wormser D, *et al.* Body-mass index and all-cause mortality: individual-participant-data meta-analysis of 239 prospective studies in four continents. *The Lancet* 2016;**388**:776–86. doi:10.1016/S0140-6736(16)30175-1

5 Fan J, Song Y, Chen Y, *et al.* Combined effect of obesity and cardio-metabolic abnormality on the risk of cardiovascular disease: A meta-analysis of prospective cohort studies. *International Journal of Cardiology* 2013;**168**:4761–8. doi:10.1016/j.ijcard.2013.07.230

6 McGee DL, The Diverse Population Collaboration. Body mass index and mortality: a meta-analysis based on person-level data from twenty-six observational studies. *Annals of Epidemiology* 2005;**15**:87–97. doi:10.1016/j.annepidem.2004.05.012

7 van Dijk SB, Takken T, Prinsen EC, *et al.* Different anthropometric adiposity measures and their association with cardiovascular disease risk factors: a meta-analysis. *Neth Heart J* 2012;**20**:208–18. doi:10.1007/s12471-011-0237-7

8 Gajalakshmi V, Lacey B, Kanimozhi V, *et al.* Body-mass index, blood pressure, and cause-specific mortality in India: a prospective cohort study of 500 810 adults. *Lancet Glob Health* 2018;**6**:e787–94. doi:10.1016/S2214-109X(18)30267-5

9 Yusuf S, Hawken S, Ôunpuu S, *et al.* Obesity and the risk of myocardial infarction in 27 000 participants from 52 countries: a case-control study. *The Lancet* 2005;**366**:1640–9. doi:10.1016/S0140-6736(05)67663-5

10 Chen Y, Copeland WK, Vedanthan R, *et al.* Association between body mass index and cardiovascular disease mortality in east Asians and south Asians: pooled analysis of prospective data from the Asia Cohort Consortium. *BMJ* 2013;**347**. doi:10.1136/bmj.f5446

11 Shah A, Kanaya AM. Diabetes and Associated Complications in the South Asian Population. *Curr Cardiol Rep* 2014;**16**:476. doi:10.1007/s11886-014-0476-5

For peer review only - http://bmjopen.bmj.com/site/about/guidelines.xhtml

12 Blüher M, Laufs U. New concepts for body shape-related cardiovascular risk: role of fat distribution and adipose tissue function. *European Heart Journal* 2019;**40**:2856–8. doi:10.1093/eurheartj/ehz411

13 Neeland IJ, Poirier P, Després J-P. Cardiovascular and Metabolic Heterogeneity of Obesity: Clinical Challenges and Implications for Management. *Circulation* 2018;**137**:1391–406. doi:10.1161/CIRCULATIONAHA.117.029617

14 Huxley R, Mendis S, Zheleznyakov E, *et al.* Body mass index, waist circumference and waist:hip ratio as predictors of cardiovascular risk—a review of the literature. *Eur J Clin Nutr* 2010;**64**:16–22. doi:10.1038/ejcn.2009.68

15 Page M, McKenzie J, Bossuyt P, *et al.* The PRISMA 2020 statement: an updated guideline for reporting systematic reviews. *BMJ* Published Online First: 2021. doi:10.1136/bmj.n71

16 Cochrane Community. Covidence. The Future of Evidence Synthesis in Cochrane. 2022.

17 Wells G, Shea B, O'Connell D, *et al.* The Newcastle-Ottawa Scale (NOS) for Assessing the Quality of Nonrandomised Studies in Meta-analyses. Ottawa Hospital Research Institute. https://www.ohri.ca/programs/clinical_epidemiology/oxford.asp

18 Modesti P, Reboldi G, Cappuccio F, *et al.* Panethnic differences in blood pressure in Europe: a systematic review and meta-analysis. *PLoS ONE* 2016;**11**. doi:10.1371/journal.pone.0147601

19 Taing K, Farkouh M, Moineddin R, *et al.* Age and sex-specific associations of anthropometric measures of adiposity with blood pressure and hypertension in India: a cross-sectional study. *BMC Cardiovasc Disord* 2016;**16**. doi:10.1186/s12872-016-0424-y

20 Green J, Cairns B, Casabonne D, *et al.* Height and cancer incidence in the Million Women Study: prospective cohort, and meta-analysis of prospective studies of height and total cancer risk. *The Lancet Oncology* 2011;**12**:785–94. doi:10.1016/S1470-2045(11)70154-1

21 Orsini N. Weighted mixed-effects dose–response models for tables of correlated contrasts. *The Stata Journal* 2021;**21**:320–47. doi:10.1177/1536867X211025798

22 Shim S, Shin I, Yoon B, *et al.* Dose-response meta-analysis using STATA Software. *J Health Info Stat* 2016;**41**:351–8. doi:10.21032/jhis.2016.41.3.351

23 Papier K, Knuppel A, Syam N, *et al.* Meat consumption and risk of ischemic heart disease : A systematic review and meta-analysis. *Critical Reviews in Food Science and Nutrition* 2021;**0**:1–12. doi:10.1080/10408398.2021.1949575

For peer review only

For peer review only - http://bmjopen.bmj.com/site/about/guidelines.xhtml

24 Deshpande-Joshi S, Rao S. Differential risk of hypertension among lean and non-lean rural subjects in relation to decadal changes in anthropometry. *J Am Coll Nutr* 2018;**37**:380–6. doi:10.1080/07315724.2017.1405750

25 Bramhankar M, Pandey M, Rana G, *et al.* An assessment of anthropometric indices and its association with NCDs among the older adults of India: evidence from LASI Wave-1. *BMC Public Health* 2021;**21**:1357. doi:10.1186/s12889-021-11421-4

26 Agrawal P, Gupta K, Aboyans V, *et al.* Women's health in India: the role of body mass index. *Health Care Women Int* 2014;**36**:320–41. doi:10.1080/07399332.2014.892110

27 Bose K, Ghosh A, Roy S, *et al.* The relationship of age, body mass index and waist circumference with blood pressure in Bengalee Hindu male jute mill workers of Belur, West Bengal, India. *Anthropology Anz* 2005;**63**:205–12. doi:PMID: 15962571

28 Dhall M, Devi K, Nilupher A, *et al.* Hypertension and its correlate with general and central adiposity: A study among urban population of Delhi. *Diabetes Metab Syndr* 2018;**12**:881–4. doi:10.1016/j.dsx.2018.05.008

29 Ganguli D, Das N, Saha I, *et al.* Risk factors for hypertension in a population-based sample of postmenopausal women in Kolkata, West Bengal, India. *Asia Pac J Public Health* 2013;**25**:388–97. doi:10.1177/1010539511420703

30 Ghosh J, Bandyopadhyay A. Comparative evaluation of obesity measures: relationship with blood pressure and hypertension. *Singapore Med J* 2007;**48**:232–5. doi:PMID: 17342293

31 Hazarika N, Narain K, Biswas D, *et al.* Hypertension in the native rural population of Assam. *Natl Med J India* 2004;**17**:300–4. doi:PMID: 15736549

32 Kaur P, Rao S, Radhakrishnan E, *et al.* Prevalence, awareness, treatment, control and risk factors for hypertension in a rural population in South India. *Int J Public Health* 2012;**57**:87–94. doi:10.1007/s00038-011-0303-3

33 Kaur P, Radhakrishnan E, Sankarasubbaiyan S, *et al.* A Comparison of Anthropometric Indices for Predicting Hypertension and Type 2 Siabetes in a Male Industrial Population of Chennai, South India. *Ethnicity and Disease* 2008;**18**:31–26.

34 Meshram II, Arlappa N, Balkrishna N, *et al.* Prevalence of hypertension, its correlates and awareness among adult tribal population of Kerala state, India. *J Postgrad Med* 2012;**58**:255–61. doi:10.4103/0022-3859.105444

35 Meshram II, Vishnu Vardhana Rao M, Sudershan Rao V, *et al.* Regional variation in the prevalence of overweight/obesity, hypertension and diabetes and their correlates among the adult rural population in India. *Br J Nutr* 2016;**115**:1265–72. doi:10.1017/S0007114516000039

For peer review only - http://bmjopen.bmj.com/site/about/guidelines.xhtml

36 Shriraam V, Mahadevan S, Arumugam P. Prevalence and risk factors of diabetes, hypertension and other non-communicable diseases in a tribal population in South India. *Indian J Endocr Metab* 2021;**25**:313. doi:10.4103/ijem.ijem_298_21

37 Singh R, Mukherjee M, Kumar R, *et al.* Study of risk factors of coronary heart disease in urban slums of Patna. *Nepal J Epidemiology* 2012;**2**:205–12. doi:10.3126/nje.v2i3.6902

38 Tselha N, Shimrah C, Kulshreshtha M, *et al.* Association between hypertension and adiposity indicators: A study among the Muslim population of Uttar Pradesh. *Diabetes Metab Syndr* 2019;**13**:2335–8. doi:10.1016/j.dsx.2019.05.016

39 Venkatramana P, Reddy P. Association of overall and abdominal obesity with coronary heart disease risk factors: comparison between urban and rural Indian men. *Asia Pac J Clin Nutr* 2002;**11**:66–71. doi:10.1046/j.1440-6047.2002.00250.x

40 Vikram N, Latifi A, Misra A, *et al.* Waist-to-height ratio compared to standard obesity measures as predictor of cardiometabolic risk factors in Asian Indians in North India. *Metab Syndr Relat Disord* 2016;**14**:492–9. doi:10.1089/met.2016.0041

41 Yadav S, Boddula R, Genitta G, *et al.* Prevalence & risk factors of pre-hypertension & hypertension in an affluent north Indian population. *Indian J Med Res* 2008;:712–20. doi:PMID: 19246794

42 Banerjee S, Kumar P, Srivastava S, *et al.* Association of anthropometric measures of obesity and physical Activity with ardiovascular diseases among older adults: evidence from a cross-sectional survey, 2017–18. *PLoS ONE* 2021;**16**. doi:10.1371/journal.pone.0260148

43 Bodkhe S, Jajoo S, Jajoo U, *et al.* Epidemiology of confirmed coronary heart disease among population older than 60 years in rural central India—A community-based cross-sectional study. *Indian Heart Journal* 2019;**71**:39–44. doi:10.1016/j.ihj.2019.01.002

44 Latheef SAA, Subramanyam G, Reddy BM. Utility of anthropometric traits and indices in predicting the risk of coronary artery disease in the adult men of southern Andhra Pradesh. *Indian Heart J* 2018;**70**:S133–9. doi:10.1016/j.ihj.2018.07.016

45 Patil S, Joshi R, Gupta G, *et al.* Risk factors for acute myocardial infarction in a rural population of central India: A hospital-based case–control study. *Nat Med J India* 2004;**17**. doi:PMID: 15372760

46 Prabhakar S, Suravarapu S, Mathai D, *et al.* Risk factors for stroke in rural population of Telangana State of India, an unmatched case-control study. *J Neurosci Rural Pract* 2020;**11**:448–53. doi:10.1055/s-0040-1713291

For peer review only

For peer review only - http://bmjopen.bmj.com/site/about/guidelines.xhtml

47 Shah M, Mazumdar V, Patel S, *et al.* A case control study of risk factors of coronary heart disease among patients admitted at tertiary hospital in western India. *Australasian Medical Journal* 2017;**10**:381–8. doi:10.21767/AMJ.2017.2860

48 Islam FMA, Bhuiyan A, Chakrabarti R, *et al.* Undiagnosed hypertension in a rural district in Bangladesh: The Bangladesh Population-based Diabetes and Eye Study (BPDES). *J Hum Hypertens* 2016;**30**:252–9. doi:10.1038/jhh.2015.65

49 Khanam R, Ahmed S, Rahman S, *et al.* Prevalence and factors associated with hypertension among adults in rural Sylhet district of Bangladesh: a cross-sectional study. *BMJ Open* 2019;**9**. doi:10.1136/bmjopen-2018-026722

50 Simmons S, Hagan Jr. J, Schack T. The influence of anthropometric indices and intermediary determinants of hypertension in Bangladesh. *IJERPH* 2021;**18**. doi:10.3390/ijerph18115646

51 Cameron A, Magliano D, Shaw J, *et al.* The influence of hip circumference on the relationship between abdominal obesity and mortality. *Intl J of Epi* 2012;**41**:484–94. doi:10.1093/ije/dyr198

52 Nishtar S, Wierzbicki A, Lumb P, *et al.* Waist-hip ratio and low HDL predict the risk of coronary artery disease in Pakistanis. *Current Medical Research and Opinion* 2008;**20**:55–62. doi:10.1185/030079903125002595

53 Goh LGH, Dhaliwal SS, Welborn TA, *et al.* Anthropometric measurements of general and central obesity and the prediction of cardiovascular disease risk in women: a cross-sectional study. *BMJ Open* 2014;**4**. doi:10.1136/bmjopen-2013-004138

54 Balkau B, Deanfield J, Després J, *et al.* International Day for the Evaluation of Abdominal Obesity (IDEA): A study of waist circumference, cardiovascular disease, and diabetes mellitus in 168,000 primary care patients in 63 countries. *Circulation* 2007;**116**:1942–51. doi:10.1161/CIRCULATIONAHA.106.676379

55 Chen Z, Iona A, Parish S, *et al.* Adiposity and risk of ischaemic and haemorrhagic stroke in 0.5 million Chinese men and women: a prospective cohort study. *The Lancet Global Health* 2018;**6**:e630–40. doi:10.1016/S2214-109X(18)30216-X

56 Peters S, Bots S, Woodward M. Sex Differences in the Association Between Measures of General and Central Adiposity and the Risk of Myocardial Infarction: Results From the UK Biobank. *JAHA* 2018;**7**. doi:10.1161/JAHA.117.008507

57 Wells J. Commentary: The paradox of body mass index in obesity assessment: not a good index of adiposity, but not a bad index of cardio-metabolic risk. *Int J Epidemiol* 2014;**43**:672–4. doi:10.1093/ije/dyu060

58 Li X, Qi L. Gene–Environment Interactions on Body Fat Distribution. *IJMS* 2019;**20**:3690. doi:10.3390/ijms20153690

For peer review only - http://bmjopen.bmj.com/site/about/guidelines.xhtml

59 Chen Z, Smith M, Du H, *et al.* Blood pressure in relation to general and central adiposity among 500 000 adult Chinese men and women. *Int J Epidemiol* 2015;**44**:1305–19. doi:10.1093/ije/dyv012

60 Carter J, Abdullah N, Bragg F, *et al.* Body composition and risk factors for cardiovascular disease in global multi-ethnic populations. *Int J Obes* 2023;**47**:855–64. doi:10.1038/s41366-023-01339-9

61 Levelt E, Pavlides M, Banerjee R, *et al.* Ectopic and Visceral Fat Deposition in Lean and Obese Patients With Type 2 Diabetes. *J Am Coll Cardiol* 2016;**68**:53–63. doi:10.1016/j.jacc.2016.03.597

62 Chandalia M, Abate N, Garg A, *et al.* Relationship between Generalized and Upper Body Obesity to Insulin Resistance in Asian Indian Men. *The Journal of Clincial Endocrineology and Metabolism* 1999;**84**:7.

63 Lean M, Han T, Bush H, *et al.* Ethnic differences in anthropometric and lifestyle measures related to coronary heart disease risk between South Asian, Italian and general-population British women living in the west of Scotland. *Int J Obes Relat Metab Disord* 2001;**25**:1800–5. doi:10.1038/sj.ijo.0801823

64 Rush E, Goedecke J, Jennings C, *et al.* BMI, fat and muscle differences in urban women of five ethnicities from two countries. *Int J Obes* 2007;**31**:1232–9. doi:10.1038/sj.ijo.0803576

65 Banerji MA, Faridi N, Atluri R, *et al.* Body Composition, Visceral Fat, Leptin, and Insulin Resistance in Asian Indian Men. *Journal of Clinical Endocrineology and Metabolism* 1999;**84**:8. doi:10.1210/jcem.84.1.5371

66 Yusuf S, Hawken S, McQueen M, *et al.* Effect of potentially modifiable risk factors associated with myocardial infarction in 52 countries (the INTERHEART study): case-control study. *The Lancet* 2004;**364**:937–52. doi:10.1016/S0140-6736(04)17018-9

67 Deurenberg P, Deurenberg-Yap M, Guricci S. Asians are different from Caucasians and from each other in their body mass index/body fat per cent relationship. *Obesity Reviews* 2002;**3**:141–6. doi:10.1046/j.1467-789X.2002.00065.x

68 Lear SA, Humphries KH, Kohli S, *et al.* The Use of BMI and Waist Circumference as Surrogates of Body Fat Differs by Ethnicity**. *Obesity* 2007;**15**:2817–24. doi:10.1038/oby.2007.334

For peer review only - http://bmjopen.bmj.com/site/about/guidelines.xhtml

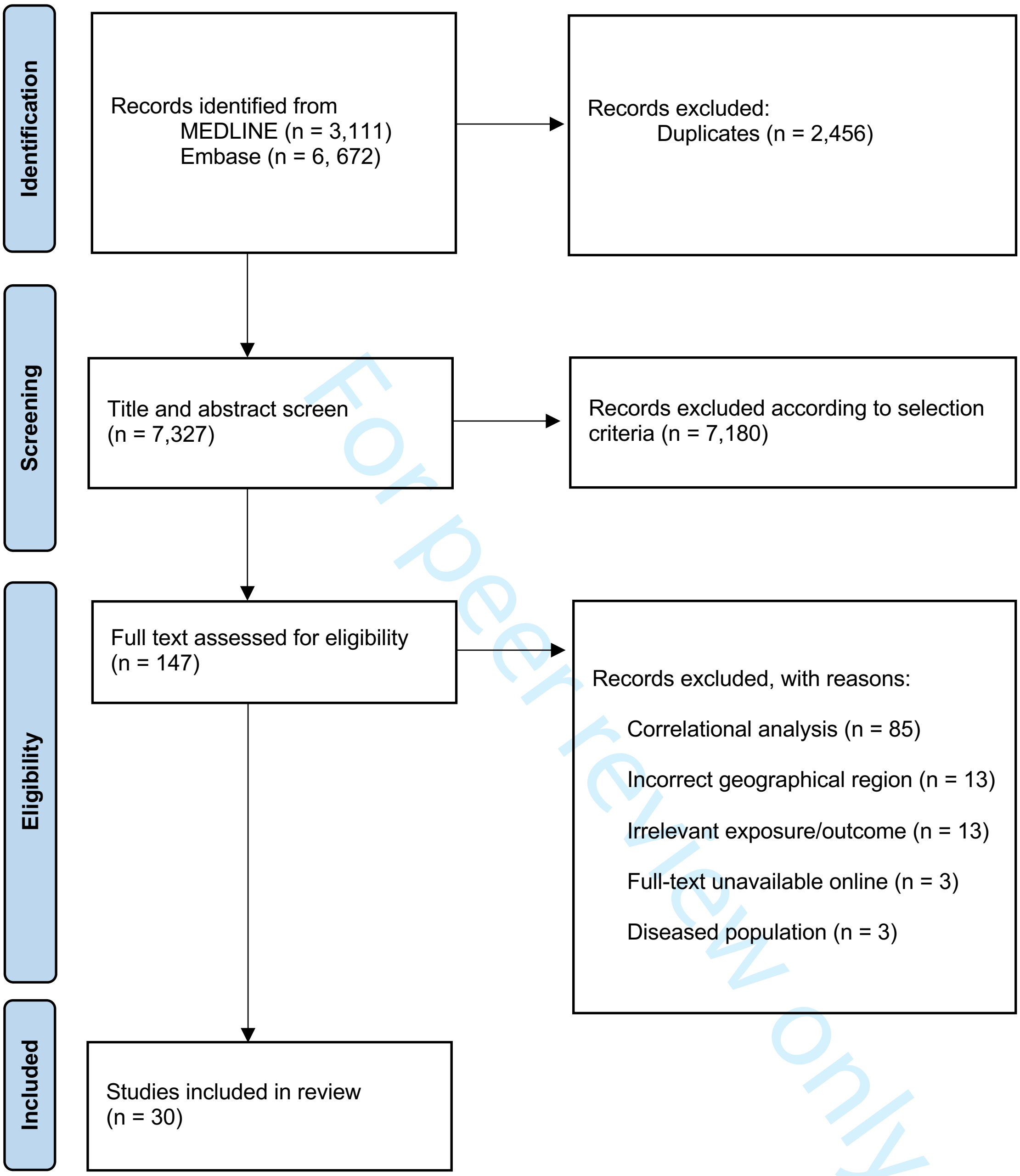

For peer review only

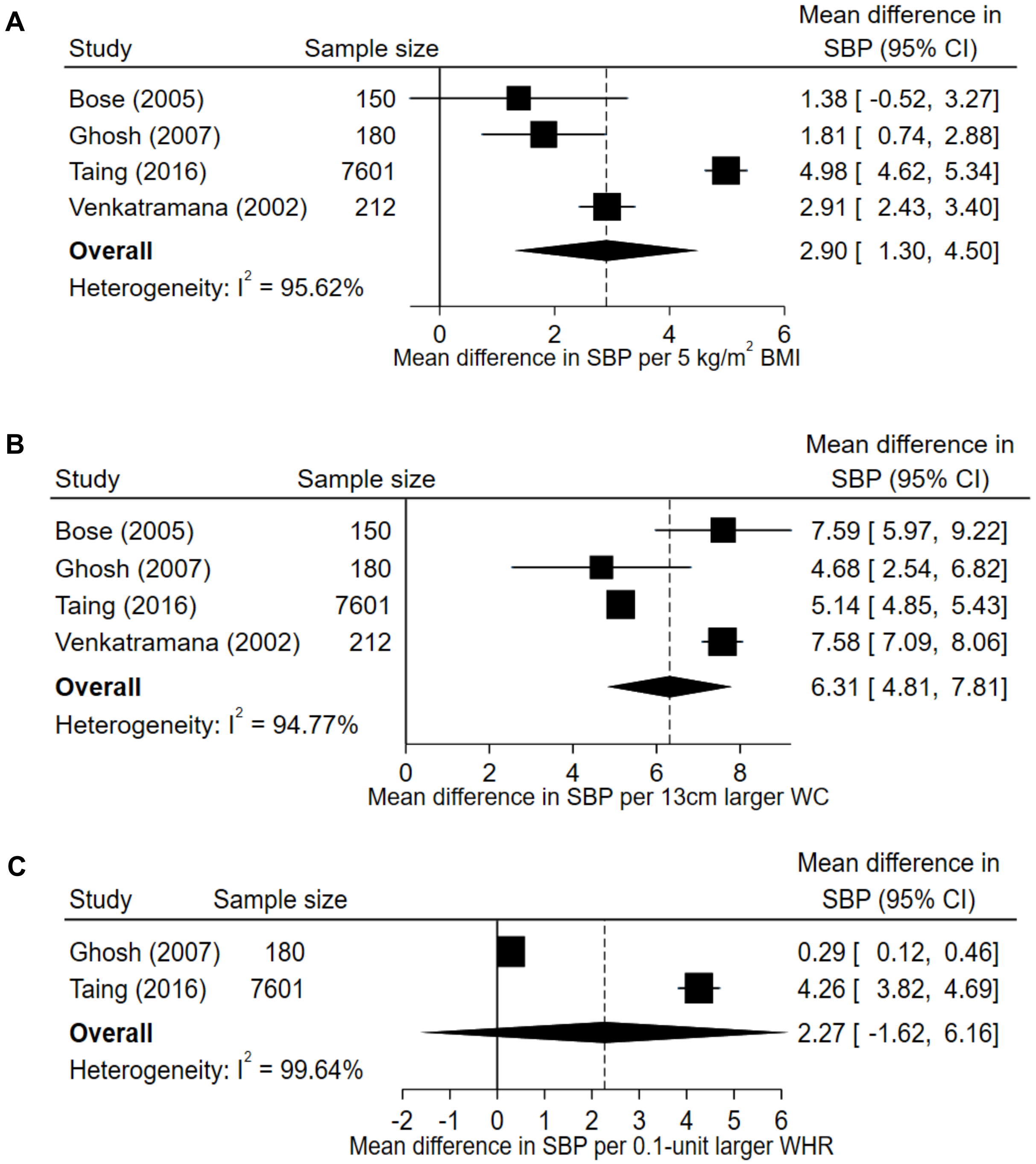
A
Study
Sample size
Mean difference in SBP (95% CI)
Bose (2005) 150 1.38 [-0.52, 3.27]
Ghosh (2007) 180 1.81 [0.74, 2.88]
Taing (2016) 7601 4.98 [4.62, 5.34]
Venkatramana (2002) 212 2.91 [2.43, 3.40]
Overall 2.90 [1.30, 4.50]
Heterogeneity: I² = 95.62%
0 2 4 6
Mean difference in SBP per 5 kg/m² BMI
B
Study
Sample size
Mean difference in SBP (95% CI)
Bose (2005) 150 7.59 [5.97, 9.22]
Ghosh (2007) 180 4.68 [2.54, 6.82]
Taing (2016) 7601 5.14 [4.85, 5.43]
Venkatramana (2002) 212 7.58 [7.09, 8.06]
Overall 6.31 [4.81, 7.81]
Heterogeneity: I² = 94.77%
0 2 4 6 8
Mean difference in SBP per 13cm larger WC
C
Study
Sample size
Mean difference in SBP (95% CI)
Ghosh (2007) 180 0.29 [0.12, 0.46]
Taing (2016) 7601 4.26 [3.82, 4.69]
Overall 2.27 [-1.62, 6.16]
Heterogeneity: I² = 99.64%
-2 -1 0 1 2 3 4 5 6
Mean difference in SBP per 0.1-unit larger WHR

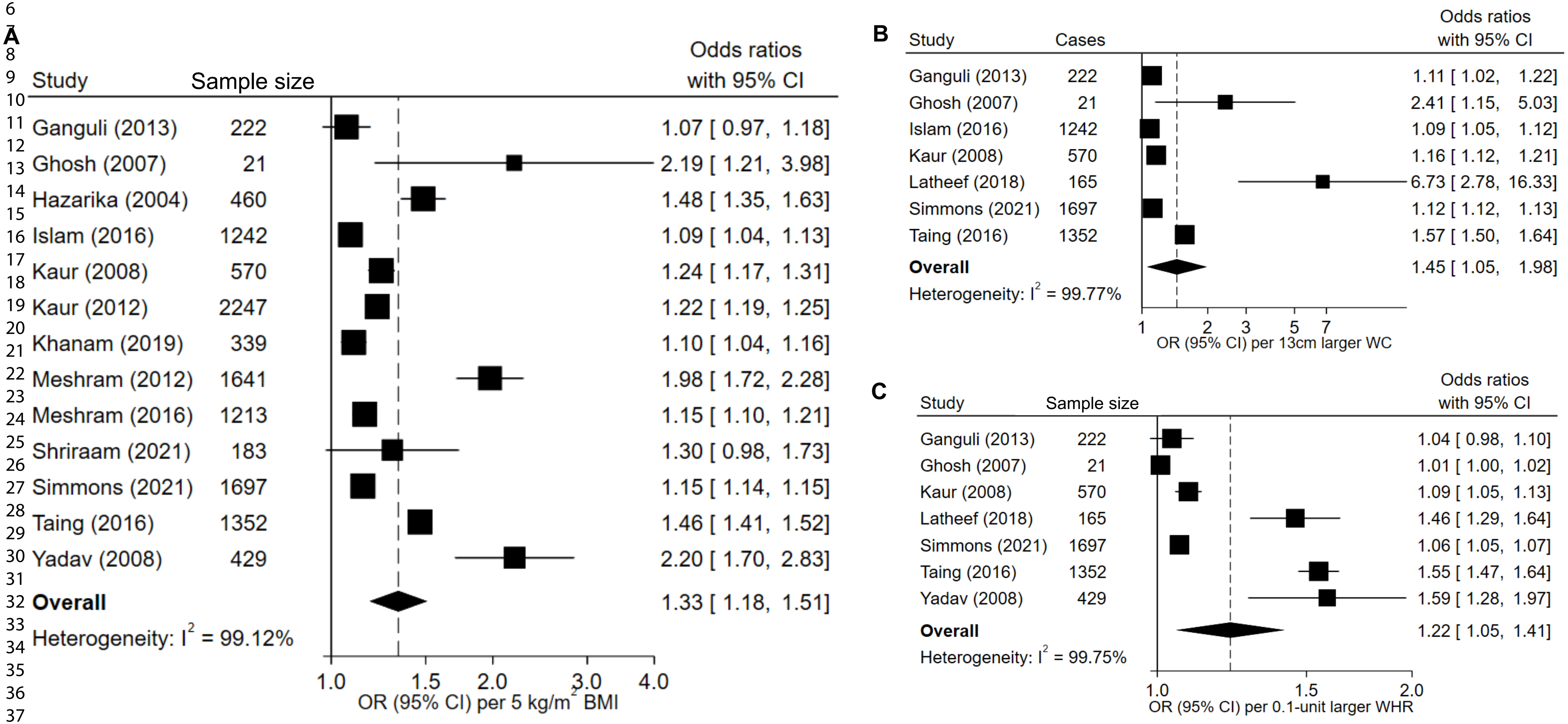
A
Study
Sample size
Odds ratios with 95% CI
Ganguli (2013) 222 1.07 [0.97, 1.18]
Ghosh (2007) 21 2.19 [1.21, 3.98]
Hazarika (2004) 460 1.48 [1.35, 1.63]
Islam (2016) 1242 1.09 [1.04, 1.13]
Kaur (2008) 570 1.24 [1.17, 1.31]
Kaur (2012) 2247 1.22 [1.19, 1.25]
Khanam (2019) 339 1.10 [1.04, 1.16]
Meshram (2012) 1641 1.98 [1.72, 2.28]
Meshram (2016) 1213 1.15 [1.10, 1.21]
Shriraam (2021) 183 1.30 [0.98, 1.73]
Simmons (2021) 1697 1.15 [1.14, 1.15]
Taing (2016) 1352 1.46 [1.41, 1.52]
Yadav (2008) 429 2.20 [1.70, 2.83]
Overall 1.33 [1.18, 1.51]
Heterogeneity: I2 = 99.12%
1.0 1.5 2.0 3.0 4.0
OR (95% CI) per 5 kg/m2 BMI
B
Study
Cases
Odds ratios with 95% CI
Ganguli (2013) 222 1.11 [1.02, 1.22]
Ghosh (2007) 21 2.41 [1.15, 5.03]
Islam (2016) 1242 1.09 [1.05, 1.12]
Kaur (2008) 570 1.16 [1.12, 1.21]
Latheef (2018) 165 6.73 [2.78, 16.33]
Simmons (2021) 1697 1.12 [1.12, 1.13]
Taing (2016) 1352 1.57 [1.50, 1.64]
Overall 1.45 [1.05, 1.98]
Heterogeneity: I2 = 99.77%
1 2 3 5 7
OR (95% CI) per 13cm larger WC
C
Study
Sample size
Odds ratios with 95% CI
Ganguli (2013) 222 1.04 [0.98, 1.10]
Ghosh (2007) 21 1.01 [1.00, 1.02]
Kaur (2008) 570 1.09 [1.05, 1.13]
Latheef (2018) 165 1.46 [1.29, 1.64]
Simmons (2021) 1697 1.06 [1.05, 1.07]
Taing (2016) 1352 1.55 [1.47, 1.64]
Yadav (2008) 429 1.59 [1.28, 1.97]
Overall 1.22 [1.05, 1.41]
Heterogeneity: I2 = 99.75%
1.0 1.5 2.0
OR (95% CI) per 0.1-unit larger WHR

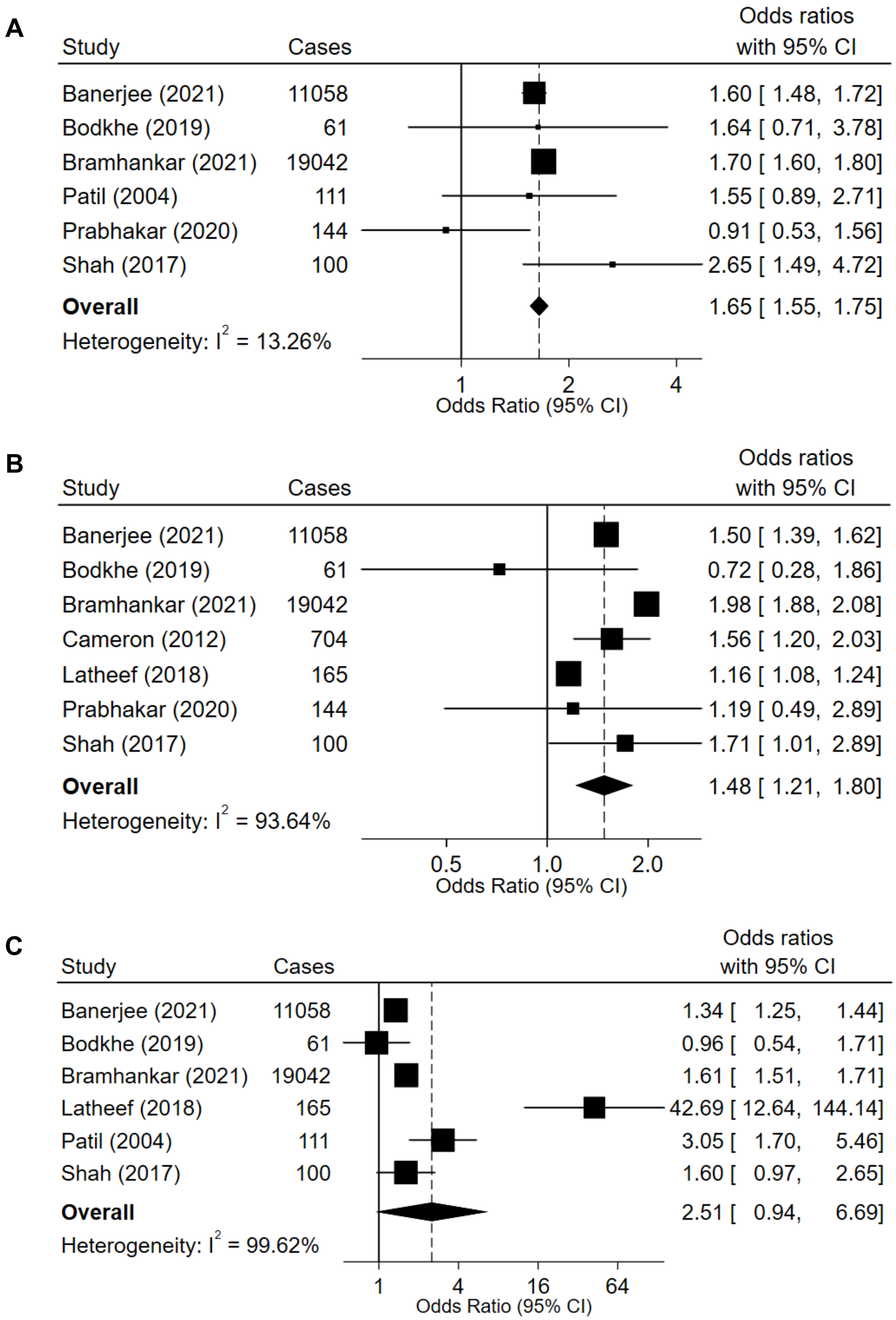
A
Study
Cases
Odds ratios
with 95% CI
Banerjee (2021) 11058 1.60 [1.48, 1.72]
Bodkhe (2019) 61 1.64 [0.71, 3.78]
Bramhankar (2021) 19042 1.70 [1.60, 1.80]
Patil (2004) 111 1.55 [0.89, 2.71]
Prabhakar (2020) 144 0.91 [0.53, 1.56]
Shah (2017) 100 2.65 [1.49, 4.72]
Overall 1.65 [1.55, 1.75]
Heterogeneity: I2 = 13.26%
1
2
4
Odds Ratio (95% CI)
B
Study
Cases
Odds ratios
with 95% CI
Banerjee (2021) 11058 1.50 [1.39, 1.62]
Bodkhe (2019) 61 0.72 [0.28, 1.86]
Bramhankar (2021) 19042 1.98 [1.88, 2.08]
Cameron (2012) 704 1.56 [1.20, 2.03]
Latheef (2018) 165 1.16 [1.08, 1.24]
Prabhakar (2020) 144 1.19 [0.49, 2.89]
Shah (2017) 100 1.71 [1.01, 2.89]
Overall 1.48 [1.21, 1.80]
Heterogeneity: I2 = 93.64%
0.5
1.0
2.0
Odds Ratio (95% CI)
C
Study
Cases
Odds ratios
with 95% CI
Banerjee (2021) 11058 1.34 [1.25, 1.44]
Bodkhe (2019) 61 0.96 [0.54, 1.71]
Bramhankar (2021) 19042 1.61 [1.51, 1.71]
Latheef (2018) 165 42.69 [12.64, 144.14]
Patil (2004) 111 3.05 [1.70, 5.46]
Shah (2017) 100 1.60 [0.97, 2.65]
Overall 2.51 [0.94, 6.69]
Heterogeneity: I2 = 99.62%
1
4
16
64
Odds Ratio (95% CI)

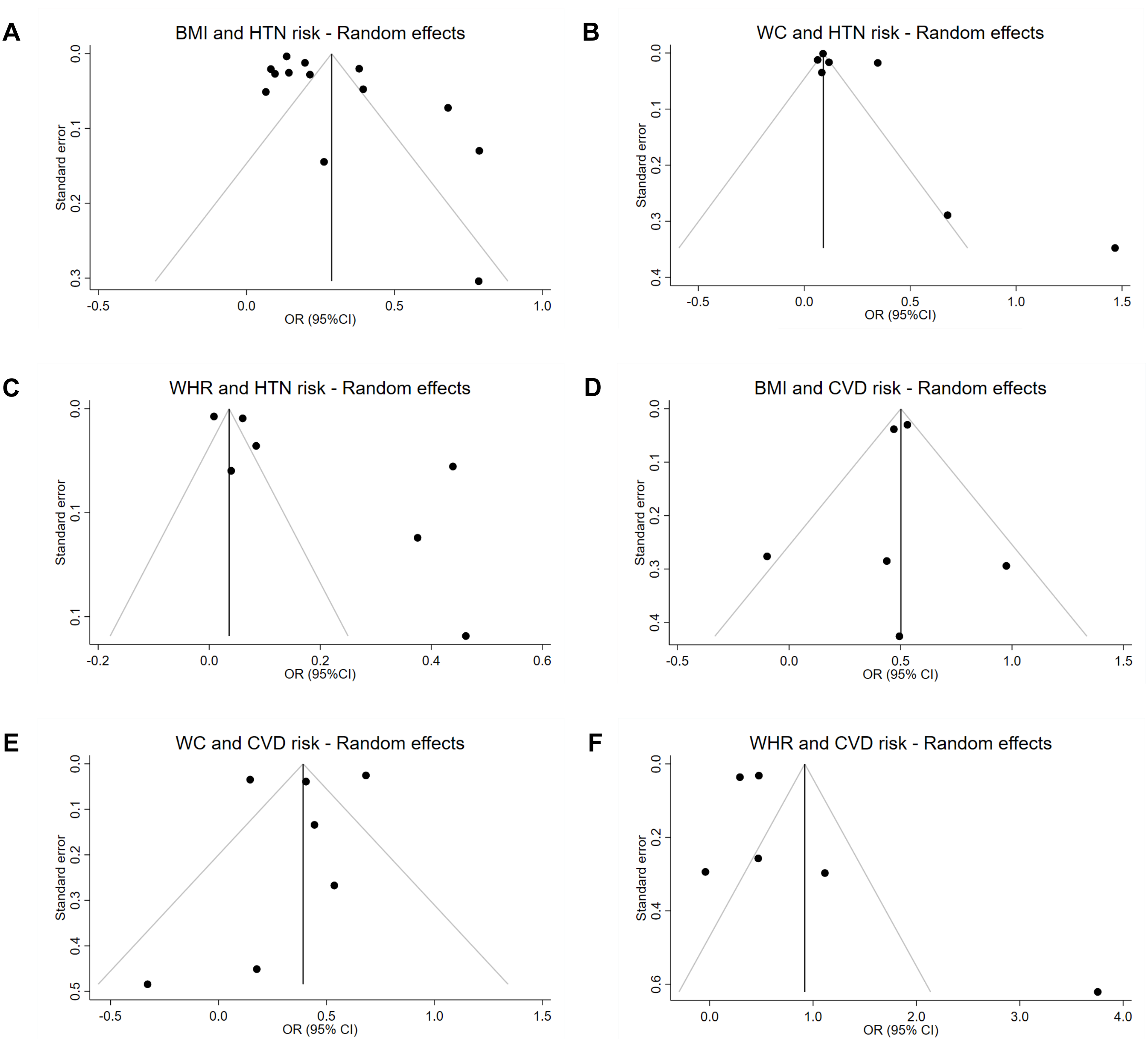
A
BMI and HTN risk - Random effects
Standard error
0.0
0.1
0.2
0.3
-0.5
0.0
0.5
1.0
OR (95%CI)
B
WC and HTN risk - Random effects
Standard error
0.0
0.1
0.2
0.3
0.4
-0.5
0.0
0.5
1.0
1.5
OR (95%CI)
C
WHR and HTN risk - Random effects
Standard error
0.0
0.1
0.1
-0.2
0.0
0.2
0.4
0.6
OR (95%CI)
D
BMI and CVD risk - Random effects
Standard error
0.0
0.1
0.2
0.3
0.4
-0.5
0.0
0.5
1.0
1.5
OR (95% CI)
E
WC and CVD risk - Random effects
Standard error
0.0
0.1
0.2
0.3
0.4
0.5
-0.5
0.0
0.5
1.0
1.5
OR (95% CI)
F
WHR and CVD risk - Random effects
Standard error
0.0
0.2
0.4
0.6
0.0
1.0
2.0
3.0
4.0
OR (95% CI)

DATA SUPPLEMENT

Associations of General and Central Adiposity with Hypertension and Cardiovascular Disease Among South Asian Populations: A Systematic Review and Meta-Analysis

Federica RE[1] MSc, Ayodipupo S. OGUNTADE[1] MSc, Bastian BOHRMANN[1] MSc, Fiona BRAGG[1,2] DPhil, Jennifer L. CARTER[1] PhD

1. Clinical Trial Service Unit and Epidemiological Studies Unit (CTSU), Nuffield Department of Population Medicine, University of Oxford, Oxford, UK.

2. MRC Population Health Research Unit, Nuffield Department of Population Health, University of Oxford, Oxford, UK

Address for correspondence
Dr Jennifer Carter
Nuffield Department of Population Health
Big Data Institute
Roosevelt Drive, Oxford
OX3 7LF, UK
jennifer.carter@ndph.ox.ac.uk

For peer review only

For peer review only - http://bmjopen.bmj.com/site/about/guidelines.xhtml

CONTENTS

Data Supplement S1. Search strategy in MEDLINE and Embase……………..……………....3

Data Supplement S2. Summary of studies included in literature review looking at the association of measures of adiposity with blood pressure (BP) and hypertension (HTN)………………………………………………………………………………………..6

Data Supplement S3. Summary of studies included in literature review looking at the association of measures of adiposity with cardiovascular disease (CVD) risk and mortality………………………………………………………………………………………12

Data Supplement S4a/S4b/S4c. Quality assessment of included studies……...……15

Data Supplement S5. Random and fixed effects model formulas.………...……...…..…17

Data Supplement S6. Mean change in systolic blood pressure (SBP, panel B) and diastolic blood pressure (DBP, panels A and C) per 5kg/m^2 higher body mass index (BMI)…………………………………………………………………………...………..18

Data Supplement S7. Mean change in systolic blood pressure (SBP, panel B) and diastolic blood pressure (DBP, panels A and C) per 10cm larger waist circumference (WC)………………………………………………………………………………………..19

Data Supplement S8. Odds ratio (OR) of hypertension per 5kg/m^2 higher body mass index (BMI, A), 10cm larger waist circumference (WC, B) and 0.1-units larger waist-to-hip ratio (WHR, C)…………………………………………………………………….…….20

Data Supplement S9. Odds ratio of cardiovascular disease for overweight vs. normal body mass index (BMI, panel A), large vs. normal waist circumference (WC, panel B), and large vs. normal waist-to-hip ratio (WHR, panel C)………………………………...21

Data Supplement S10. Additional details on sensitivity analyses results………...……22

For peer review only

For peer review only - http://bmjopen.bmj.com/site/about/guidelines.xhtml

Data Supplement S1. Search strategy for MEDLINE and Embase.

Medline (Ovid MEDLINE® Epub Ahead of Print, In-Process & Other Non-Indexed Citations, Ovid MEDLINE® Daily and Ovid MEDLINE®) search strategy

1 Anthropometry/ or body composition/ or body fat distribution/ or body weight/ or overweight/ or thinness/ or Adipose Tissue/ or Adiposity/ or body fat distribution/ or body mass index/ or body size/ or waist-hip ratio/ or Obesity/ or abdominal fat/ or intra-abdominal fat/ or subcutaneous fat, abdominal/ or subcutaneous fat/ or Waist Circumference/ or Waist-Height Ratio

2 ((abdom* or intraabdom* or central or truncal or trunk or visceral or android or gynoid) adj fat?)

3 (central adiposity or obes* or fat distribution or waist circumference or hip circumference or waist-hip ratio or waist-to-hip ratio or waist-height ratio or waist-to-height ratio or waist-to-stature ratio or WHR or WHtR or electric impedance or bio-impedance or bio-electric impedance or sagittal abdominal diameter)

4 (anthropometr* or adipos* or body weight or body mass index or quetelet index or BMI or fat mass* or body fat* or fatness or lean mass or fat-free mass or overweight or body composition or body size or skeletal mass or muscle mass)

5 exp Coronary Disease/ or exp Myocardial Ischemia/ or Myocardial Infarction/ or exp Peripheral Vascular Diseases/ or exp Cerebrovascular Disorders/ or Cardiovascular Diseases/ or exp Hypertension/ or Blood Pressure/

6 (coronary heart disease or coronary artery disease or angina* or myocardial infarction or coronary isch?emia or myocardial isch?emia or heart attack* or isch?emic heart disease or CHD or stroke* or cerebrovascular accident or cerebrovascular disorder or cerebrovascular disease or peripheral arter* disease or peripheral vascular disease or cardiovascular disease or CVD or cardiovascular risk or vascular risk or vascular disease or hypertension or HTN or blood pressure)

7 exp case-control studies/ or exp cohort studies/ or exp cross-sectional studies/ or clinical trial/ or observational study/ or Randomized Controlled Trials as Topic/

8 (cohort* or longitudinal* or prospective* or follow-up* or observational * or inciden* or case-control or retrospective or cross-sectional or population-based or randomi?ed controlled trial* or randomi?ed trial* or clinical trial* or association* or risk*)

9 (South Asia* or Afghan* or Bangladesh*or Bhutan* or India* or Maldiv* or Nepal* or Pakistan* or Sri Lanka*)

10 bangladesh/ or bhutan/ or india/ or afghanistan/ or nepal/ or pakistan/ or sri lanka/

For peer review only - http://bmjopen.bmj.com/site/about/guidelines.xhtml

11 1 or 2 or 3 or 4
12 5 or 6
13 7 or 8
14 9 or 10
15 11 and 12 and 13 and 14
16 limit 15 to (english language and humans and yr="1990-Current" and "all adult (18 plus years)")

Embase search strategy

1 Anthropometry/ or body composition/ or body fat distribution/ or body weight/ or overweight/ or thinness/ or Adipose Tissue/ or Adiposity/ or body fat distribution/ or body mass index/ or body size/ or waist-hip ratio/ or Obesity/ or abdominal fat/ or intra-abdominal fat/ or subcutaneous fat, abdominal/ or subcutaneous fat/ or Waist Circumference/ or Waist-Height Ratio/

2 ((abdom* or intraabdom* or central or truncal or trunk or visceral or android or gynoid) adj fat?)

3 (central adiposity or obes* or fat distribution or waist circumference or hip circumference or waist-hip ratio or waist-to-hip ratio or waist-height ratio or waist-to-height ratio or waist-to-stature ratio or WHR or WHtR or electric impedance or bio-impedance or bio-electric impedance or sagittal abdominal diameter)

4 (anthropometr* or adipos* or body weight or body mass index or quetelet index or BMI or fat mass* or body fat* or fatness or lean mass or fat-free mass or overweight or body composition or body size or skeletal mass or muscle mass)

5 exp Coronary Disease/ or exp Myocardial Ischemia/ or Myocardial Infarction/ or exp Peripheral Vascular Diseases/ or exp Cerebrovascular Disorders/ or Cardiovascular Diseases/ or exp Hypertension/ or Blood Pressure/

6 (coronary heart disease or coronary artery disease or angina* or myocardial infarction or coronary isch?emia or myocardial isch?emia or heart attack* or isch?emic heart disease or CHD or stroke* or cerebrovascular accident or cerebrovascular disorder or cerebrovascular disease or peripheral arter* disease or peripheral vascular disease or cardiovascular disease or CVD or cardiovascular risk or vascular risk or vascular disease or hypertension or HTN or blood pressure)

7 exp case-control studies/ or exp cohort studies/ or exp cross-sectional studies/ or clinical trial/ or observational study/ or Randomized Controlled Trials as Topic/

8 (cohort* or longitudinal* or prospective* or follow-up* or observational * or inciden* or case-control or retrospective or cross-sectional or population-based or randomi?ed controlled trial* or randomi?ed trial* or clinical trial* or association* or risk*)

For peer review only - http://bmjopen.bmj.com/site/about/guidelines.xhtml
For peer review only

9 (South Asia* or Afghan* or Bangladesh*or Bhutan* or India* or Maldiv* or Nepal* or Pakistan* or Sri Lanka*)

10 bangladesh/ or bhutan/ or india/ or afghanistan/ or nepal/ or pakistan/ or sri lanka/

11 1 or 2 or 3 or 4
12 5 or 6
13 7 or 8
14 9 or 10
15 11 and 12 and 13 and 14
16 limit 15 to (human and english language and yr="1990-Current" and (adult >18 to 64 years or aged >65+ years))

For peer review only - http://bmjopen.bmj.com/site/about/guidelines.xhtml

Data Supplement S2. Summary of studies included in literature review looking at the association of measures of adiposity with blood pressure (BP) and hypertension (HTN)

Results report associations in terms of mean change (mmHg) in BP or odds ratios (OR) of HTN. Additionally, 95% confidence intervals (95%CI) are reported where available. Unless otherwise specified, cut-offs used throughout for different measures of adiposity are: underweight BMI=<18.5kg/m^2, normal BMI=18.6-25.9kg/m^2, overweight BMI=26-29.9kg/m^2, obese BMI=≥30kg/m^2, low-risk/normal WC females=<80cm, high-risk/large WC females=≥80cm, low-risk/normal WC males=<94cm, high-risk/large WC males=≥94cm, low-risk/normal WHR females=<0.85cm, high-risk/large WHR females=≥0.85cm, low-risk/normal WHR males=<0.90cm, high-risk/large WHR males=≥90cm.
Abbreviations: BMI= body mass index (kg/m^2); WHR=waist-to-hip ratio (cm); WC=waist circumference (cm); SBP=systolic blood pressure (mmHg); DBP=diastolic blood pressure (mmHg); WHtR=waist-to-height ratio (cm); B=beta coefficient; t=two-tailed t-test.

Author (study design)	Geographical location	No. of participants (% of women)	Mean (SD) age, years	Adiposity measurements	Mean follow-up, years	Outcomes	Variables adjusted for	Results
Agrawal et al., 2014[1] (prospective cohort)	India	325 (100)	38 (N/A)	BMI, WHR	4	HTN	Age-group, education, religion, caste, employment status, standard of living	The likelihood of HTN was higher among obese women (OR: 3.86; p<0.0001) compared to women with a normal BMI. The likelihood of HTN among women with large WHR was 2.5 times higher (OR=2.47; p=0.002).
Bose et al., 2005[2] (cross-sectional)	India	150 (0)	40.7 (15.2)	BMI, WC	N/A	SBP, DBP, mean arterial pressure	Age	Regression of BMI and WC with SBP (B= 0.275, t=1.426), DBP (B= 0.009, t=0.067) and mean arterial pressure (B=0.098, t=0.065) showed that BMI did not have a significant impact on SBP, DBP, or mean arterial pressure. WC had a significant impact on SBP (B=0.506, t= 7.068), DBP (B=0.393, t=5.190) and mean arterial pressure (B=0.461, t=6.387).
Deshpande-Joshi et al., 2017[3] (cross-sectional)	India	140 (0)	32.4 (2)	BMI, WC, WHR	N/A	SBP, DBP, HTN	Not provided	Odds of HTN were highest for subjects in the highest tertile of BMI (OR: 18.9; 95% CI: 4.1–87.3) followed by large WC (OR: 11.4; 95% CI:1.6–13.0), and large WHR (OR: 6.6; 95% CI: 2.2–19.7).

For peer review only - http://bmjopen.bmj.com/site/about/guidelines.xhtml

Author (study design)	Geographical location	No. of participants (% of women)	Mean (SD) age, years	Adiposity measurements	Mean follow-up, years	Outcomes	Variables adjusted for	Results
Dhall et al., 2018[4] (cross-sectional)	India	568 (56.0)	32 (median age)	BMI, WC, WHR	N/A	SBP, DBP, HTN	Not provided	For males, large WC was significantly associated with risk of HTN (OR: 6.76; 95% CI: 2.57-17.76). Odds of HTN were found to be highest among those with risk category of WHR and conicity index in females. However, no significant association was observed.
Ganguli et al., 2013[5] (cross-sectional)	India	415 (100)	54.5 (9.4)	BMI, WC, WHR	N/A	HTN	Age, total cholesterol, exercise frequency, family history of HTN	WC was significantly associated with HTN (OR: 2.55; 95%CI: 1.07-6.06). WHR was significantly associated with HTN in the unadjusted (OR: 2.26, 95%CI: 1.39-3.68) and age-adjusted models (OR: 2.17, 95%CI: 1.31-3.59). BMI was not significantly associated with HTN in the unadjusted, age-adjusted, or multivariable-adjusted models.
Ghosh et al., 2007[6] (cross-sectional)	India	180 (N/A)	45.7 (9.3)	BMI, WC, WHR, WHtR	N/A	SBP, DBP, HTN	Age	Large WC was significantly associated with HTN (OR: 1.07; 95%CI: 1.0-1.12), along with overweight BMI (OR: 1.17; 95%CI: 1.04-1.32), large WHR (OR: 1.09; 95%CI: 1.01-1.17), and large WHtR (OR: 1.12; 95%CI: 1.03-1.22).
Hazarika et al., 2004[7] (cross-sectional)	India	3180 (54.7)	Partici-pants aged >30	BMI, WHR	N/A	HTN	Age, sex, marital status, type of work, alcohol, smoking, tobacco chewing	Overweight and BMI were significantly associated with HTN (OR overweight: 1.95; 95%CI: 1.37-2.78 and OR obese: 3.10; 95%CI: 1.17-8.22). Large WHR was also associated with HTN (OR: 1.54; 95%CI: 1.25-1.90).

For peer review only - http://bmjopen.bmj.com/site/about/guidelines.xhtml

Author (study design)	Geographical location	No. of participants (% of women)	Mean (SD) age, years	Adiposity measurements	Mean follow-up, years	Outcomes	Variables adjusted for	Results
Islam et al., 2016[8] (cross-sectional)	Bangladesh	3104 (65.0)	Not reported	BMI, WC	N/A	HTN	Age, education, diabetes	Among both males and females, BMI was significantly associated with mild, moderate, and severe hypertension. The same pattern followed for quartiles of WC, though associations appeared stronger than for BMI *(see full-text, Table 3 for details)*.
Kaur et al., 2008[9] (cross-sectional)	India	2148 (0)	40.5 (11.6)	BMI, WC, WHR, WHtR		SBP, DBP, HTN	Age	All four anthropometric indices showed a significant unadjusted OR for HTN across the quintiles. BMI and WC showed significant age-adjusted OR in all quintiles. WC showed a significant increase in age and BMI adjusted OR in the last two quintiles (*see full-text for quintiles).*
Kaur et al., 2012[10] (cross-sectional)	India	10463 (53.2)	Not reported	BMI, WC		HTN	Age	Odds of HTN were higher in the general obesity category (BMI ≥27.50kg/m^2) compared to the highest central obesity category (WC ≥80cm), OR BMI: 3.25; 95%CI: 2.57-4.11 and OR WC: 2.51; 95%CI: 2.12-2.98.
Khanam et al., 2019[11] (cross-sectional)	Bangladesh	1810 (52.3)	Male median: 50 (IQR 42–60) Female median: 47 (IQR 40–57)	BMI, WC		HTN	Age, education, wealth status, smoking, fruit and vegetables (BMI models adjustments unavailable)	Compared to healthy weight, overweight BMI was significantly associated with greater risk of HTN in both males (OR: 2.9; 95%CI: 1.8-4.6) and females (OR: 1.6; 95%CI: 1.1-2.4). Odds of HTN were fourfold higher among males (OR: 4.0; 95%CI: 2.5-6.4) and threefold higher among females (OR: 2.8; 95%CI: 2.0-4.1) with large WC.
Meshram et al., 2012[12] (cross-sectional)	India	4193 (54.9)	43.3 (14.9) for males and 42.4 (14.4) for females	BMI, WC	N/A	HTN	Age, sex, education, wealth index, alcohol	Large WC and overweight BMI were associated with two-fold (OR for abdominal obesity=1.7; 95%CI=1.24-2.33, OR for

For peer review only - http://bmjopen.bmj.com/site/about/guidelines.xhtml
For peer review only

Author (study design)	Geographical location	No. of participants (% of women)	Mean (SD) age, years	Adiposity measurements	Mean follow-up, years	Outcomes	Variables adjusted for	Results
								BMI=1.7; 95%CI=1.35-2.21) increase in risk of HTN.
Meshram et al., 2016[13] (cross-sectional)	India	8969 (49.5)	38.2 (15.4)	BMI, WC, WHR	N/A	HTN	Age, sex, education, occupation	Overweight individuals had 2.1 times higher risk of HTN (2.17; 95%CI 1.68-2.81), while individuals with large WC had two times higher risk (OR 1.96; CI 1.61-2.42). Individuals with large WHR had 1.4 times higher risk of HTN (OR 1.35; 95%CI 1.13-1.62).
Shriraam et al., 2021[14] (cross-sectional)	India	502 (57.8)	55.1 (10.86)	BMI, WC	N/A	HTN	Age, sex, occupation, physical activity, diabetes	Being overweight was not significantly associated with increased risk of HTN (OR: 1.27; 95%CI: 0.76-2.13). However, being obese was (OR: 9.75; 95%CI: 2.06-46.19). WC was significantly associated with increased risk of HTN (OR:1.62; 95%CI: 1.07-2.48).
Simmons et al., 2021[15] (cross-sectional)	Bangladesh	8019 (53)	See categories in full-text	BMI, WC, WHR, WHtR	N/A	HTN	Age, residence, education, fruits and vegetables, alcohol, physical activity	Risk of HTN was higher among females and males who were overweight (OR: 1.35; 95%CI: 1.23-1.62 and OR: 2.40; 95%CI: 2.34-2.62) and obese (OR: 1.68; 95%CI: 1.44-1.89 and OR: 2.62; 95%CI: 2.02-2.94). A moderate WC, defined as 94-102cm in males and 80-88cm in females, was associated with higher risk of HTN among both groups (OR females: 1.72; 95%CI: 1.64-1.91; OR males: 2.14; 95%CI: 2.05-2.28). A stronger pattern was observed for large WC (OR: 2.52; 95%CI: 2.05-2.98 and OR: 2.65; 95%CI: 2.34-2.85). Patterns were similar for WHR and WHtR.

For peer review only
For peer review only - http://bmjopen.bmj.com/site/about/guidelines.xhtml

Author (study design)	Geographical location	No. of participants (% of women)	Mean (SD) age, years	Adiposity measurements	Mean follow-up, years	Outcomes	Variables adjusted for	Results
Singh et al., 2012[16] (cross-sectional)	India	3118 (36.5)	Not reported	BMI, WHR	N/A	HTN	Tobacco, alcohol	BMI was significantly associated with increased risk of HTN (OR: 1.52; 95%CI: 1.25-1.85), as was WHR (OR: 1.65; 95%CI: 1.36-2.00).
Taing et al., 2016[17] (cross-sectional)	India	7601 (50.1)	40 (11)	BMI, WC, HC, WHR, WHtR	N/A	SBP, DBP, mean arterial pressure	Education, location, WC, BMI	Every 5 kg/m^2 greater BMI or 10cm wider WC was associated with a 5 and 4mmHg higher SBP, and a 4 and 3mmHg higher DBP. The association between WC and DBP was stronger than the that of BMI and DBP.
Tselha et al., 2019[18] (cross-sectional)	India	214 (53.2)	34.0 (16.0) in males and 33.7 (11.4) in females	BMI, WC, WHR, WHtR	N/A	HTN	Not provided	In males, WHR followed by WC and BMI were found to be associated with HTN: OR: 1.48 (96%CI:-0.59-3.72); 1.16 (95%CI:-0.37-3.65); and 1.11 (95%CI:0.11-11.20). In females, WHtR, BMI and WHR were found to be the strongest predictors of HTN: OR: 6.82 (95%CI: -2.68-17.39); 1.31 (95%CI: - 0.33-5.30); and 1.06 (95%CI: 0.21-3.17), respectively.
Venkatramana et al., 2002[19] (cross-sectional)	India	212 (0)	Urban: 47.4 (9.1); rural: 40.8 (14.2)	BMI, WC	N/A	SBP, DBP	Age, smoking, physical activity	BMI did not have significant effects on SBP (B=0.050, p=0.891) and DBP (B=0.225, p=0.544) in the urban population. However, it did have significant effects in the rural population (SBP B=1.182, p<0.001; DBP B=1.047, p=0.005). WC did not have significant effects on SBP (B=0.224, p=0.660) or DBP (B=0.140, p=0.788) in the urban population or the rural population (SBP: B=-0.990, p=0.059; DBP: B=0.140, p=0.788).

For peer review only - http://bmjopen.bmj.com/site/about/guidelines.xhtml
For peer review only

Author (study design)	Geographical location	No. of participants (% of women)	Mean (SD) age, years	Adiposity measurements	Mean follow-up, years	Outcomes	Variables adjusted for	Results
Vikram et al., 2016[20] (cross-sectional)	India	509 (45.3)	40.0 (8.6) in males and 38.7 (9.2) in females	BMI, WC, WHR, WHtR	N/A	HTN	Crude	In males, being overweight (OR: 2.12; 95%CI 1.07-4.37), having a large WC (OR: 2.65; 95%CI: 1.33-5.26), WHR (OR: 2.27; 95%CI: 0.91-5.61), and WHtR (OR: 3.85; 95%CI: 1.57-9.43) was associated with HTN. In females, overweight BMI (OR: 4.00; 95%CI: 1.16-13.80) and large WC (OR: 5.25; 95%CI: 1.52-18.11) were significantly associated with HTN. Large WHR (OR: 4.21; 95%CI: 0.96-18.47) was not statistically significant.
Yadav et al., 2008[21] (cross-sectional)	India	1746 (31.8)	49.8 (11.5)	BMI, WC, WHR	N/A	HTN	Age, sex	Being overweight was associated with HTN (OR: 2.2; 95%CI: 1.5-3.1). Associations were stronger for large WC (OR: 4.1; 95%CI: 2.6-6.4), but weaker for large WHR (OR: 1.56; 95%CI: 1.37-1.79).

For peer review only - http://bmjopen.bmj.com/site/about/guidelines.xhtml
For peer review only

Data Supplement S3. Summary of studies included in literature review looking at the association of measures of adiposity with cardiovascular disease (CVD) risk and mortality

Results report associations in terms hazard ratios (HR), or relative risk (RR). Additionally, 95% confidence intervals (95%CI) and p-values (p) are reported where available. Unless otherwise specified, cut-offs used throughout for different measures of adiposity are: underweight BMI=<18.5kg/m^2, normal BMI=18.6-25.9kg/m^2, overweight BMI=26-29.9kg/m^2, obese BMI=≥30kg/m^2, low-risk/normal WC females=<80cm, high-risk/large WC females=≥80cm, low-risk/normal WC males=<94cm, high-risk/large WC males=≥94cm, low-risk/normal WHR females=<0.85cm, high-risk/large WHR females=≥0.85cm, low-risk/normal WHR males=<0.90cm, high-risk/large WHR males=≥90cm.

Abbreviations: BMI=body mass index (kg/m^2); WC=waist circumference (cm); WHR=waist-to-hip ratio (cm); CHD=coronary heart/artery disease; HC=hip circumference.

Author (study design)	Geographical location	No. of participants (% of women)	Mean (SD) age, years	Adiposity measurements	Mean follow-up, years	Outcomes	Adjustments	Results
Banerjee et al., 2021[22] (cross-sectional)	India	31464 (N/A)	N/A, participants >60	BMI, WC, WHR	N/A	CVD	Age, sex, education, marital status, working status, tobacco, alcohol, physical activity, monthly per-capital consumption expenditure, religion, caste, place of residence, region	Likelihood of CVD was higher among adults who were overweight (OR: 1.60; CI: 1.48–1.72), had a large WC (OR: 1.50; CI: 1.39–1.62) and high-risk WHR (AOR: 1.34; CI: 1.25–1.44), compared to adults with normal BMI and those who do not have a high-risk WC and high risk WHR.
Bodkhe et al., 2019[23] (cross-sectional)	India	1190 (51)	N/A, participants >60	BMI, WC, WHR	N/A	CHD	Age, sex, education, socioeconomic status, physical activity, HTN, diabetes, tobacco	No significant associations between risk of CHD and obesity (OR for BMI: 1.64; 95%CI: 0.71-3.77; OR for WC: 0.72; 95%CI: 0.28-1.87; OR for WHR: 0.96; 95%CI: 0.54-1.71).
Bramhankar et al., 2021[24] (cross-sectional)	India	37536 (N/A)	N/A, participants >45	BMI, WC, WHR	N/A	CVD	Age, sex, education, employment, residence, caste, wealth quintile, region, smoking, alcohol	Adults were 2.3 times more likely (OR: 2.33; 95%CI: 2.2-2.5) by obesity, 61% more likely (OR: 1.61; 95%CI: 1.63-1.63) by high-risk WHR and 98% more likely (OR: 1.98; 95%CI: 1.9-2.1) by high-risk WC to develop CVD than their normal-weight individuals.

For peer review only - http://bmjopen.bmj.com/site/about/guidelines.xhtml

Author (study design)	Geographical location	No. of participants (% of women)	Mean (SD) age, years	Adiposity measurements	Mean follow-up, years	Outcomes	Adjustments	Results
Cameron et al., 2012[25] (prospective cohort)	Mauritius (South Asian Mauritius and Africa Mauritius reported separately)	7,979	Not reported	BMI, WC, HC	15.1	Death from CVD	Age, sex, smoking	In the South Asian population, high-risk WC was significantly related to CVD death in males (OR: 1.8; 95%CI: 1.2-2.7) and females (OR: 1.5; 95%CI: 1.1-2.2), compared with those below the obesity cut-off, when adjusting for hip circumference.
Latheef et al., 2018[26] (case-control)	India	165 (0)	Not reported	WC, WHR	N/A	CAD	Not provided	Association of anthropometric variables with CAD:<br>WC- OR: 1.16; 95%CI: 1.08-1.24. WHR- OR: 42.69; 95%CI: 10.28-177.20.
Nishtar et al., 2008[27] (cross-sectional)	Pakistan	400 (16)	Cases: 51.2 (9.5)<br>Controls: 48.2 (9.5)	BMI, WC, WHR	N/A	CAD	Age, urban/rural, family history of CAD, smoking, diabetes, HTN, BP	A significant association of WC (OR: 1.02; 95%CI: 1.00-1.04) and WHR (OR: 1.06; 95%CI: 1.03-1.10) was found with CAD. The association was not significant for BMI (OR: 1.02; 95%CI: 0.96-1.08).
Patil et al., 2004[28] (case-control)	India	Cases 111 (20.7)<br>Controls 222 (20.7)	*See categories in full-text*	BMI, WHR	N/A	Acute myocardial infarction	Crude	Large WHR was associated with increased risk of acute MI (OR: 2.50; 95%CI: 1.52-4.10). Overweight BMI, was also associated with increased risk (OR: 1.55; 95%CI: 0.88-2.69) but was not significant.

For peer review only

For peer review only - http://bmjopen.bmj.com/site/about/guidelines.xhtml

Author (study design)	Geographical location	No. of participants (% of women)	Mean (SD) age, years	Adiposity measurements	Mean follow-up, years	Outcomes	Adjustments	Results
Prabhakar et al., 2020[29] (case-control)	India	Cases: 144 (30.5) Controls: 144 (50.0)	*See categories in full-text*	BMI, WC	N/A	Stroke	Age, sex, smoking, alcohol, BP, diabetes, total cholesterol, HDL	Large WC in both men and women were not significantly associated with increased risk of stroke (OR: 1.33; 95%CI: 0.69-2.59 and OR: 1.06; 95%CI: 0.33-3.37 respectively). Being overweight was also not significantly associated with stroke (OR: 1.06; 95%CI: 0.33-3.37).
Shah et al., 2017[30] (case-control)	India	300 (22.0)	56.1 (10.7)	BMI, WC, WHR	N/A	CAD	Not provided	Overweight BMI significantly associated with risk of CAD (OR: 2.65; 95%CI: 1.49-4.72). Large WHR not significantly associated with risk of CAD (OR: 1.60; 95%CI: 0.97-2.66). High WC significantly associated with risk of CAD (OR: 1.71; 95%CI: 1.01-2.88).

For peer review only - http://bmjopen.bmj.com/site/about/guidelines.xhtml

Data Supplement S4a, S4b, and S4c. Quality assessment of included studies

Performed using the Newcastle-Ottawa Scale adapted to cohort, case-control, and cross-sectional studies. Criteria that were met were marked with a 1, whereas criteria that were not met were marked with a 0.

S4a: COHORT STUDIES

First author, publication year	SELECTION				COMPARABILITY		OUTCOME			Total (9/9)
	Representative of exposed cohort	Selection of non-exposed	Exposure ascertainment	Outcome absent at start	Main factor controlled for	Additional factors controlled for	Assessment of outcomes	Sufficient follow-up time (>2 years)	Adequacy of follow-up	
Agrawal et al., 2014	1	1	1	0	1	1	1	0	1	6/9
Cameron et al., 2012	1	1	1	1	1	0	0	1	1	7/9

S4b: CASE-CONTROL STUDIES

First author, publication year	SELECTION				COMPARABILITY		OUTCOME			Total (9/9)
	Adequate case definition	Representativeness of cases	Selection of controls	Definition of controls	Main factor controlled for	Additional factors controlled	Ascertainment of exposure	Same ascertainment cases and controls	Non-response rate	
Latheef et al., 2018	1	0	0	1	1	0	1	1	0	5/9
Patil et al., 2004	1	1	0	1	1	0	1	1	0	6/9
Prabhakar et al., 2020	1	0	1	1	1	0	1	1	0	6/9
Nishtar et al., 2008	1	0	0	1	1	0	1	1	0	5/9
Shah et al., 2017	1	0	0	1	1	0	0	1	0	4/9

For peer review only - http://bmjopen.bmj.com/site/about/guidelines.xhtml

S4c: CROSS-SECTIONAL STUDIES

First author, publication year	SELECTION				COMPARABILITY		OUTCOME		Total (10/10)
	Representativeness of sample	Sample size	Ascertainment of exposure (max 2)	Non-response rate	Main factor controlled for	Additional factors controlled	Assessment of outcome (max 2)	Same ascertainment cases and controls	
Banerjee et al., 2021	1	1	0	0	1	1	1	1	6/10
Bodkhe et al., 2019	1	0	2	0	1	1	1	1	7/10
Bose et al., 2005	1	0	2	0	1	0	2	1	7/10
Bramhankar et al., 2021	1	0	0	0	1	1	1	1	5/10
Deshpande-Joshi et al., 2017	0	1	2	0	0	0	2	1	6/10
Dhall et al., 2018	0	0	2	0	0	0	2	1	5/10
Ganguli et al., 2013	1	0	2	0	1	1	2	1	8/10
Ghosh et al., 2007	0	0	2	0	0	0	2	1	5/10
Hazarika et al., 2004	1	1	2	0	1	1	2	1	9/10
Islam et al., 2016	1	1	2	0	1	1	2	1	9/10
Kaur et al., 2008	0	0	2	0	0	0	2	1	5/10
Kaur et al., 2012	0	1	2	0	1	0	2	1	7/10
Khanam et al., 2019	1	1	2	0	1	1	2	1	9/10
Meshram et al., 2012	0	1	2	0	1	1	2	1	8/10
Meshram et al., 2016	1	1	2	0	1	1	2	1	9/10
Shritaam et al., 2021	1	1	2	0	1	1	2	1	9/10
Simmons et al., 2021	0	0	1	0	1	1	1	1	5/10
Singh et al., 2012	0	0	2	0	1	0	2	1	6/10
Taing et al., 2016	1	0	2	0	0	0	2	1	6/10
Tselha et al., 2019	0	0	2	0	0	0	2	1	5/10
Venkatramana et al., 2002	1	0	2	0	1	1	2	1	8/10
Vikram et al., 2016	1	0	2	0	0	0	2	1	6/10
Yadav et al., 2008	0	1	2	1	1	1	2	1	9/10

For peer review only

For peer review only - http://bmjopen.bmj.com/site/about/guidelines.xhtml

Data Supplement S5. Random and fixed effects model formulas.[31–33]

Fixed-effects model formula

The fixed-effects model assumes that all included studies come from the same underlying population and that any variation in the study results is due to sampling error. In this model, the effect size is assumed to be the same for all studies, and any differences between study results are solely due to chance.

$$\theta_{FE} = \frac{\sum_{i=1}^{k} w_i \cdot \theta_i}{\sum_{i=1}^{k} w_i}$$

Where:

- θ_{FE} is the estimated overall effect size.
- k is the number of studies.
- w_i is the weight assigned to each study (based on sample size and precision).
- θ_i is the effect size estimate for each study.

Random-effects model formula

The random-effects model considers not only the within-study variability but also the between-study variability. It assumes that the true effect size can vary between studies due to both sampling error and real differences in effect size between the studies.

$$\theta_{RE} = \frac{\sum_{i=1}^{k} w_i \cdot \theta_i}{\sum_{i=1}^{k} w_i} \text{ and } w_i = \frac{1}{(\tau^2 + \theta_i^2)}$$

Where:

- θ_{RE} is the estimated overall effect size in the random-effects model.
- θ_i^2 is the within-study variance of each study.
- τ^2 is the estimated between-study variance, which represents the additional variability between studies.

For peer review only

For peer review only - http://bmjopen.bmj.com/site/about/guidelines.xhtml

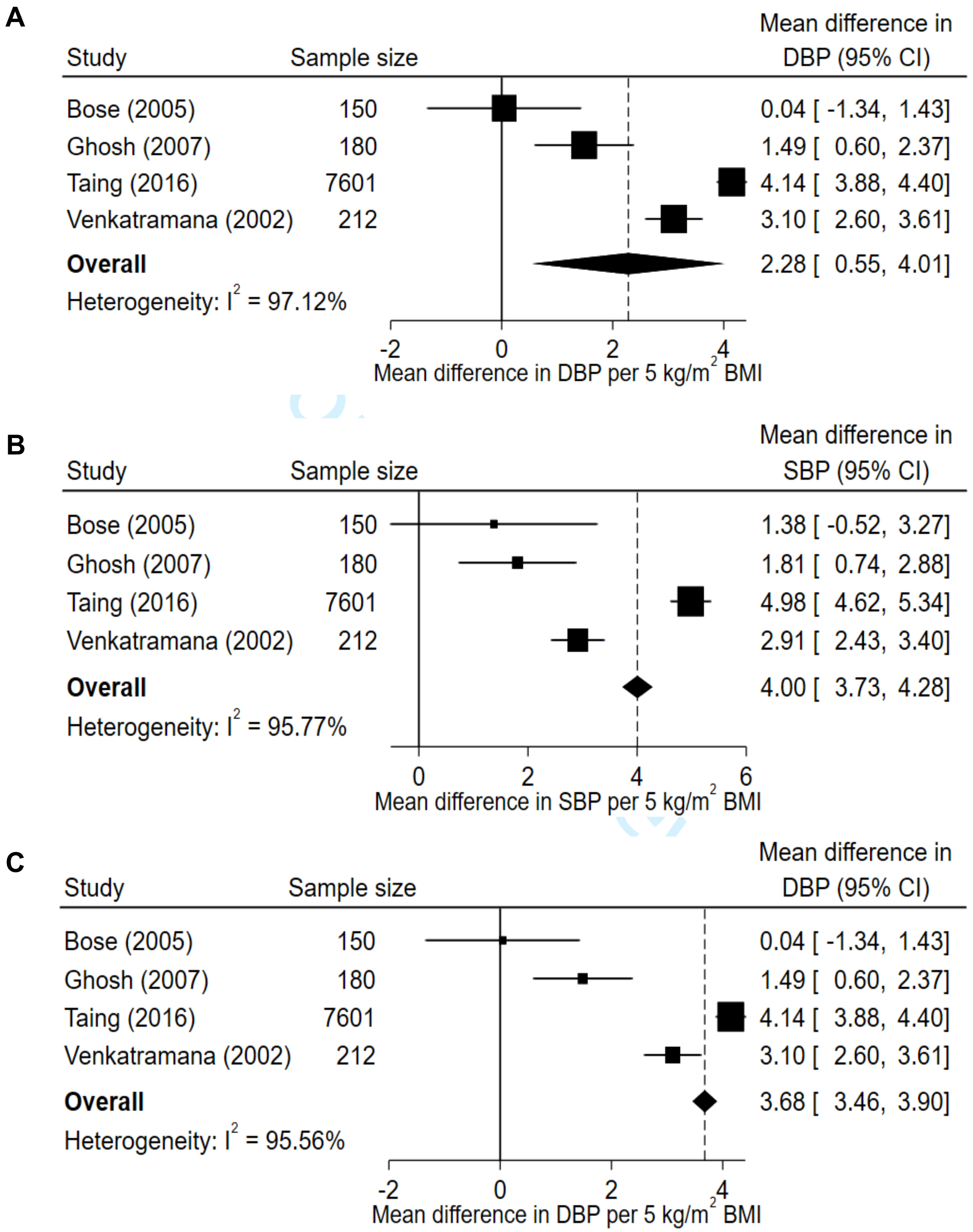

Data Supplement S6. Mean change in systolic blood pressure (SBP, panel B) and diastolic blood pressure (DBP, panels A and C) per 5kg/m^2 higher body mass index (BMI)
Random effects (A) and fixed-effects (B and C) models were applied on four studies reporting associations of blood pressure and BMI. The total number of participants was 8,143.
Abbreviations: 95% confidence interval (95%CI).

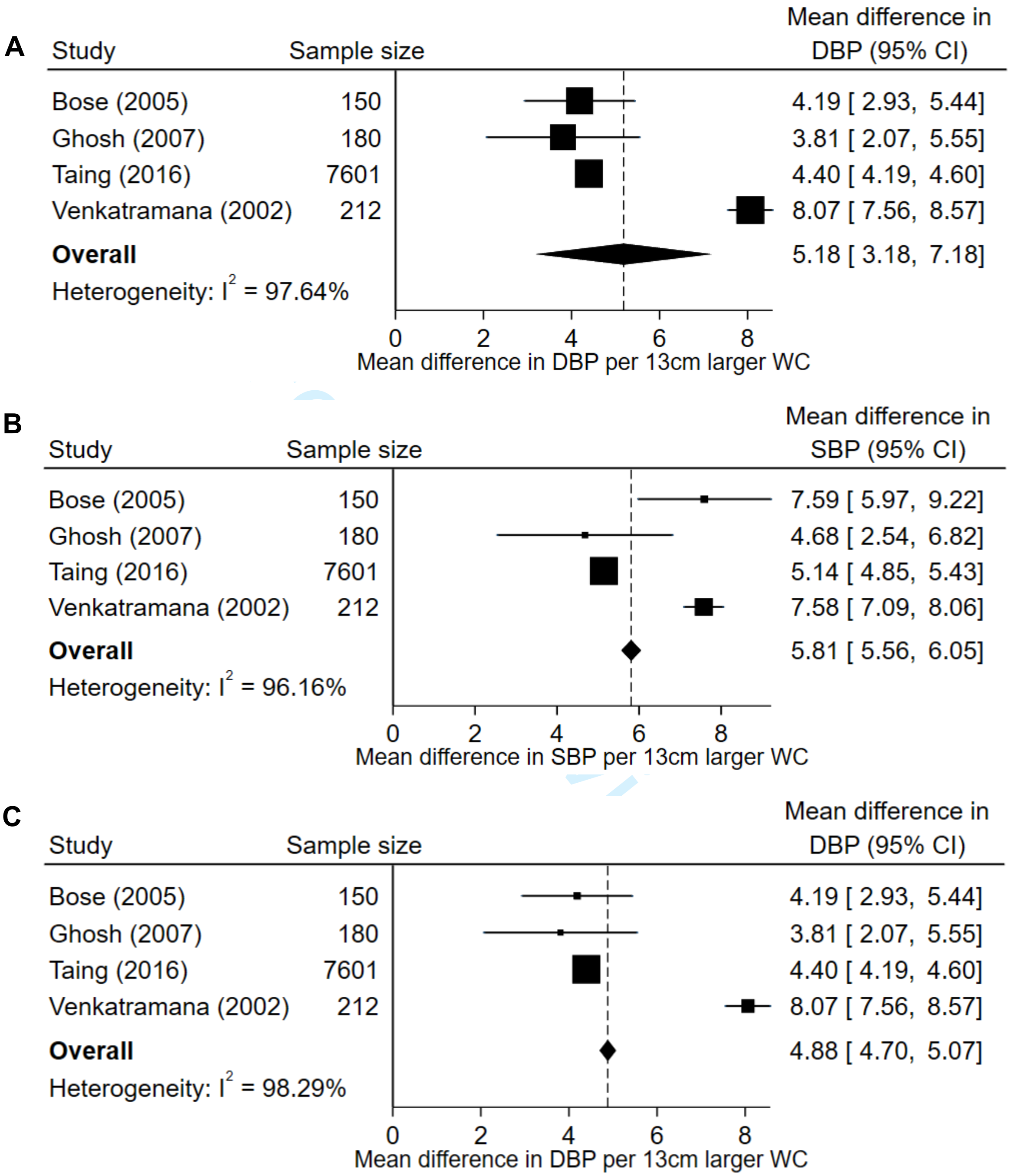

Data Supplement S7. Mean change in systolic blood pressure (SBP, panel B) and diastolic blood pressure (DBP, panels A and C) per 13cm larger waist circumference (WC)

Random effects (A) and fixed-effects (B and C) models were applied on four studies reporting associations of blood pressure and WC. The total number of participants was 8,143.

Abbreviations: 95% confidence interval (95%CI).

For peer review only - http://bmjopen.bmj.com/site/about/guidelines.xhtml

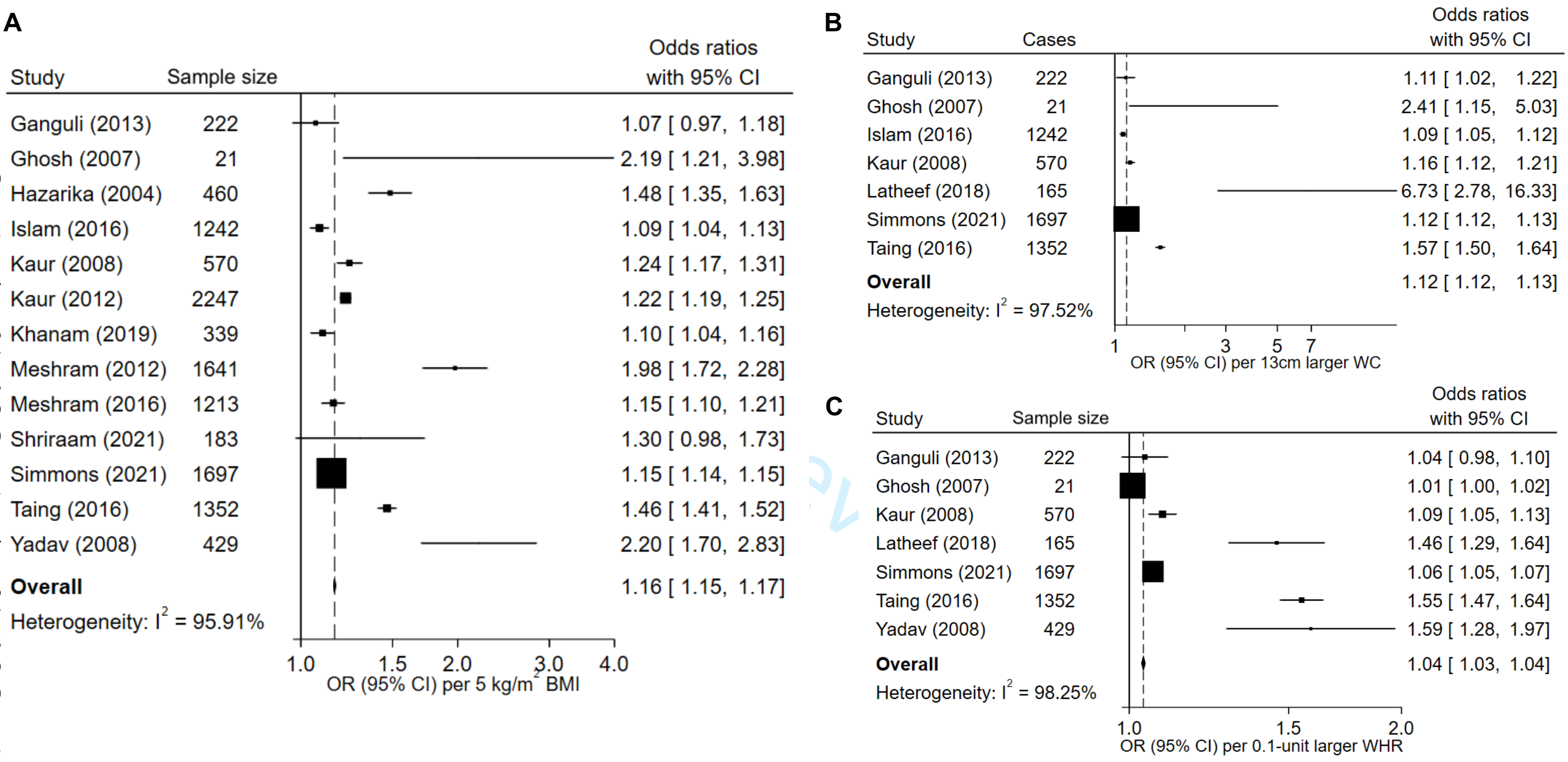

Data Supplement S8. Odds ratio (OR) of hypertension per 5kg/m^2 higher body mass index (BMI, A), 13cm larger waist circumference (WC, B) and 0.1-units larger waist-to-hip ratio (WHR, C)
A fixed-effects model was used on 13 studies looking at BMI, seven studies looking at WC and seven studies looking at WHR. The total number of participants was 11,636 for panel A, 4,899 for B, and 4,456 or panel C. *Abbreviations: 95% confidence interval (95%CI).*

For peer review only - http://bmjopen.bmj.com/site/about/guidelines.xhtml

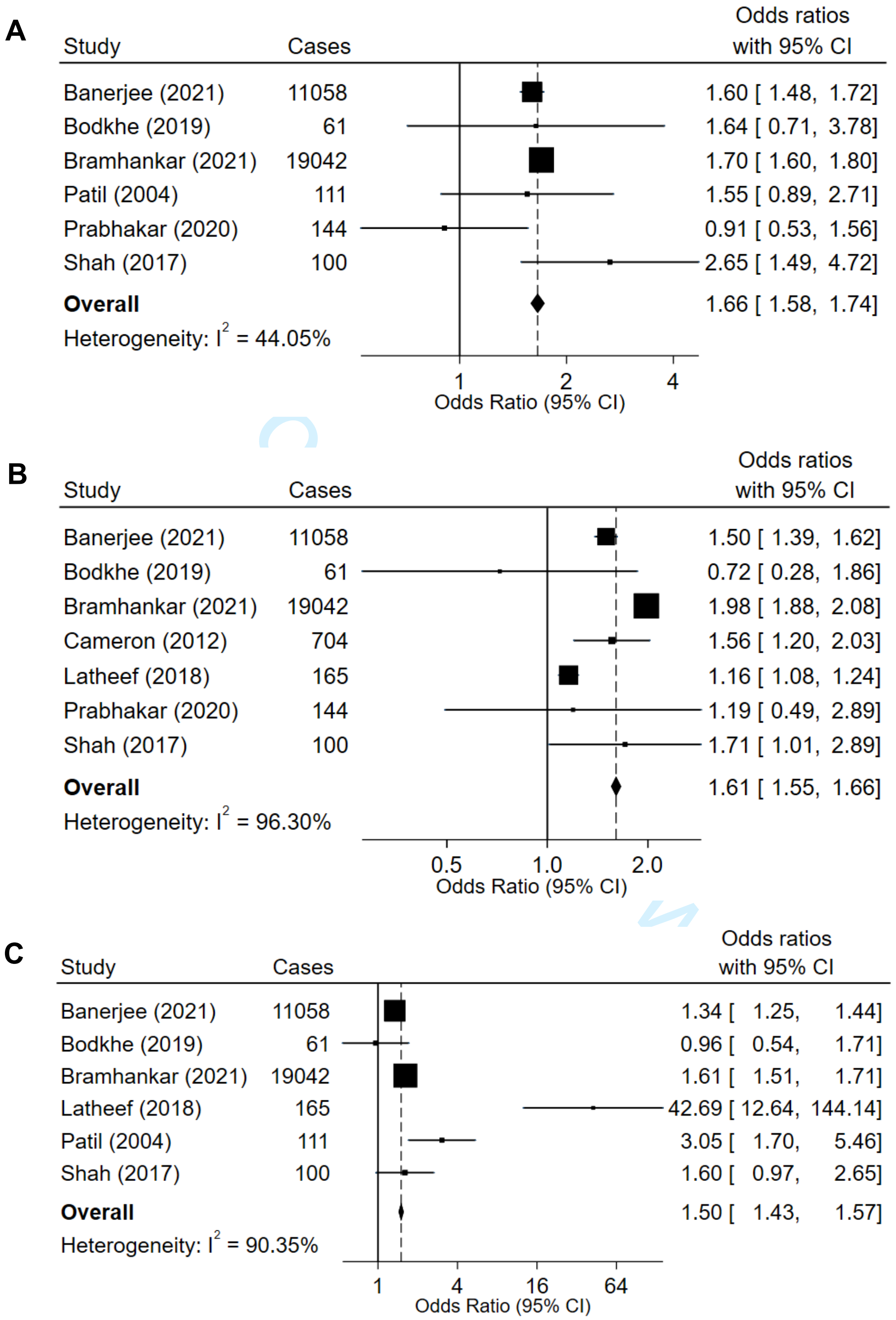

Data Supplement S9. Odds ratio of cardiovascular disease for overweight vs. normal body mass index (BMI, panel A), large vs. normal waist circumference (WC, panel B), and large vs. normal waist-to-hip ratio (WHR, panel C)

A fixed-effects model was used on six studies looking at BMI, seven studies looking at WC, and six studies looking at WHR. *Abbreviations: 95% confidence interval (95%CI).*

For peer review only - http://bmjopen.bmj.com/site/about/guidelines.xhtml

Data Supplement S10. Additional details on sensitivity analyses results.

Results were not substantively different after sensitivity analyses. For the studies looking at the association of a 5kg/m^2 increase in BMI with HTN, removing the study by Meshram *et al*. decreased the odds ratio from 1.33 (95%CI: 1.18-1.51) to 1.28 (95%CI: 1.14-1.42).[12] Similar results were seen when removing the study by Yadav et al. (OR: 1.28; 95%CI: 1.15-1.43).[21] Individual removal of several other studies resulted in an increase in magnitude of associations to 1.36 (1.20-1.55).[5,8,11,13,15] For studies looking at the odds of HTN per 10cm larger WC, removing the study by Simmons et al. resulted in an increase in estimates from 1.33 (1.04-1.69) to 1.57 (1.28-1.93).[15] For studies looking at the odds of HTN per 0.1-unit larger WHR, removing the studies by Ghosh et al. [6] increased the strength of associations from 1.18 (1.02-1.37) to 1.26 (1.07-1.48).

For peer review only

For peer review only - http://bmjopen.bmj.com/site/about/guidelines.xhtml

DATA SUPPLEMENT REFERENCE LIST

1. Agrawal P, Gupta K, Aboyans V, Agrawal S. Women's health in India: the role of body mass index. Health Care Women Int. 2014;36(3):320–41.

2. Bose K, Ghosh A, Roy S, Gangopadhyay S. The relationship of age, body mass index and waist circumference with blood pressure in Bengalee Hindu male jute mill workers of Belur, West Bengal, India. Anthropology Anz. 2005;63(2):205–12.

3. Deshpande-Joshi S, Rao S. Differential risk of hypertension among lean and non-lean rural subjects in relation to decadal changes in anthropometry. J Am Coll Nutr. 2018;37(5):380–6.

4. Dhall M, Devi K, Nilupher A, Gupta U, Tyagi R, Kapoor S. Hypertension and its correlate with general and central adiposity: A study among urban population of Delhi. Diabetes Metab Syndr. 2018;12(6):881–4.

5. Ganguli D, Das N, Saha I, Chaudhuri D, Ghosh S, Dey S. Risk factors for hypertension in a population-based sample of postmenopausal women in Kolkata, West Bengal, India. Asia Pac J Public Health. 2013;25(5):388–97.

6. Ghosh J, Bandyopadhyay A. Comparative evaluation of obesity measures: relationship with blood pressure and hypertension. Singapore Med J. 2007;48(3):232–5.

7. Hazarika N, Narain K, Biswas D, Kalita H, Mahanta J. Hypertension in the native rural population of Assam. Natl Med J India. 2004;17:300–4.

8. Islam FMA, Bhuiyan A, Chakrabarti R, Rahman MA, Kanagasingam Y, Hiller JE. Undiagnosed hypertension in a rural district in Bangladesh: The Bangladesh Population-based Diabetes and Eye Study (BPDES). J Hum Hypertens. 2016;30(4):252–9.

9. Kaur P, Radhakrishnan E, Sankarasubbaiyan S, Rao S, Kondalsamy-Chennakesavan S, Rao T, et al. A Comparison of Anthropometric Indices for Predicting Hypertension and Type 2 Siabetes in a Male Industrial Population of Chennai, South India. Ethnicity and Disease. 2008;18:31–26.

10. Kaur P, Rao S, Radhakrishnan E, Rajasekar D, Gupte M. Prevalence, awareness, treatment, control and risk factors for hypertension in a rural population in South India. Int J Public Health. 2012;57(1):87–94.

11. Khanam R, Ahmed S, Rahman S, Kibria G, Syed J, Khan A, et al. Prevalence and factors associated with hypertension among adults in rural Sylhet district of Bangladesh: a cross-sectional study. BMJ Open [Internet]. 2019 [cited 2022 Jul 24];9(10). Available from: https://bmjopen.bmj.com/lookup/doi/10.1136/bmjopen-2018-026722

For peer review only - http://bmjopen.bmj.com/site/about/guidelines.xhtml

12. Meshram II, Arlappa N, Balkrishna N, Rao KM, Laxmaiah A, Brahmam GNV. Prevalence of hypertension, its correlates and awareness among adult tribal population of Kerala state, India. J Postgrad Med. 2012;58(4):255–61.

13. Meshram II, Vishnu Vardhana Rao M, Sudershan Rao V, Laxmaiah A, Polasa K. Regional variation in the prevalence of overweight/obesity, hypertension and diabetes and their correlates among the adult rural population in India. Br J Nutr. 2016;115(7):1265–72.

14. Shriraam V, Mahadevan S, Arumugam P. Prevalence and risk factors of diabetes, hypertension and other non-communicable diseases in a tribal population in South India. Indian J Endocr Metab. 2021;25(4):313.

15. Simmons S, Hagan Jr. J, Schack T. The influence of anthropometric indices and intermediary determinants of hypertension in Bangladesh. IJERPH [Internet]. 2021 [cited 2022 Jul 24];18(11). Available from: https://www.mdpi.com/1660-4601/18/11/5646

16. Singh R, Mukherjee M, Kumar R, Singh R, Pal R. Study of risk factors of coronary heart disease in urban slums of Patna. Nepal J Epidemiology. 2012;2(3):205–12.

17. Taing K, Farkouh M, Moineddin R, Tu J, Jha P. Age and sex-specific associations of anthropometric measures of adiposity with blood pressure and hypertension in India: a cross-sectional study. BMC Cardiovasc Disord. 2016;16(1).

18. Tselha N, Shimrah C, Kulshreshtha M, Devi N. Association between hypertension and adiposity indicators: A study among the Muslim population of Uttar Pradesh. Diabetes Metab Syndr. 2019;13(4):2335–8.

19. Venkatramana P, Reddy P. Association of overall and abdominal obesity with coronary heart disease risk factors: comparison between urban and rural Indian men. Asia Pac J Clin Nutr. 2002;11(1):66–71.

20. Vikram N, Latifi A, Misra A, Luthra K, Bhatt S, Guleria R, et al. Waist-to-height ratio compared to standard obesity measures as predictor of cardiometabolic risk factors in Asian Indians in North India. Metab Syndr Relat Disord. 2016;14(10):492–9.

21. Yadav S, Boddula R, Genitta G, Bhatia V, Bansal B, Kongara S, et al. Prevalence & risk factors of pre-hypertension & hypertension in an affluent north Indian population. Indian J Med Res. 2008;(128):712–20.

22. Banerjee S, Kumar P, Srivastava S, Banerjee A. Association of anthropometric measures of obesity and physical Activity with ardiovascular diseases among older adults: evidence from a cross-sectional survey, 2017–18. Gaipov A, editor. PLoS ONE [Internet]. 2021 [cited 2022 Jul 24];16(12). Available from: https://dx.plos.org/10.1371/journal.pone.0260148

For peer review only - http://bmjopen.bmj.com/site/about/guidelines.xhtml

23. Bodkhe S, Jajoo S, Jajoo U, Ingle S, Gupta S, Taksande B. Epidemiology of confirmed coronary heart disease among population older than 60 years in rural central India—A community-based cross-sectional study. Indian Heart Journal. 2019;71(1):39–44.

24. Bramhankar M, Pandey M, Rana G, Rai B, Mishra N, Shukla A. An assessment of anthropometric indices and its association with NCDs among the older adults of India: evidence from LASI Wave-1. BMC Public Health. 2021;21(1):1357.

25. Cameron A, Magliano D, Shaw J, Zimmet P, Carstensen B, Alberti K, et al. The influence of hip circumference on the relationship between abdominal obesity and mortality. Intl J of Epi. 2012;41(2):484–94.

26. Latheef SAA, Subramanyam G, Reddy BM. Utility of anthropometric traits and indices in predicting the risk of coronary artery disease in the adult men of southern Andhra Pradesh. Indian Heart J. 2018;70(3):S133–9.

27. Nishtar S, Wierzbicki A, Lumb P, Lambert-Hammill M, Turner C, Crook M, et al. Waist-hip ratio and low HDL predict the risk of coronary artery disease in Pakistanis. Current Medical Research and Opinion. 2008;20(1):55–62.

28. Patil S, Joshi R, Gupta G, Reddy M, Pai M, Kalantri S. Risk factors for acute myocardial infarction in a rural population of central India: A hospital-based case–control study. Nat Med J India. 2004;17(4).

29. Prabhakar S, Suravarapu S, Mathai D, Renangi S, Challa S. Risk factors for stroke in rural population of Telangana State of India, an unmatched case-control study. J Neurosci Rural Pract. 2020;11(3):448–53.

30. Shah M, Mazumdar V, Patel S, Baxi R, Shringarpure K. A case control study of risk factors of coronary heart disease among patients admitted at tertiary hospital in western India. Australasian Medical Journal. 2017;10(05):381–8.

31. Borenstein M, Hedges L, Higgins J, Rothstein H. Introduction to Meta-Analysis. 2nd ed. John Wiley & Sons; 2009.

32. Higgins J, Green S, editors. Cochrane Handbook for Systematic Reviews of Interventions Verison 5.1.0 [Internet]. 2011. Available from: http://handbook-5-1.cochrane.org/.

33. DerSimonian R, Laird N. Meta-analysis in clinical trials. Controlled Clinical Trials. 1986;7(3):177–88.

For peer review only - http://bmjopen.bmj.com/site/about/guidelines.xhtml

PRISMA 2020 Checklist

Section and Topic	Item #	Checklist item	Location where item is reported
**TITLE**			
Title	1	Identify the report as a systematic review.	Page 1
**ABSTRACT**			
Abstract	2	See the PRISMA 2020 for Abstracts checklist.	Pages 2-3
**INTRODUCTION**			
Rationale	3	Describe the rationale for the review in the context of existing knowledge.	Pages 5-6
Objectives	4	Provide an explicit statement of the objective(s) or question(s) the review addresses.	Page 6
**METHODS**			
Eligibility criteria	5	Specify the inclusion and exclusion criteria for the review and how studies were grouped for the syntheses.	Page 7
Information sources	6	Specify all databases, registers, websites, organisations, reference lists and other sources searched or consulted to identify studies. Specify the date when each source was last searched or consulted.	Page 6
Search strategy	7	Present the full search strategies for all databases, registers, and websites, including any filters and limits used.	Supplement S1
Selection process	8	Specify the methods used to decide whether a study met the inclusion criteria of the review, including how many reviewers screened each record and each report retrieved, whether they worked independently, and if applicable, details of automation tools used in the process.	Page 7
Data collection process	9	Specify the methods used to collect data from reports, including how many reviewers collected data from each report, whether they worked independently, any processes for obtaining or confirming data from study investigators, and if applicable, details of automation tools used in the process.	Page 7
Data items	10a	List and define all outcomes for which data were sought. Specify whether all results that were compatible with each outcome domain in each study were sought (e.g. for all measures, time points, analyses), and if not, the methods used to decide which results to collect.	Pages 6-7
	10b	List and define all other variables for which data were sought (e.g. participant and intervention characteristics, funding sources). Describe any assumptions made about any missing or unclear information.	Pages 6-7
Study risk of bias assessment	11	Specify the methods used to assess risk of bias in the included studies, including details of the tool(s) used, how many reviewers assessed each study and whether they worked independently, and if applicable, details of automation tools used in the process.	Pages 7-8
Effect measures	12	Specify for each outcome the effect measure(s) (e.g. risk ratio, mean difference) used in the synthesis or presentation of results.	Page 7
Synthesis methods	13a	Describe the processes used to decide which studies were eligible for each synthesis (e.g. tabulating the study intervention characteristics and comparing against the planned groups for each synthesis (item #5)).	N/A
	13b	Describe any methods required to prepare the data for presentation or synthesis, such as handling of missing summary statistics, or data conversions.	Pages 8-9
	13c	Describe any methods used to tabulate or visually display results of individual studies and syntheses.	Page 8
	13d	Describe any methods used to synthesize results and provide a rationale for the choice(s). If meta-analysis was performed, describe the model(s), method(s) to identify the presence and extent of statistical heterogeneity, and software package(s) used.	Pages 8-9
	13e	Describe any methods used to explore possible causes of heterogeneity among study results (e.g. subgroup analysis, meta-regression).	Pages 8-9
	13f	Describe any sensitivity analyses conducted to assess robustness of the synthesized results.	N/A
Reporting bias assessment	14	Describe any methods used to assess risk of bias due to missing results in a synthesis (arising from reporting biases).	Pages 7-8
Certainty	15	Describe any methods used to assess certainty (or confidence) in the body of evidence for an outcome.	N/A

For peer review only - http://bmjopen.bmj.com/site/about/guidelines.xhtml

PRISMA 2020 Checklist

Section and Topic	Item #	Checklist item	Location where item is reported
assessment			
**RESULTS**			
Study selection	16a	Describe the results of the search and selection process, from the number of records identified in the search to the number of studies included in the review, ideally using a flow diagram.	Pages 9-10
	16b	Cite studies that might appear to meet the inclusion criteria, but which were excluded, and explain why they were excluded.	Figure 1
Study characteristics	17	Cite each included study and present its characteristics.	Pages 9-10; Supplement S2 and S3
Risk of bias in studies	18	Present assessments of risk of bias for each included study.	Page 16; Supplement S4a, S4b, S4c
Results of individual studies	19	For all outcomes, present, for each study: (a) summary statistics for each group (where appropriate) and (b) an effect estimate and its precision (e.g. confidence/credible interval), ideally using structured tables or plots.	Supplement S2 and S3
Results of syntheses	20a	For each synthesis, briefly summarise the characteristics and risk of bias among contributing studies.	Page 16
	20b	Present results of all statistical syntheses conducted. If meta-analysis was done, present for each the summary estimate and its precision (e.g. confidence/credible interval) and measures of statistical heterogeneity. If comparing groups, describe the direction of the effect.	Pages 10-11; Figures 2, 3, 4; Supplement S5, S6, S7, S8
	20c	Present results of all investigations of possible causes of heterogeneity among study results.	N/A
	20d	Present results of all sensitivity analyses conducted to assess the robustness of the synthesized results.	N/A
Reporting biases	21	Present assessments of risk of bias due to missing results (arising from reporting biases) for each synthesis assessed.	Page 12; Figure 5
Certainty of evidence	22	Present assessments of certainty (or confidence) in the body of evidence for each outcome assessed.	N/A
**DISCUSSION**			
Discussion	23a	Provide a general interpretation of the results in the context of other evidence.	Pages 12-14
	23b	Discuss any limitations of the evidence included in the review.	Pages 12-14
	23c	Discuss any limitations of the review processes used.	Pages 14-15
	23d	Discuss implications of the results for practice, policy, and future research.	Pages 12-15
**OTHER INFORMATION**			
Registration and protocol	24a	Provide registration information for the review, including register name and registration number, or state that the review was not registered.	Page 6
	24b	Indicate where the review protocol can be accessed, or state that a protocol was not prepared.	Page 6
	24c	Describe and explain any amendments to information provided at registration or in the protocol.	N/A
Support	25	Describe sources of financial or non-financial support for the review, and the role of the funders or sponsors in the review.	Page 20
Competing	26	Declare any competing interests of review authors.	Page 20

For peer review only

PRISMA 2020 Checklist

Section and Topic	Item #	Checklist item	Location where item is reported
interests			
Availability of data, code and other materials	27	Report which of the following are publicly available and where they can be found: template data collection forms; data extracted from included studies; data used for all analyses; analytic code; any other materials used in the review.	Page 20

From: Page MJ, McKenzie JE, Bossuyt PM, Boutron I, Hoffmann TC, Mulrow CD, et al. The PRISMA 2020 statement: an updated guideline for reporting systematic reviews. BMJ 2021;372:n71. doi: 10.1136/bmj.n71

For more information, visit: http://www.prisma-statement.org/

For peer review only
